# PM2.5 promotes lung cancer progression through activation of the AhR‐TMPRSS2‐IL18 pathway

**DOI:** 10.15252/emmm.202217014

**Published:** 2023-03-28

**Authors:** Tong‐Hong Wang, Kuo‐Yen Huang, Chin‐Chuan Chen, Ya‐Hsuan Chang, Hsuan‐Yu Chen, Chuen Hsueh, Yi‐Tsen Liu, Shuenn‐Chen Yang, Pan‐Chyr Yang, Chi‐Yuan Chen

**Affiliations:** ^1^ Tissue Bank, Chang Gung Memorial Hospital at Linkou Taoyuan Taiwan; ^2^ Graduate Institute of Health Industry Technology and Research Center for Chinese Herbal Medicine, College of Human Ecology Chang Gung University of Science and Technology Taoyuan Taiwan; ^3^ Liver Research Center, Department of Hepato‐Gastroenterology Chang Gung Memorial Hospital at Linkou Taoyuan Taiwan; ^4^ Graduate Institute of Natural Products Chang Gung University Taoyuan Taiwan; ^5^ Department of Clinical Laboratory Sciences and Medical Biotechnology, College of Medicine National Taiwan University Taipei Taiwan; ^6^ Institute of Statistical Science Academia Sinica Taipei Taiwan; ^7^ Department of Anatomic Pathology Chang Gung Memorial Hospital, Linko Taoyuan Taiwan; ^8^ Institute of Biomedical Sciences Academia Sinica Taipei Taiwan; ^9^ Department of Internal Medicine National Taiwan University Hospital and National Taiwan University College of Medicine Taipei Taiwan; ^10^ Genomics Research Center Academia Sinica Taipei Taiwan

**Keywords:** AhR, EGFR, lung cancer, PM2.5, TMPRSS2, Cancer, Respiratory System

## Abstract

Particulate matter 2.5 (PM2.5) is a risk factor for lung cancer. In this study, we investigated the molecular mechanisms of PM2.5 exposure on lung cancer progression. We found that short‐term exposure to PM2.5 for 24 h activated the EGFR pathway in lung cancer cells (EGFR wild‐type and mutant), while long‐term exposure of lung cancer cells to PM2.5 for 90 days persistently promoted EGFR activation, cell proliferation, anchorage‐independent growth, and tumor growth in a xenograft mouse model in EGFR‐driven H1975 cancer cells. We showed that PM2.5 activated AhR to translocate into the nucleus and promoted EGFR activation. AhR further interacted with the promoter of TMPRSS2, thereby upregulating TMPRSS2 and IL18 expression to promote cancer progression. Depletion of TMPRSS2 in lung cancer cells suppressed anchorage‐independent growth and xenograft tumor growth in mice. The expression levels of TMPRSS2 were found to correlate with nuclear AhR expression and with cancer stage in lung cancer patient tissue. Long‐term exposure to PM2.5 could promote tumor progression in lung cancer through activation of EGFR and AhR to enhance the TMPRSS2‐IL18 pathway.

## Graphical abstract

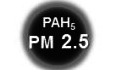

**The paper explained**

**Problem**

Particulate matter 2.5 (PM2.5) is a known risk factor for lung cancer; however, the molecular mechanisms triggered by exposure to PM2.5 and affecting cancer progression are unclear.

**Results**

In this study, lung cancer cells exposed to PM2.5 for 90 days were used as a cellular model of the effects of long‐term exposure to PM2.5 on lung cancer. In mice, tumor progression was enhanced by long‐term exposure to PM2.5. The molecular mechanism identified may include (i) activation of EGFR signaling and (ii) activation of AhR to upregulate TMPRSS2, which in turn upregulates its downstream targets, such as IL18.

**Impact**

Long‐term exposure to PM2.5 could promote tumor progression in lung cancer through activation of EGFR and AhR to enhance the TMPRSS2‐IL18 pathway.

## Introduction

Air pollution is contamination of the indoor or outdoor atmosphere by chemical, physical, or biological agents, and is one of the greatest environmental risks to human health. The combined effects of ambient (outdoor) and household air pollution were estimated by World Health Organization (WHO) to cause about 7 million premature deaths every year (Pérez Velasco & Jarosińska, 2022). The major components of air pollution are particulate matter (PM). Short‐term or long‐term exposure to PM is known to cause adverse health effects, including cardiovascular and respiratory disease, and cancers (Bazyar *et al*, 2019).

Particulate matter can be divided into PM10 or PM2.5 depending on whether the diameter of the PM particles is less than 10 or 2.5 μm. PM10 is mostly deposited in the nasal cavity and upper respiratory tract, while PM2.5 can enter the lower respiratory tract into the alveoli and enter the bloodstream (Sicard *et al*, 2019). PM2.5 enters the human body mainly through inhalation, thus causing various pathological effects on the respiratory system, such as chronic obstructive pulmonary disease, asthma, and multidrug resistance after tuberculosis treatment (Xing *et al*, 2016; Jeong *et al*, 2019; Yao *et al*, 2019). PM2.5 is composed of inorganic, organic, and biological compounds such as polycyclic aromatic hydrocarbons (PAHs), endotoxins, and fungi (Harrison & Yin, 2000). Among them, PAHs are the main components of PM2.5, accounting for approximately 80% (Javed *et al*, 2019). PAHs are mainly derived from fossil fuels such as petroleum and coal or man‐made organic chemicals. PAHs are highly lipophilic and can enter cells directly through the cell membrane. PAHs can bind to cytoplasmic aryl hydrocarbon receptor (AhR) and then translocate to the nucleus to promote the expression of the cytochrome P450 family, which assists in the metabolism of carcinogens and environmental pollutants (Romagnolo *et al*, 2010). In addition, PAHs are known to photochemically react with sulfur oxides, nitrogen oxides, and ozone in the air to form secondary pollutants that are carcinogenic and genotoxic to the human body (Henkler *et al*, 2012).

Long‐term exposure to air pollution is one of the causes of the high incidence of lung cancer (Pun *et al*, 2017). The inhalation of substances containing PAHs, such as PM2.5, is known to increase the risk of lung cancer (Singh *et al*, 2018). Exposure to PM2.5 can cause DNA damage and activation of AhR, epidermal growth factor receptor (EGFR), and the immune system, leading to tumorigenesis (Chen *et al*, 2020). Recent studies have shown that PM2.5 exposure promotes the expression of transmembrane serine protease 2 (TMPRSS2) (Li *et al*, 2021), a type 2 transmembrane serine protease family (TTSP) that is known to promote SARS‐CoV‐2 infection and is also involved in the regulation of carcinogenesis (Martin & List, 2019; Hoffmann *et al*, 2020). Although many epidemiological studies have confirmed that PM2.5 is a risk factor for the occurrence of lung cancer (Al‐Hamdan *et al*, 2017; Tseng *et al*, 2019), the definitive role of PM2.5 in the development of lung cancer remains largely unknown. In this study, we show that long‐term exposure of lung cancer cells to PM2.5 can activate AhR to promote the expression of TMPRSS2 and IL18 and promote lung cancer progression.

## Results

### Effects of short‐ and long‐term exposure to PM2.5 on cell proliferation, EGFR signaling, and anchorage‐independent growth of lung cancer cells

**Figure 1:**
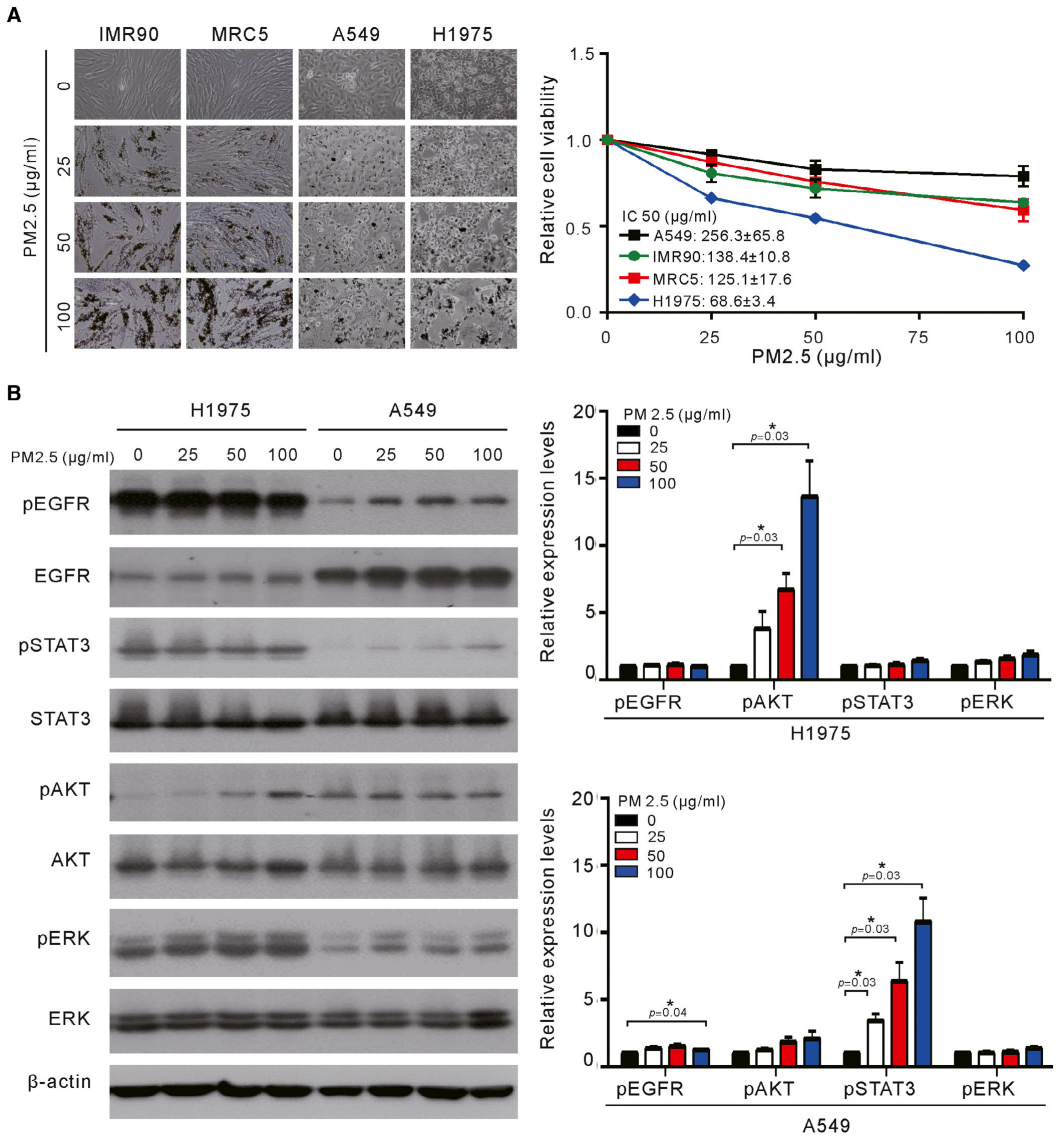
Effects of short‐term exposure to PM2.5 on cell viability and EGFR signaling in lung cancer cells Normal lung cells (IMR90 and MRC5) and lung cancer cells (A549 and H1975) were treated with various concentrations of PM2.5 for 24 h, and cell viability was assessed by a Trypan blue assay. The values are the mean ± SD of three independent experiments.H1975 and A549 cells were treated with various concentrations of PM2.5 for 24 h, and their cell lysates were analyzed for phosphor‐ERK (pERK), phosphor‐AKT (pAKT), phosphor‐STAT3 (pSTAT3), phosphor‐EGFR (pEGFR), ERK, AKT, STAT3, and EGFR by Western blotting. β‐actin served as the loading control. The results shown are from one of three similar experiments (left panel). The relative expression levels of pEGFR, pAKT, pSTAT3, and pERK were quantified by normalizing with β‐actin and are shown in the right panel. The values are the mean ± SD of three independent experiments. *P < 0.05, as analyzed with one‐sample *t*‐test and compared with untreated cells. Source data are available online for this figure.

The cytotoxic effects of PM2.5 on normal lung and lung cancer cells were evaluated by short‐term 24‐h exposure using the normal lung fibroblast cell lines MRC5 and IMR90 and lung cancer cell lines A549 (EGFR wild‐type) and H1975 (EGFR L858R+T790M). As shown in Fig 1A, short‐term exposure to PM2.5 appears to produce a greater cytotoxic effect on H1975 cells than on IMR90 (*P* < 0.001), MRC5 (*P* < 0.001), and A549 (*P* < 0.001) cells. The IC50s for IMR90, MRC5, and A549 were over 100 μg/ml, while the IC50 of H1975 was 68.6 ± 3.46 μg/ml (Fig 1A). As EGFR activation is the main driver of lung cancer (Tumbrink *et al*, 2021), the effects of exposure to PM2.5 on EGFR signaling were examined next. As shown in Fig 1B, PM2.5 induced the activation of phosphor (p)‐EGFR, and pSTAT3 in A549 cells but induced the activation of only pAKT in H1975 cells.

**Figure 2:**
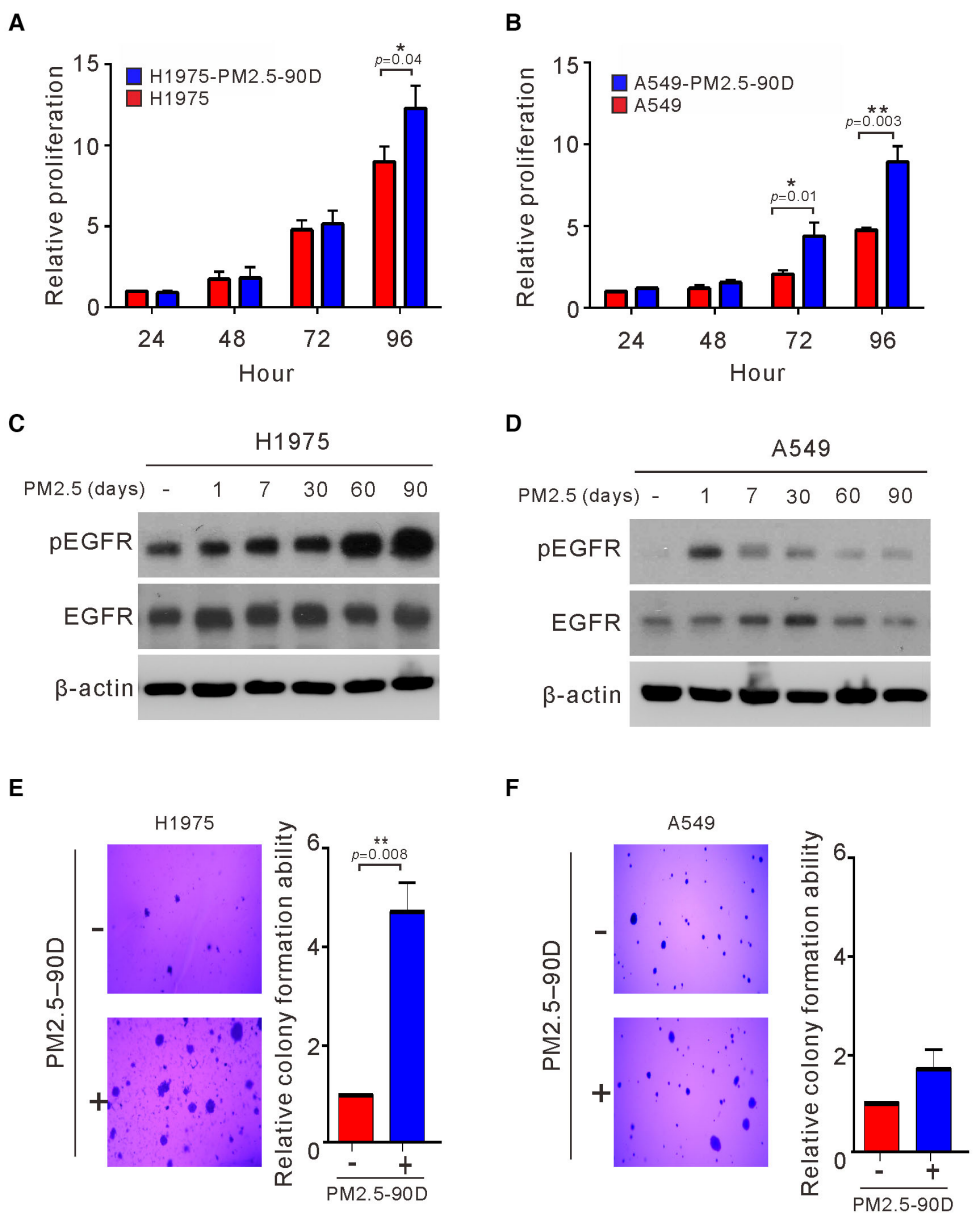
Effects of long‐term exposure to PM2.5 on cell proliferation, EGFR activation, and anchorage‐independent growth of lung cancer cells A, BH1975 and A549 cells were treated with PM2.5 at 50 μg/ml for 90 days, and the proliferation of the treated cells was assessed by Trypan blue assays.C, DH1975 and A549 cells were exposed to PM2.5 at 50 μg/ml for different lengths of time, and the cell lysates of treated cells were assessed for phosphorylated EGFR (pEGFR) and EGFR by Western blotting. β‐actin served as the loading control.E, FH1975 and A549 cells were treated with PM2.5 at 50 μg/ml for 90 days. The anchorage‐independent growth was assessed by a soft agar colony formation assay. The number of colonies was scored, and the data are presented as the relative colony formation ability. Data information: The data shown are the means ± SDs from three independent experiments. *P < 0.05 and **P < 0.01, compared with untreated control cells. The results shown in (C and D) are from one of three similar experiments. (A and B) *P*‐values were determined by two‐sample *t*‐test. (E and F) *P*‐values were determined by one‐sample *t*‐test. Source data are available online for this figure.

**Figure EV1:**
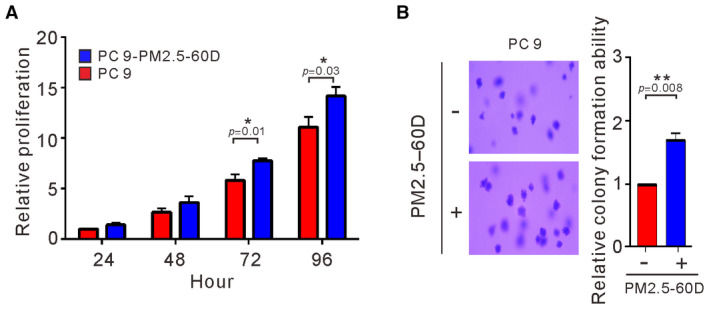
Effects of long‐term exposure to PM2.5 on cell proliferation and anchorage‐independent growth of PC9 lung cancer cells A, BPC9 cells were treated with PM2.5 at 50 μg/ml for 60 days. The proliferation of treated cells was assessed by Trypan blue assay (A). The anchorage‐independent growth was assessed by a soft agar colony formation assay (B). The data shown are the means ± SDs from three independent experiments. *P < 0.05 and **P < 0.01, compared with untreated cells. (A) *P*‐values were determined by two‐sample *t*‐test. (B) *P*‐values were determined by one‐sample *t*‐test. Source data are available online for this figure.

To examine the effects of long‐term exposure on cancer cells, we cultured A549 and H1975 cells in the presence of PM2.5 at 50 μg/ml for 90 days before analysis of proliferation and EGFR activation. As shown in Fig 2A and B, long‐term exposure to PM2.5 appeared to increase the proliferation of both EGFR wild‐type and EGFR mutant cancer cells (*P* < 0.05). The effects of long‐term exposure on EGFR activation were also examined at different times of continuous exposure to PM2.5 at 50 μg/ml in lung cancer cells. As shown in Fig 2C, the expression of pEGFR in H1975 cells was continuously upregulated at different times of exposure to PM2.5 and reached the highest level after 90 days. Exposure to PM2.5 also upregulated the expression of activated pEGFR in A549 cells (Fig 2D) but resulted in the highest activation on Day 1, followed by a show decrease to lower levels after longer exposure times. Next, we examined the effects of long‐term exposure to PM2.5 on anchorage‐independent growth in H1975 and A549 cells. As shown in Fig 2E and F, long‐term exposure to PM2.5 substantially increased the ability of H1975 and A549 cells to undergo anchorage‐independent growth, although the increased pattern did not reach statistical significance in A549 cells. Increased ability to proliferate and to undergo anchorage‐independent growth was also observed in PC9 lung cancer cells exposed to PM2.5 for 60 days (Fig EV1). Thus, long‐term exposure to PM2.5 increased the ability of cancer cells to proliferate and undergo anchorage‐independent growth.

### Long‐term exposure of lung cancer cells to PM2.5 enhances tumorigenicity *in vivo*

**Figure 3:**
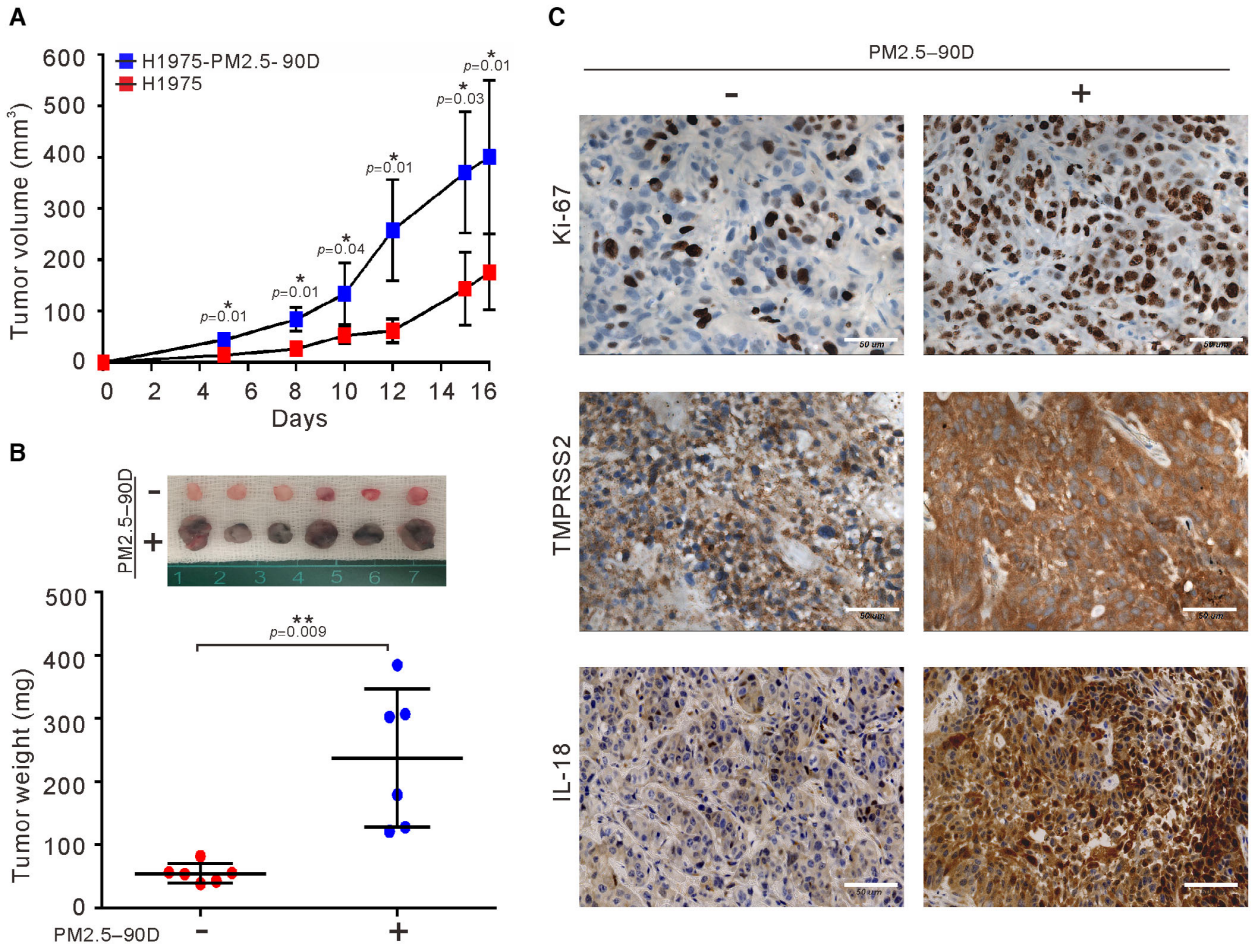
Effects of long‐term exposure to PM2.5 on tumor growth of lung cancer cells *in vivo*. H1975 cells were exposed to 50 μg/ml PM2.5 for 90 days. Both unexposed and exposed cells were injected subcutaneously into the flank of each mouse (*n* = 6 per group) A, BThe tumor volume and excised tumor weight were measured. The sizes of tumors excised from each group are shown at the top of (B).CIHC staining of excised tumors for Ki‐67, IL18, and TMPRSS2 is shown in (C). Scale bars, 50 μm. Data information: The results shown in (A) and (B) are presented as the means ± SDs of six mice. *P < 0.05 and **P < 0.01, compared with untreated group. (A) *P*‐values were determined by two‐way repeated measures ANOVA with pairwise comparison of *post hoc* analysis with Benjamini–Hochberg (BH) correction. (B) *P*‐values were determined by two‐sample *t*‐test. Source data are available online for this figure.

A previous study showed that A549 cells treated with PM2.5 for 10 days have an increased ability to migrate and invade and induce cancer stem cell properties (Wang *et al*, 2019). Here, we examined the effects of long‐term exposure to PM 2.5 on H1975 cells. To determine whether the increased ability to proliferate and to undergo anchorage‐independent growth by long‐term exposure to PM2.5 may indeed impact tumor growth *in vivo*, we subcutaneously injected unexposed and PM2.5‐exposed H1975 cells into the flanks of nude mice, and the tumor volumes were determined twice a week. At 16 days after injection, the mice were euthanized. As shown in Fig 3A and B, the tumor growth in the mice injected with PM2.5‐exposed H1975 cells was considerably faster than that in the mice injected with untreated H1975 cells. Consistent with this finding, the levels of Ki‐67, TMPRSS2, and IL18 were greatly increased in the tumors derived from the PM2.5‐exposed cells compared to the tumors derived from the untreated cells (Fig 3C). These results indicate that long‐term exposure to PM2.5 enhanced the tumorigenic abilities of lung cancer cells *in vivo*.

### Long‐term exposure to PM2.5 elevates the expression of TMPRSS2 by activating AhR in lung cancer cells

**Figure 4:**
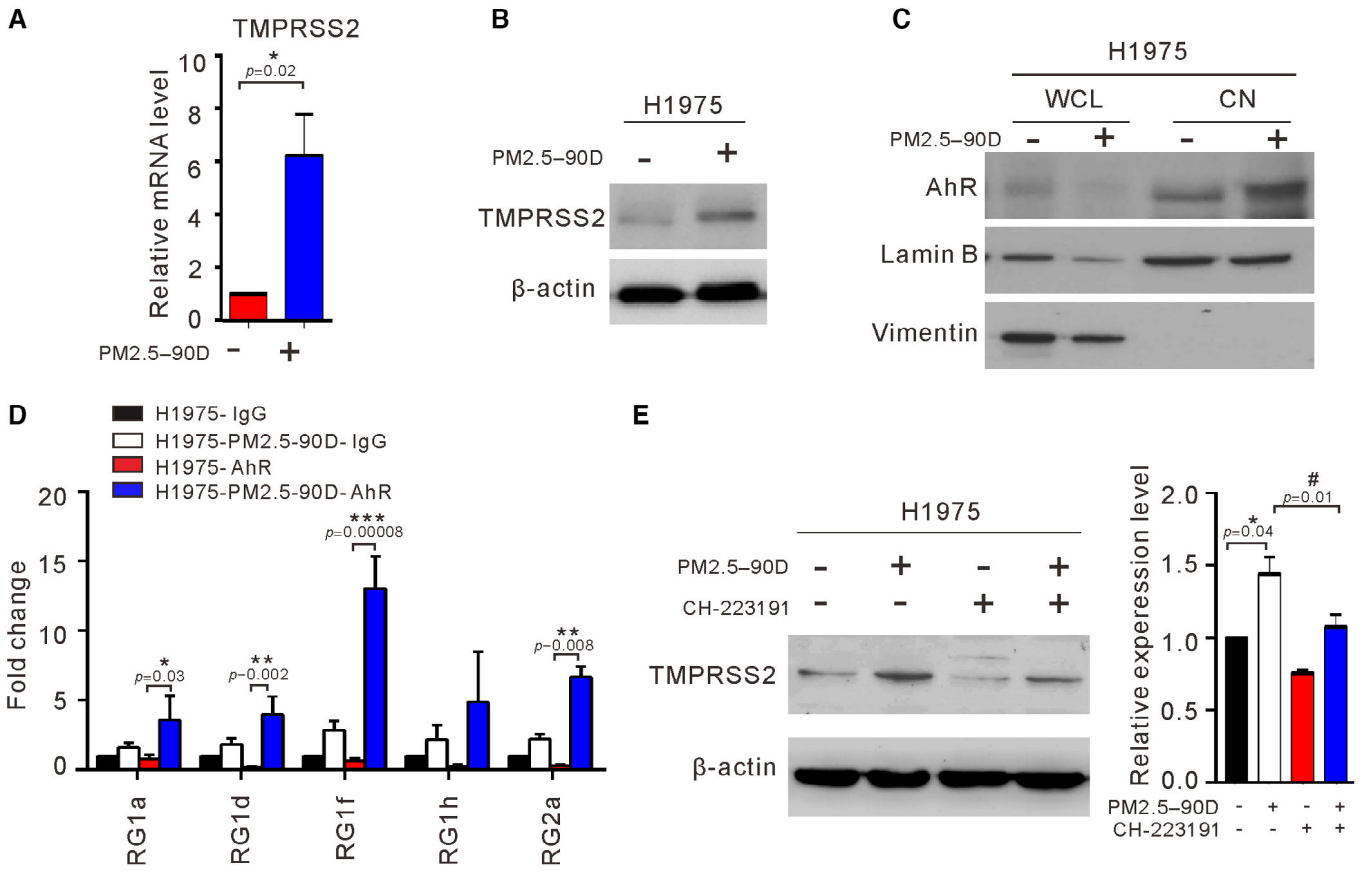
Effects of long‐term exposure to PM2.5 on the AhR‐TMPRSS2 axis in lung cancer cells A, BH1975 cells were treated with 50 μg/ml PM2.5 for 90 days, and the expression levels of TMPRSS2 mRNA and protein were determined by real‐time RT–PCR (A) and Western blotting (B), respectively.CThe proteins from whole cell lysates (WCL) and nuclear fractions (CN) were analyzed for AhR, lamin B (nuclei marker), and vimentin (cytoplasm marker) by Western blotting.DChIP–qPCR analysis of AhR binding to the promoter of the TMPRSS2 locus. The chromatin of untreated or treated H1975 cells was immunoprecipitated using AhR antibody. Precipitated genomic DNA was amplified for the five sites (RG1a, 1d, 1f, 1 h, and 2a) in the proximal promoter of the TMPRSS2 locus by real‐time PCR. Data were normalized to the input and expressed as “Fold change” relative to the IgG control of H1975 cells.EWestern blot analysis of TMPRSS2 expression in cells treated with CH223191 at 10 μM for 48 h. The relative expression level of TMPRSS2 was quantified by normalizing with β‐actin and is shown in the right panel. Data information: The data shown represent the mean ± SD of three independent experiments. *P < 0.05 and **P < 0.01, and ***P < 0.001, compared with untreated cells. ^#^ *P* < 0.05, compared with PM2.5‐treated cells. The results shown in (B, C, and E) are from one of three similar experiments. (A) *P*‐values were determined by one‐sample *t*‐test. (D) One‐way ANOVA and Tukey's multiple‐comparisons test of *post hoc* analysis were used when homogeneity of variance across groups (RG1a, RG1d, and RG1f) occurred; Welch's ANOVA and Games–Howell test of *post hoc* analysis were used when variance across groups was not equal (RG1h and RG2a). (E) *P*‐values were determined by one‐sample *t*‐test. Source data are available online for this figure.

**Figure EV2:**
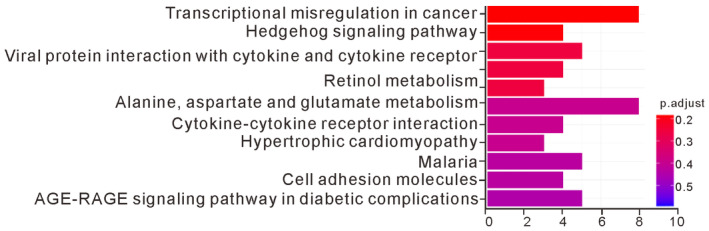
The top 10 enriched biological processes in long‐term exposure to PM2.5 H1975 cells were exposed to 50 μg/ml PM2.5 for 90 days, and total RNA was subjected to whole‐transcriptome analysis as described in the Materials and Methods. Functional classification of the differentially expressed genes in PM2.5‐treated H1975 cells, as assessed using ingenuity pathway analysis.Source data are available online for this figure.

We used whole‐transcriptome sequencing to further explore the mechanism of action affected by long‐term exposure to PM2.5 in H1975 cells. A total of 292 genes exhibited differential expression over twofold greater or lesser in the PM2.5‐treated H1975 cells. These differentially expressed genes were subjected to ingenuity pathway analysis, which revealed that PM2.5 affected transcriptional dysregulation in cancer, the hedgehog signaling pathway, viral protein interactions with cytokine and cytokine receptors, retinol metabolism, alanine, aspartate, and glutamate metabolism, cytokine–cytokine receptor interactions, hypertrophic cardiomyopathy, malaria, cell adhesion molecules, and the AGE‐RAGE signaling pathway in diabetic complications (Fig EV2). The 10 differentially expressed genes with the highest scores are listed in Table 1. Among them, TMPRSS2 is a known oncogene that promotes tumor progression and metastasis in prostate cancer (Stone, 2017; Ko *et al*, 2020). Previous studies have shown that PM2.5 exposure upregulates the expression of TMPRSS2 in pulmonary fibroblasts (Li *et al*, 2021). We thus selected TMPRSS2 for further study. To address the potential importance of TMPRSS2 upregulation in PM2.5‐treated H1975 cells, we first examined whether PM2.5 induced TMPRSS2 upregulation by quantitative real‐time RT–PCR and Western blotting. As shown in Fig 4A and B, TMPRSS2 was increased to a higher level in the PM2.5‐treated 1975 cells than in the untreated cells.

**Table 1:** Identities of the 10 highest‐scored genes differentially expressed in PM2.5‐treated H1975 cells.

Rank	Gene name	Gene description	NCBI gene ID
1	NME1‐NME2	NME1‐NME2 readthrough [Source: HGNC Symbol; Acc: HGNC: 33531]	654364
2	ZBTB9	Zinc finger and BTB domain containing 9 [Source: HGNC Symbol; Acc: HGNC: 28323]	221504
3	RNASE7	Ribonuclease A family member 7 [Source: HGNC Symbol; Acc: HGNC: 19278]	84659
4	HES7	Hes family bHLH transcription factor 7 [Source: HGNC Symbol; Acc: HGNC: 15977]	84667
5	BEX5	Brain‐expressed X‐linked 5 [Source: HGNC Symbol; Acc: HGNC: 27990]	340542
6	CCDC173	Coiled‐coil domain containing 173 [Source: HGNC Symbol; Acc: HGNC: 25064]	129881
7	FBP2	Fructose‐bisphosphatase 2 [Source: HGNC Symbol; Acc: HGNC: 3607]	8789
8	AKNAD1	AKNA domain containing 1 [Source: HGNC Symbol; Acc: HGNC: 28398]	254268
9	CPXM2	Carboxypeptidase X, M14 family member 2 [Source: HGNC Symbol; Acc: HGNC: 26977]	119587
10	TMPRSS2	Transmembrane protease, serine 2 [Source: HGNC Symbol; Acc: HGNC: 11876]	7113

**Figure EV3:**
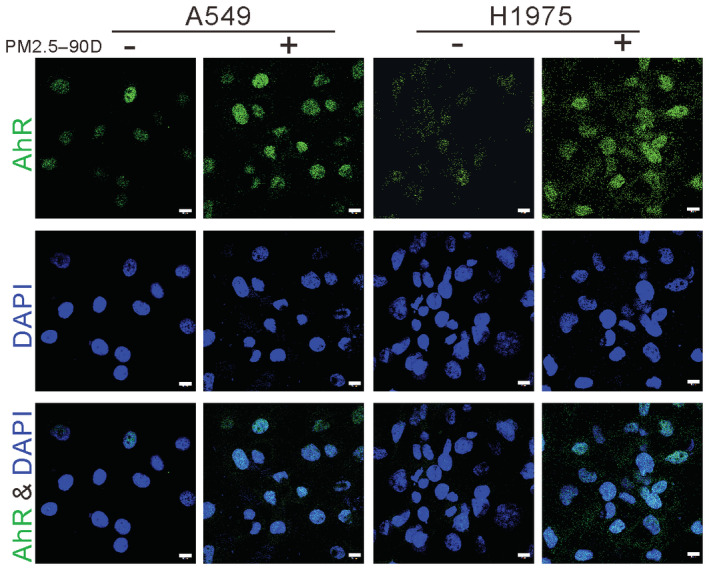
Immunofluorescence staining of AhR The subcellular distribution of AhR was assessed by immunofluorescence staining in H1975 and A549 cells treated with 50 μg/ml PM2.5 for 90 days. DAPI was used as a nuclear stain. Scale bar: 10 μm.Source data are available online for this figure.

Polycyclic aromatic hydrocarbons can activate AhR and then regulate the transcriptional expression of related genes (Tsay *et al*, 2013). Studies have shown that there are putative binding sites for AhR in the promoter of TMPRSS2 (Watzky *et al*, 2021). Therefore, we hypothesized that long‐term exposure to PM2.5 activates AhR translocation to the nucleus and then binds to the promoter of TMPRSS2. To test this hypothesis, we used nuclear fractionation and Western blot analysis to examine the effects of PM2.5 treatment on the cellular distribution of AhR in nuclear and whole‐cell lysates. As shown in Fig 4C, the levels of nuclear AhR were greatly increased in the PM 2.5‐exposed H1975 cells. To verify the nuclear localization of AhR, we performed immunofluorescence staining of AhR in A549 and H1975 cell lines. As shown in Fig EV3, elevated nuclear localization of AhR was evident in the PM2.5‐treated cells. To determine whether the increased nuclear AhR may be associated with enhanced binding at the promoter of TMPRSS2, we performed ChIP–qPCR analysis in the PM2.5‐treated and untreated H1975 cells. As shown in Fig 4D, AhR binding was enhanced at four distinct sites of the TMPRSS2 promoter in the PM2.5‐exposed H1975 cells. These results suggest that long‐term exposure to PM2.5 can activate AhR to enhance the expression of TMPRSS2 in lung cancer cells. Consistent with a role of AhR in the expression of TMPPSS2, we observed that the AhR antagonist CH223191 inhibited the expression of TMPRSS2 in PM2.5‐treated cells (Fig 4E).

### Effects of TMPRSS2 depletion on the anchorage‐independent growth and *in vivo* tumor growth of lung cancer cells

**Figure 5:**
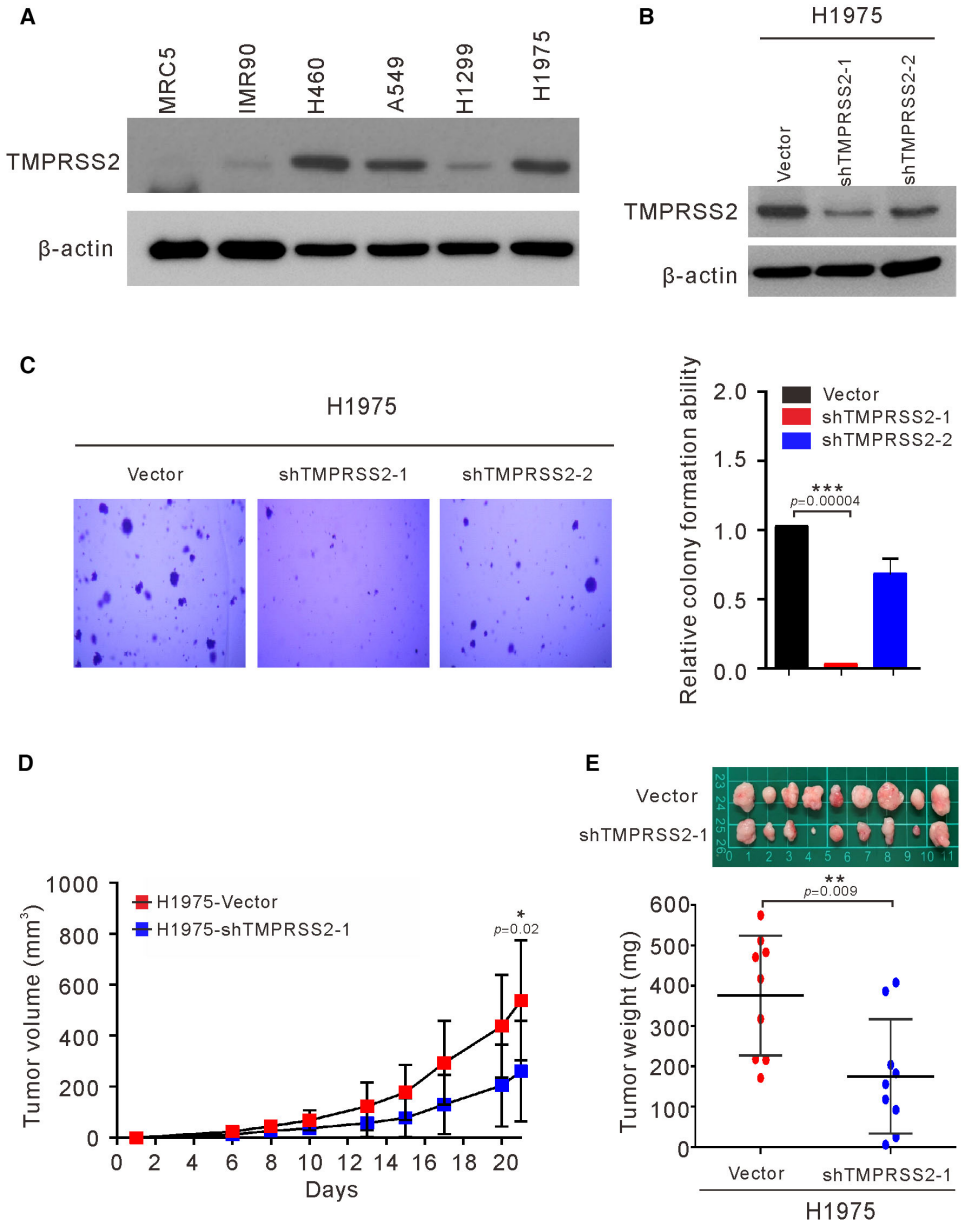
Effects of TMPRSS2 depletion on the anchorage‐independent growth and *in vivo* tumor growth of lung cancer cells AExpression of TMPRSS2 was examined by Western blots of four lung cancer cell lines (H460, A546, H1299, and H1975) and two normal fibroblasts (MRC5 and IMR90).B, CH1975 cells were infected with sh‐TMPRSS2 (sh‐TMPRSS2‐1 and sh‐TMPRSS2‐2) or empty vector (Vector). The stable clones of TMPRSS2 knockdown cells were analyzed for the expression of TMPRSS2 by Western blots (B) and their ability to perform anchorage‐independent growth in soft agar (C).D, EH1975‐shTMPRSS2‐1 cells were injected subcutaneously into mice (*n* = 9 per group), and the tumor growth of the implanted cells was measured (D). The excised tumors and their weights are shown in (E). Data information: The results shown in (A and B) are from one of three similar experiments. β‐actin was used as the loading control. The data shown in (C) represent the means ± SDs from three independent experiments. The results shown in (D) and (E) are presented as the means ± SDs of nine mice. *P < 0.05, **P < 0.01, and ***P < 0.001 compared with vector control. (C) *P*‐values were determined by one‐sample *t*‐test. (D and E) *P*‐values were determined by two‐sample *t*‐test. Source data are available online for this figure.

TMPRSS2 is associated with the development of prostate cancer and the promotion of SARS‐CoV‐2 infection of lung cells (Hoffmann *et al*, 2020). However, the biological function of TMPRSS2 in lung cancer is unclear, and the current research results are inconsistent (Wang *et al*, 2021; Schneider *et al*, 2022). To determine whether the expression level of TMPRSS2 is increased in lung cancer cells, we examined the endogenous protein level of TMPRSS2 in four established lung cancer cell lines and two normal lung fibroblasts by Western blotting. As shown in Fig 5A, a high level of TMPRSS2 protein was detected in three lung cancer cell lines (H460, A549, and H1975). A low level of TMPRSS2 was also detected in H1299 lung cancer cells and normal IMR90 fibroblasts but was undetectable in normal MRC5 fibroblasts. To determine whether the level of TMPRSS2 may impact tumorigenicity in lung cancer cells, we examined the effect of stable depletion of TMPRSS2 on anchorage‐independent growth of H1975 cells. As shown in Fig 5B and C, the levels of TMPRSS2 and anchorage‐independent growth were greatly reduced in the cells stably expressing shTMPRSS2‐1. The cells stably expressing shTMPRSS2‐2 had only a slight reduction in the expression of TMPRSS2 and a slight inhibition of anchorage‐independent growth. To determine whether TMPRSS2 affects lung cancer cell tumorigenesis *in vivo*, we performed a xenotransplantation assay in nude mice. As shown in Fig 5D, the growth of the shTMPRSS2‐1 H1975 cell‐derived xenograft tumors was slower than that of the vector control cell‐derived xenograft tumors. Similarly, the average weight of the excised shTMPRSS2‐1 tumors was lower than that of the vector control tumors (Fig 5E). These results show that the downregulation of TMPRSS2 inhibited lung cancer cell tumor growth *in vitro* and *in vivo*.

### Transcriptomic profiling of TMPRSS2‐regulated genes in H1299 cells

**Figure 6:**
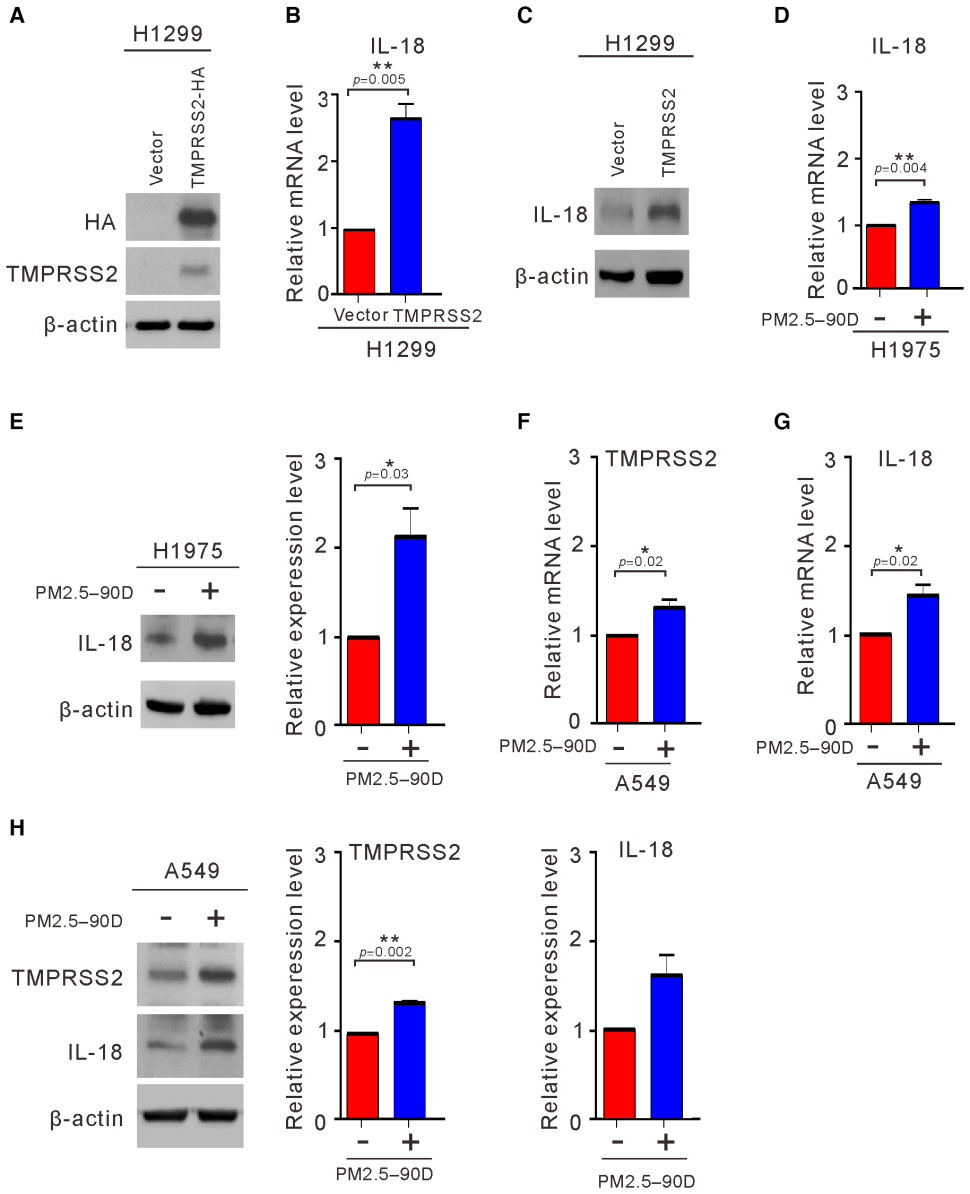
Effects of TMPRSS2 overexpression on the induction of IL18 A–CH1299 cells were transfected with TMPRSS2‐HA or the empty vector (Vector). After 48 h, the transfected cells were assayed for the expression of TMPRSS2 (A) and the induction of IL18 by qRT–PCR (B) and Western blotting (C).D–HThe expression level of IL18 was examined in H1975 cells (D, E) and A549 cells (F–H) exposed to PM2.5 at 50 μg/ml for 90 days by qRT–PCR (D, F, and G) and Western blotting (E and H). Data information: The results shown in (A, C, E, and H) are from one of three similar experiments. β‐actin was used as the loading control. The data shown in (E and H) were normalized to β‐actin from three independent experiments. The data shown in (B, D–H) represent the means ± SDs from three independent experiments; *P < 0.05 and **P < 0.01, as analyzed with one‐sample *t*‐test and compared with vector control or untreated cells. Source data are available online for this figure.

**Figure EV4:**
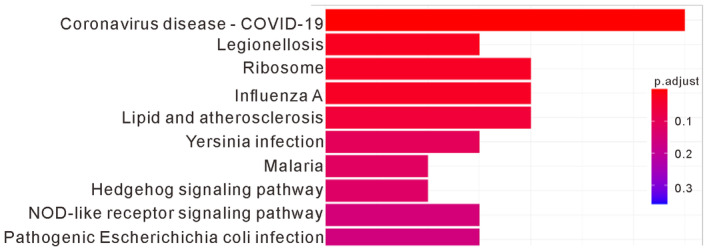
The top 10 enriched biological processes in TMPRSS2 overexpression H1299 cells were transfected with TMPRSS2‐HA or the empty vector (Vector). After 48 h, the transfected cells were subjected to whole‐transcriptome analysis. Functional classification of the differentially expressed genes in TMPRSS2‐overexpressing H1299 cells, as assessed using ingenuity pathway analysis.Source data are available online for this figure.

To explore the molecular effects of TMPRSS2 on lung cancer cells, we examined the effects of overexpressing TMPRSS2 using H1299 cells, which express low levels of endogenous TMPRSS2 (Fig 5A). First, we employed whole‐transcriptome sequencing to compare the gene expression profiles of H1299 cells transfected with or without TMPRSS2 (Fig 6A). A total of 169 putative genes were identified in the H1299 cells that overexpressed TMPRSS2 and showed a significant twofold difference in the expression level. With ingenuity pathway analysis, the pathways that were enriched in the TMPRSS2‐overexpressing H1299 cells were determined and included coronavirus disease–COVID‐19, legionellosis, ribosome, influenza A, lipid and atherosclerosis, *Yersinia* infection, malaria, hedgehog signaling pathway, NOD‐like receptor signaling pathway, and pathogenic *Escherichia coli* infection (Fig EV4). Table 2 shows the top 10 upregulated genes in the TMPRSS2‐overexpressing H1299 cells. Among them, IL18 has been shown to play a role in carcinogenesis and tumor progression (Zitvogel *et al*, 2012). IL18 expression showed positive correlations with TMPRSS2 in various human tissues (Cao *et al*, 2021). To further confirm the correlation of IL18 upregulation by TMPRSS2 overexpression, we performed RT–qPCR and Western blot analysis to detect IL18 mRNA and protein levels in the TMPRSS2‐overexpressing H1299 cells. As shown in Fig 6B and C, the IL18 mRNA and protein levels were increased in the TMPRSS2‐overexpressing H1299 cells. Interestingly, we found that the mRNA and protein levels of IL18 and TMPRSS2 were also increased in the H1975 cells and A549 cells exposed to PM2.5 for 90 days, although the protein‐increased level of IL18 did not reach the statistical significance in A549 cells (Fig 6D–H). These results suggest that enhanced expression of IL18 is correlated with increased expression of TMPRSS2.

**Table 2:** Identities of the 10 highest‐scored genes differentially expressed in TMPRSS2‐overexpressing H1299 cells.

Rank	Gene name	Gene description	NCBI gene ID
1	RPL17‐C18orf32	RPL17‐C18orf32 readthrough [Source: HGNC Symbol; Acc: HGNC: 44661]	100526842
2	IL18	Interleukin 18 [Source: HGNC Symbol; Acc: HGNC: 5986]	3606
3	OLAH	Oleoyl‐ACP hydrolase [Source: HGNC Symbol; Acc: HGNC: 25625]	55301
4	TMPRSS2	Transmembrane serine protease 2 [Source: HGNC Symbol; Acc: HGNC: 11876]	7113
5	HSPA6	Heat shock protein family A (Hsp70) member 6 [Source: HGNC Symbol; Acc: HGNC: 5239]	3310
6	GCOM1	GRINL1A complex locus 1 [Source: HGNC Symbol; Acc: HGNC: 26424]	145781
7	RPL36A‐HNRNPH2	RPL36A‐HNRNPH2 readthrough [Source: HGNC Symbol; Acc: HGNC: 48349]	100529097
8	GAGE12D	G antigen 12D [Source: HGNC Symbol; Acc: HGNC: 31904]	100132399
9	CXCL8	C‐X‐C motif chemokine ligand 8 [Source: HGNC Symbol; Acc: HGNC: 6025]	3576
10	VEGFD	Vascular endothelial growth factor D [Source: HGNC Symbol; Acc: HGNC: 3708]	2277

### Clinical correlation of TMPRSS2 expression with nuclear AhR, IL18, and overall cancer staging in human lung cancer specimens

**Figure 7:**
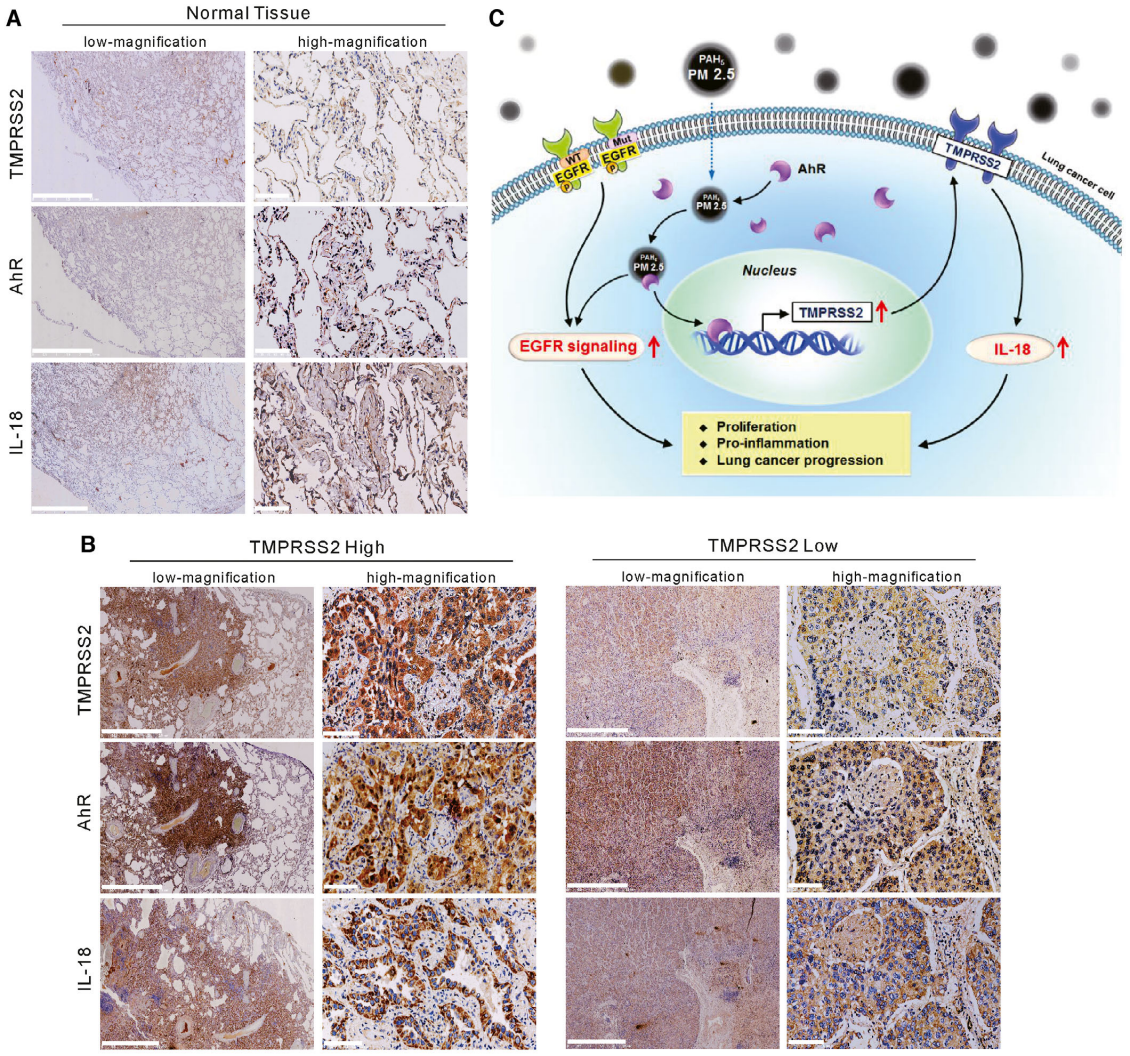
Expression levels of TMPRSS2 and nuclear AhR in normal and cancer lung tissues IHC staining of TMPRSS2, IL18, and nuclear AhR in a representative normal lung tissue section. Scale bars, 2.5 mm (low magnification) and 100 μm (high magnification).IHC staining of TMPRSS2, IL18, and nuclear AhR in lung cancer tissues that displayed high or low expression of TMPRSS2. Scale bars, 2.5 mm (low magnification) and 100 μm (high magnification).A schematic representation summarizing the mechanism by which particulate matter upregulates TMPRSS2 to promote lung cancer progression. Source data are available online for this figure.

**Figure EV5:**
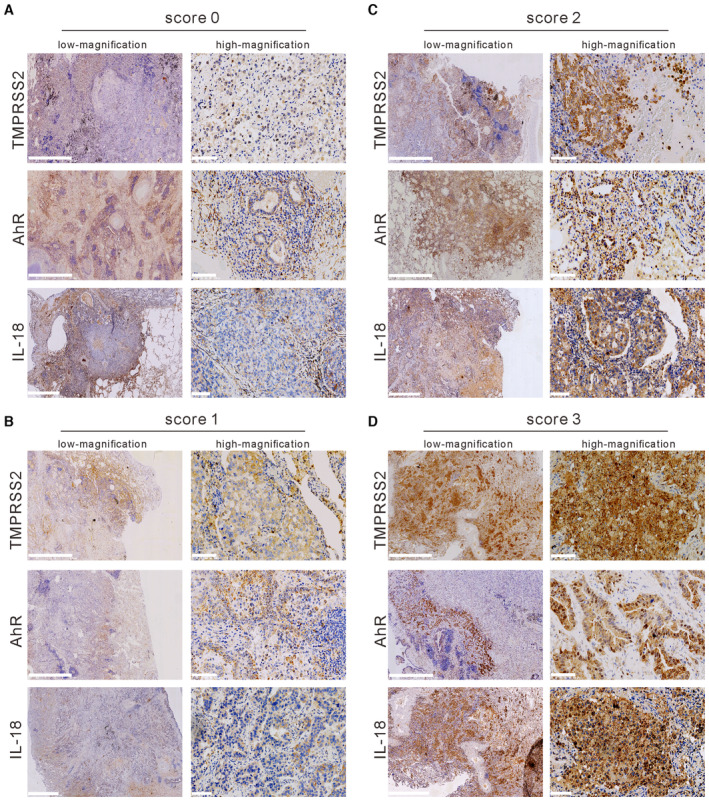
Representative IHC staining images showing immunoreactivity A–D(A) Score 0, (B) score 1, (C) score 2, and (D) score 3 of TMPRSS2, IL18, and AhR in lung cancer tissue. Scale bars, 2.5 mm (low magnification) and 100 μm (high magnification). Source data are available online for this figure.

To evaluate the role of TMPRSS2 expression in lung cancer progression *in vivo*, we examined the expression levels of TMPRSS2, IL18, and nuclear AhR by IHC in tumor specimens from 25 lung cancer patients. Examples of scoring the intensity of immunoactivity in IHC are shown in Fig EV5. Representative immunostaining of normal tissue revealed almost no to very weak expression of TMPRSS2, IL18, and AhR (Fig 7A). IHC staining of two representative tumor specimens revealed that a high expression level of TMPRSS2 was accompanied by a high expression level of nuclear AhR and IL18, while a low expression level of TMPRSS2 was associated with a low expression level of nuclear AhR and IL18 (Fig 7B). Statistical analysis of all specimens showed a significant correlation between the expression level of TMPRSS2 and nuclear AhR (*P* < 0.01) and IL18 (*P* < 0.05) (Table 3). Elevated expression of TMPRSS2 was also positively correlated with the advanced overall stages of lung cancer patients (*P* < 0.05; Table 3). Notably, the expression level of TMPRSS2 also correlated with sex (*P* < 0.05; Table 3).

**Table 3:** Clinical correlation of TMPRSS2 expression with age, sex, nuclear AhR, IL18, and overall stage in 25 lung cancer patients^a^.

Characteristics	TMPRSS2 High^b^ (*N* = 17)	TMPRSS2 Low^c^ (*N* = 8)	*P*‐value
Age (years)			
≤ 60	4	2	1
> 60	13	6	
Gender			
Female	11	1	0.03^d^
Male	6	7	
Nuclear AhR expression			
High^e^	11	0	0.0029^d^
Low^f^	6	8	
IL18 expression			
High^e^	15	3	0.0169^d^
Low^f^	2	5	
Overall stage			
III–IV	10	1	0.04^d^
I–II	7	7	

^a^ By Fisher's test.

^b^ High: Representative lung adenocarcinoma with intense TMPRSS2 immunoreactivity (+: score 2,3).

^c^ Low: Representative lung adenocarcinoma showing no or weak TMPRSS2 immunoreactivity (−: scores 0, 1).

^d^ *P* < 0.05.

^e^ High: Representative lung adenocarcinoma with intense nuclear AhR or IL18 immunoreactivity (+: scores 2, 3).

^f^ Low: Representative lung adenocarcinoma showing no or weak nuclear AhR or IL18 immunoreactivity (−: scores 0, 1).

## Discussion

Most studies of air pollution on the progression of lung cancer are based on “population‐based” epidemiology. In this study, lung cancer cells exposed to PM2.5 for 90 days were employed as a cellular model to evaluate the effects of long‐term exposure to PM2.5 on lung cancer progression. As exposure to PM2.5 can cause EGFR activation (Romagnolo *et al*, 2010; Chen *et al*, 2020), we first examined the effects of exposure to PM2.5 on EGFR signaling. Our results revealed that short‐term exposure to PM2.5 for 24 h indeed activated the EGFR pathway in lung cancer cells (Fig 1). Long‐term exposure to PM 2.5 also upregulated the expression of activated EGFR (pEGFR) in lung cancer cells harboring EGFR mutations (H1975) (Fig 2C). Long‐term exposure to PM2.5 enhanced the ability of H1975 cells to undergo anchorage‐independent growth and to promote tumor cell growth both *in vitro* and *in vivo* (Figs 2 and 3). Therefore, activation of EGFR signaling can be one of the factors that contribute to the enhanced ability to promote tumor growth in PM2.5‐exposed lung cancer cells.

To further explore the mechanism involved in the enhanced ability to promote tumor growth, we conducted whole‐transcriptome sequencing analysis in the H1975 cells exposed to PM2.5 for 90 days. A total of 292 genes exhibited differential expression with twofold increases or decreases in the PM2.5‐treated H1975 cells. Among the 10 differentially expressed genes with the highest scores, TMPRSS2 is a known oncogene that promotes tumor progression and metastasis in prostate cancer (Stone, 2017; Ko *et al*, 2020) and was recently shown to be activated by PM2.5 exposure (Li *et al*, 2021). Therefore, we examined whether TMPRSS2 may be involved in the enhanced ability to promote tumor growth in lung cancer cells. We confirmed that the expression of TMPRSS2 mRNA and protein was increased in the PM2.5‐treated H1975 cells (Fig 4A and B). As PM2.5 is known to activate the translocation of AhR into the nucleus, the increased expression of TMPRSS2 may be attributed to the binding of activated AhR to the TMPRSS2 promoter. Indeed, we confirmed that the level of AhR was increased in the nucleus of the PM2.5‐treated cells (Fig 4C). Furthermore, we showed that the binding of AhR to the TMPRSS2 promoter was enhanced in the PM2.5‐treated cells (Fig 4D), suggesting that the upregulation of TMPRSS2 expression is correlated with the enhanced binding of AhR to the promoter of TMPRSS2.

To delineate the role of TMPRSS2 in promoting tumor growth, we examined the effects of TMPRSS2 depletion on tumor growth. As shown in Fig 5, stable depletion of TMPRSS2 in H1975 cells reduced the ability to undergo anchorage‐independent growth *in vitro* and slowed tumor growth *in vivo*. Therefore, TMPRSS2‐mediated signaling is clearly involved in promoting tumor growth in lung cancers. To systematically explore the effect of TMPRSS2 on lung cancer cells, we used whole‐transcriptome analysis to analyze the effects of TMPRSS2 overexpression on gene expression. As shown in Fig EV4, we identified several pathways that were highly enriched in the TMPRSS2‐overexpressing H1299 cells, including those connected with immune, cell growth, and proliferation signatures. The immune‐associated pathways included coronavirus disease–COVID‐19, legionellosis, influenza A, *Yersinia* infection, malaria, NOD‐like receptor signaling, and pathogenic *Escherichia coli* infection. The pathways associated with cell growth and proliferation included ribosome and hedgehog signaling. Interestingly, transcriptome sequencing analysis of the PM2.5‐treated cells also revealed that PM2.5 affected immune and cell growth and proliferation signatures (Fig EV2). The immune‐associated pathways included viral protein interactions with cytokines and cytokine receptors and cytokine–cytokine receptor interactions. The pathways associated with cell growth and proliferation included transcriptional dysregulation in cancer and the hedgehog signaling pathway (Fig EV2). This finding indicates that upregulated TMPRSS2 expression was positively correlated with cell proliferation and immune modulation in lung cancer progression.

COVID‐19, which is a global pandemic caused by the infection of the new coronavirus SARS‐CoV‐2, has a high diagnosis rate in cities with severe air pollution, and the patient's condition is more complicated and serious. PM is associated with the incidence and severity of COVID‐19 (Yao *et al*, 2020; Zhu *et al*, 2020). SARS‐CoV‐2 enters the host cell by binding the spike (S) protein on the surface of the virus to the receptor angiotensin‐converting enzyme 2 (ACE2) on the surface of the host cell, and then cleaving the S protein by another receptor protein TMPRSS2 on the surface of the host cell to make SARS‐CoV‐2 fuse into the host cell membrane, allowing the virus to enter (Hoffmann *et al*, 2020). Exposure to PM 2.5 promoted the upregulation of TMPRSS2 and ACE2 in lung fibroblasts, which in turn increased the infectivity of SARS‐CoV‐2 and the severity of COVID‐19 (Li *et al*, 2021). Our study shows that PM2.5 induces TMPRESS2 expression, which may make people more susceptible to infection with SARS‐CoV‐2. Therefore, TMPRSS2 appears to play an important role in PM 2.5 causing the more severe effects of COVID‐19 and lung cancer.

IL18 was identified as one of the top 10 upregulated genes in the TMPRSS2‐overexpressing H1299 cells (Table 2). We confirmed that the expression of IL18 is highly correlated with the expression of TMPRSS2 (Fig 6 and Table 3). The secretion of IL18 is known to be controlled by inflammasomes, which are composed of sensor proteins. The first family of sensor proteins revealed to form inflammasomes was NOD‐like receptors (Karki *et al*, 2017). We found that NOD‐like receptor signaling is highly enriched in the TMPRSS2‐overexpressing H1299 cells (Fig EV4). Therefore, these data may indicate that the increased expression of TMPRSS2 in lung cancer cells may affect the formation of inflammasomes to enhance the secretion of IL18. The increased secretion of IL18 may in turn promote cell proliferation, proinflammation, and cancer progression. A recent study indicated that mice with EGFR mutations are more likely to develop cancer after exposure to pollutant particles mediated by an inflammatory protein called interleukin 1 beta, which is part of the body's immune response to PM2.5 exposure (Swanton *et al*, 2022). The role of PM2.5‐induced inflammatory response in cancer progression should be further explored.

Finally, the role of TMPRSS2 expression in lung cancer was examined in tumor specimens from patients. A positive correlation was observed between high levels of TMPRSS2 and nuclear AhR (Table 3), confirming that AhR is associated with the expression of TMPRSS2 *in vivo*. Increased TMPRSS2 expression was also positively correlated with the expression of IL18 (Table 3). Interestingly, increased TMPRSS2 expression was positively correlated with advanced stages of lung cancer, supporting our finding that TMPRSS2 plays an important role in tumor progression of lung cancer. Surprisingly, we observed that high expression of TMPRSS2 was found preferentially in female patients (Table 3). At present, we cannot offer any explanation for this finding.

In conclusion, we have shown that long‐term exposure to PM2.5 promotes tumor progression in lung cancer. Based on the results presented in this study, we propose a schematic model (Fig 7C) in which the mechanism involved in promoting tumor progression by exposure to PM2.5 may include (i) activation of EGFR signaling and (ii) activation of AhR to upregulate TMPRSS2, which in turn upregulates its downstream targets, such as IL18.

## Materials and Methods

### Reagents and Tools table

**Table:** 

Antibody sources	Reference or source	Identifier or catalog number
**Antibidy for Western blot and IHC**		
Rabbit anti‐pEGFR	Cell Signaling	#2234
Rabbit anti‐EGFR	Cell Signaling	#4267
Rabbit anti‐pSTAT3	Cell Signaling	#9145
Rabbit anti‐STAT3	Cell Signaling	#4904
Rabbit anti‐pAKT	Cell Signaling	#9271
Rabbit anti‐AKT	Santa Cruz	sc‐8312
Rabbit anti‐pERK	Cell Signaling	#9101
Rabbit anti‐ERK	Santa Cruz	sc‐94
Rabbit anti‐Ki‐67	Cell Signaling	#9027
Mouse anti‐HA	Sigma	H9658
Mouse anti‐TMPRSS2	Santa Cruz	sc‐515727
Rabbit anti‐IL‐18	Cell Signaling	#67775
Rabbit anti‐Vimentin	Cell Signaling	#5741
Rabbti anti‐AhR	GeneTex	GTX129012
Goat anti‐Lamin B	Santa Cruz	sc‐6216
Mouse anti‐β‐actin	Sigma	A5441
**Antibidy for IP, IHC and IF**		
Mouse anti‐AhR	Santa Cruz	sc‐133088
Goat anti mouse Alexa®Fluor 488	Life Technologies Molecular Probes	A111001
**Chemical reagent**		
CH‐223191	MedChemExpress	HY‐12684
**Sequence‐based reagents**		
**qPCR primers**	**Forward (5′‐3′)**	**Reverse (3′‐5′)**
TMPRSS2	ACTCTGGAAGTTCATGGGCAG	TGAAGTTTGGTCCGTAGAGGC
IL‐18	CTGCCACCTGCTGCAGTCTA	TCTACTGGTTCAGCAGCCATCTTTA
GAPDH	CACTAGGCGCTCACTGTT CTC	GCCCAATACGACCAAATCC

### Methods and Protocols

#### Cell lines and culture

A549, H1975, H460, H1299, IMR90, and MRC5 cells were obtained from the American Type Culture Collection (Manassas, VA, USA). The lung cancer cell line PC9 was kindly provided by Dr. Chih‐Hsin Yang (National Taiwan University). All of the cells were cultured in RPMI‐1640 medium. The short tandem repeat assay was used to confirm the identity of all cell lines. The absence of mycoplasma contamination was tested by a polymerase chain reaction (PCR)‐based enzyme‐linked immunosorbent assay (ELISA; Sigma).

#### Source and preparation of PM2.5

The source of PM2.5 used in this work is the National Institute of Standards and Technology (NIST, USA) particulate matter SRM 1650b and was obtained from Sigma‐Aldrich (St. Louis, MO, USA). The major components of this PM2.5 are PAHs and nitro‐PAHs. PM2.5 was dissolved in DMSO at 25 mg/ml, sonicated for 30 min, and used within 1 h for the experiments performed (Piao *et al*, 2018). The PM2.5 was diluted 100‐fold with double‐distilled water, and the average diameter was measured by a laser‐scattering method (Nano ZS90, Malvern). The diameter of the prepared PM 2.5 measured was 992 ± 157.2 nm.

#### Reagents

Culture media and chemical compounds were purchased from Life Technologies (Grand Island, NY, USA). Antibodies against phosphor (P)‐EGFR (1:1,000), EGFR (1:1,000), pSTAT3 (1:1,000), STAT3 (1:1,000), pAKT (1:1,000), pERK (1:1,000), IL‐18 (1:200), vimentin (1:1,000), and Ki‐67 (1:1,000) were purchased from Cell Signaling (Temecula, CA, USA). Anti‐AKT (1:1,000), anti‐ERK (1:1,000), anti‐TMPRSS2 (1:500), anti‐AhR (1:500), and anti‐Lamin B (1:1,000) were obtained from Santa Cruz Biotechnology (Santa Cruz, CA, USA). The antibody against AhR (1:500) was purchased from GeneTex (Irvine, CA, USA). The antibody against β‐actin (1:1,000) and HA (1:1,000) was purchased from Sigma (St. Louis, MO, USA). Alexa Fluor® 488 goat anti‐mouse IgG (1:1,000) was purchased from Life Technologies (Waltham, MA, USA). CH223191 was purchased from Sigma (St. Louis, MO, USA).

#### Plasmids and establishment of stable knockdown subclones

Full‐length TMPRSS2 with HA tagged at the C‐terminus was cloned into the pcDNA3.1 vector using NotI and XbaI restriction enzymes (TMPRSS2‐HA). Plasmid DNAs were transfected into cells using Lipofectamine 2000 (Invitrogen, Waltham, Massachusetts, USA) according to the manufacturer's protocol. Packed lentiviruses expressing short hairpin RNA (shRNAs) designed to knock down the expression of TMPRSS2 (TRCN0000000265 and TRCN0000000266) were obtained from the RNA interference consortium (National RNAi Core Facility, Academia Sinica, Taipei, Taiwan). The stable knockdown H1975 cells were designated shTMPRSS2‐1 and shTMPRSS2‐2, while the control was named the vector (control).

#### Whole‐transcriptome sequencing and functional enrichment analysis

Total RNA was extracted from H1975/H1975‐PM 2.5 cells treated for 90 days and H1299/H1299 cells overexpressing TMPRSS2 using TRIzol^®^ Reagent (Invitrogen, USA). Whole‐transcriptome sequencing and functional enrichment analysis were performed as described in a previous study (Leu *et al*, 2020). The RNA‐sequencing data generated in this study have been deposited in the Gene Expression Omnibus under the accession GSE220252 and GSE220306.

#### Assays for cell viability and anchorage‐independent growth

Viable cell counts by trypan blue exclusion assays and anchorage‐independent growth by soft agar colony formation assays were performed as described previously (Chen *et al*, 2009, 2016).

#### Chromatin immunoprecipitation–qPCR

Chromatin immunoprecipitation assays were performed using an Immunoprecipitation Assay Kit (Merck Millipore, Temecula, CA) according to the manufacturer's instructions. Each of the purified DNAs (10 pg) was used as a template for qPCR via five primers across potential TMPRSS2 regulatory regions (Leach *et al*, 2021): RG1a: (F)CCTTGCAATTGCTGACCCCA, (R)ACAGCAAGATGGCTTTGAACT; RG1d: (F)CATGAGGGCAGTGAGAGTGC, (R)TTTCTCTGGTCCCAGCCATC; RG1f: (F)TTACACCACTGGCTATTGGCTC, (R)CTTTTCAGCCTTGGACATCGG; RG1h: (F)CCAAAAGTGTTGCTCGGCTT, (R)AGAAGTGCAGCTGGCATCG; RG2a: (F)GCAACCTGAGCCTGTTGACT, (R)CTCAGGTCAGGCTTCCACAC.

#### RT–PCR, Western blotting, and immunofluorescence staining

The cell lysate for analysis by RT–PCR and Western blotting was performed as described previously (Chen *et al*, 2013; Wang *et al*, 2017). The immunofluorescence staining of cells and tumor sections was performed as described previously (Wang *et al*, 2017).

#### *In vivo* tumor xenograft study

The detailed procedure for tumor xenografts study was performed as previously described (Chen *et al*, 2018). Six‐week‐old nude BALB/c nu/nu male mice (the National Laboratory Animal Center, Taipei, Taiwan) were used in this work and the experiments were performed in accordance with the guidelines for the Animal Care Ethics Commission of Chang Gung Memorial Hospital (IACUC approval number: 2019032009 and 2020121704) and Chang Gung University (IACUC approval number: CGU110‐100) and under an approved animal protocol. All mice were housed under specific pathogen‐free conditions and maintained at 23 ± 1°C with a 12 h dark/light cycle. All mice had free access to standard autoclaved food and water for the duration of the study.

#### Tumor specimens

Tumor and corresponding noncancerous normal tissues were collected from 25 lung cancer patients who underwent surgical resection at Chang Gung Memorial Hospital (Tao‐Yuan, Taiwan, ROC) between 2013 and 2017. This study was reviewed and approved by the Institutional Review Board and Ethics Committee of Chang Gung Memorial Hospital (IRB No.: 202102073B0C501). Written informed consent was obtained from all patients. The experiments conformed to the principles set out in the WMA Declaration of Helsinki and the Department of Health and Human Services Belmont Report.

#### Immunohistochemistry (IHC)

Immunohistochemistry was performed as described previously (Jan *et al*, 2011). The scoring of IHC staining results was performed by two expert pathologists blinded to the clinical details. Immunoreactivity was classified into four classes: scored 0 for undetectable staining, scored 1 for weak staining, scored 2 for low intensity, and scored 3 for high intensity.

#### Statistical analysis

The presented results were representative of three independent experiments with similar results. Data in this study all followed normal distribution tested by Shapiro–Wilk test (*P* > 0.05). One‐sample *t*‐test was used to test whether relative phosphor protein expression, relative RNA expression, relative protein expression, relative proliferation of experiment group, and relative colony formation ability under null hypotheses mean equal to 1. To compare differences in proliferation, tumor weight, and tumor volume on the last observation day between treated group and control group, the variance equality between the two groups was examined by F‐test, and a further two‐sample *t*‐test with or without equal variance was applied. For the comparison of fold change of AhR binding to different TMPRSS2 promoter regions, the Levene's test was used first for evaluating homogeneity of variance across promoter regions. If *P*‐value of Levene's test > 0.05, the one‐way analysis of variance (ANOVA) with *post hoc* analysis (Tukey's multiple‐comparison test) was applied to compare differences between three groups (RG1a, RG1d, and RG1f). Otherwise, Welch's ANOVA and Games–Howell test of *post hoc* analysis was used (RG1h, RG2a). To assess relative cell ability among different cell lines (A549, IMR90, MRC5, and H1975) and PM2.5 concentration, the two‐way ANOVA and Tukey's multiple‐comparisons test of *post hoc* analysis were used. The two‐way repeated‐measurement ANOVA was used to evaluate treated effects over time on tumor volume, and pairwise comparison of *post hoc* analysis with Benjamini–Hochberg (BH) correction was conducted to the evaluated treated effect of PM2.5 on tumor volume in different time points. If multiple statistical tests were conducted in one experiment, BH method for adjusting multiple hypothesis testing was done. All tests were two tails and *P*‐value < 0.05 was considered statistically significant. The scoring of IHC staining results was done in a blinded fashion by two pathologists.

## Author contributions

**Tong‐Hong Wang:** Data curation; software; visualization; methodology; writing – original draft. **Kuo‐Yen Huang:** Data curation; visualization; methodology; writing – original draft. **Chin‐Chuan Chen:** Data curation; formal analysis. **Ya‐Hsuan Chang:** Formal analysis. **Hsuan‐Yu Chen:** Formal analysis. **Chuen Hsueh:** Data curation; software. **Yi‐Tsen Liu:** Data curation; formal analysis. **Shuenn‐Chen Yang:** Data curation; formal analysis. **Pan‐Chyr Yang:** Conceptualization; data curation; supervision; visualization; writing – review and editing. **Chi‐Yuan Chen:** Supervision; funding acquisition; writing – original draft; project administration; writing – review and editing.

## Disclosure and competing interests statement

The authors declare that they have no conflict of interest.

## Supporting information



Expanded View Figures PDF

Source Data for Expanded View

PDF+

Source Data for Figure 1

Source Data for Figure 2

Source Data for Figure 3

Source Data for Figure 4

Source Data for Figure 5

Source Data for Figure 6

Source Data for Figure 7

## Data Availability

RNA sequencing data generated in this study have been deposited at the Gene Expression Omnibus under the following accession code GSE220252 (https://www.ncbi.nlm.nih.gov/geo/query/acc.cgi?acc=GSE220252) and GSE220306 (https://www.ncbi.nlm.nih.gov/geo/query/acc.cgi?acc=GSE220306).
