# Supplementary material for: PM2.5 promotes lung cancer progression through activation of the AhR‐TMPRSS2‐IL18 pathway
Source: EMBO Mol Med. 2023 Mar 28;15(6):e17014. doi: 10.15252/emmm.202217014 (PMC10245036; doi:10.15252/emmm.202217014)
Supplement: Supplementary file 1 — Expanded View Figures PDF [file EMMM-15-e17014-s008.pdf]

Expanded View Figures

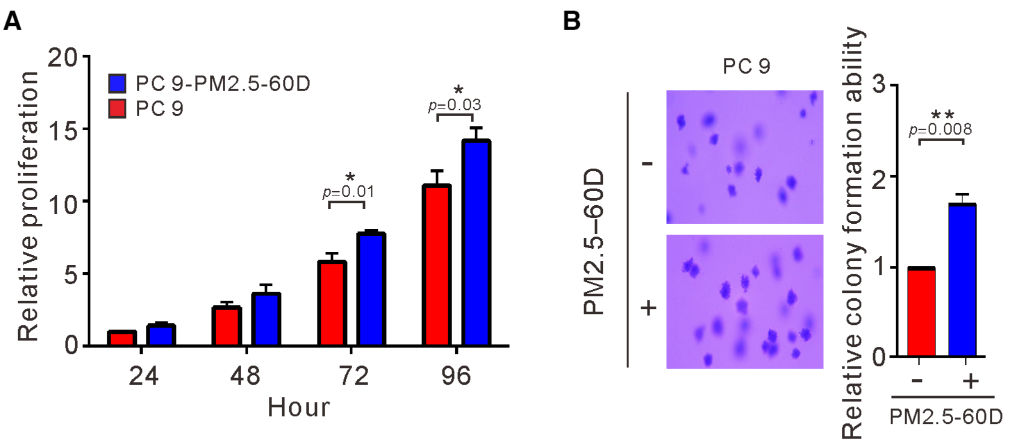

Figure EV1. Effects of long-term exposure to PM2.5 on cell proliferation and anchorage-independent growth of PC9 lung cancer cells.

A, B PC9 cells were treated with PM2.5 at 50 μg/ml for 60 days. The proliferation of treated cells was assessed by Trypan blue assay (A). The anchorage-independent growth was assessed by a soft agar colony formation assay (B). The data shown are the means ± SDs from three independent experiments. *$P < 0.05$ and **$P < 0.01$, compared with untreated cells. (A) *P*-values were determined by two-sample *t*-test. (B) *P*-values were determined by one-sample *t*-test.

Source data are available online for this figure.

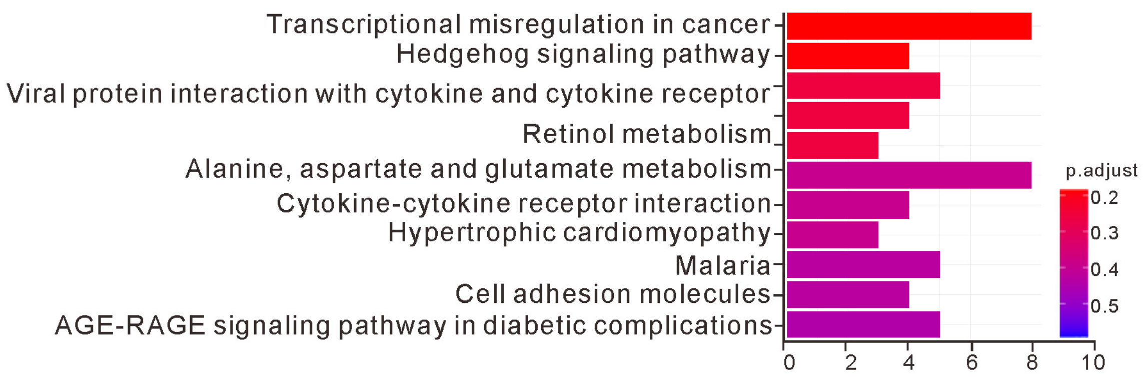

Figure EV2. The top 10 enriched biological processes in long-term exposure to PM2.5.

H1975 cells were exposed to 50 μg/ml PM2.5 for 90 days, and total RNA was subjected to whole-transcriptome analysis as described in the Materials and Methods. Functional classification of the differentially expressed genes in PM2.5-treated H1975 cells, as assessed using ingenuity pathway analysis.

Source data are available online for this figure.

© 2023 The Authors

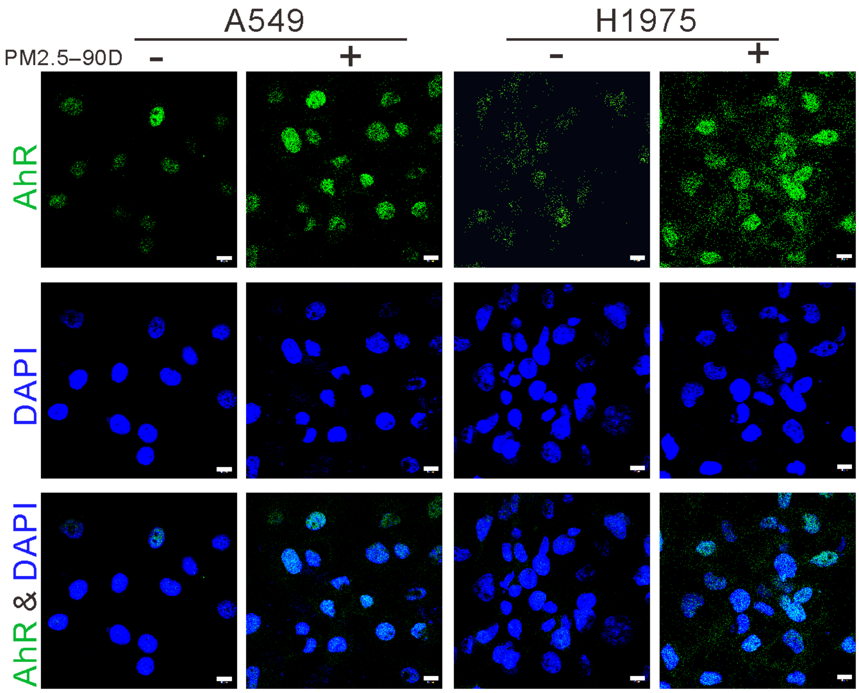

Figure EV3. Immunofluorescence staining of AhR.

The subcellular distribution of AhR was assessed by immunofluorescence staining in H1975 and A549 cells treated with 50 μg/ml PM2.5 for 90 days. DAPI was used as a nuclear stain. Scale bar: 10 μm.

Source data are available online for this figure.

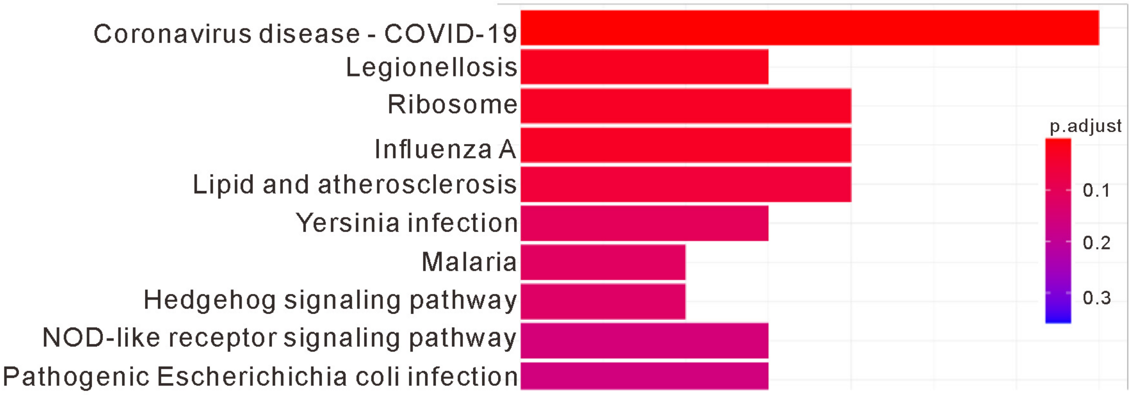

Figure EV4. The top 10 enriched biological processes in TMPRSS2 overexpression.

H1299 cells were transfected with TMPRSS2-HA or the empty vector (Vector). After 48 h, the transfected cells were subjected to whole-transcriptome analysis. Functional classification of the differentially expressed genes in TMPRSS2-overexpressing H1299 cells, as assessed using ingenuity pathway analysis.

Source data are available online for this figure.

© 2023 The Authors

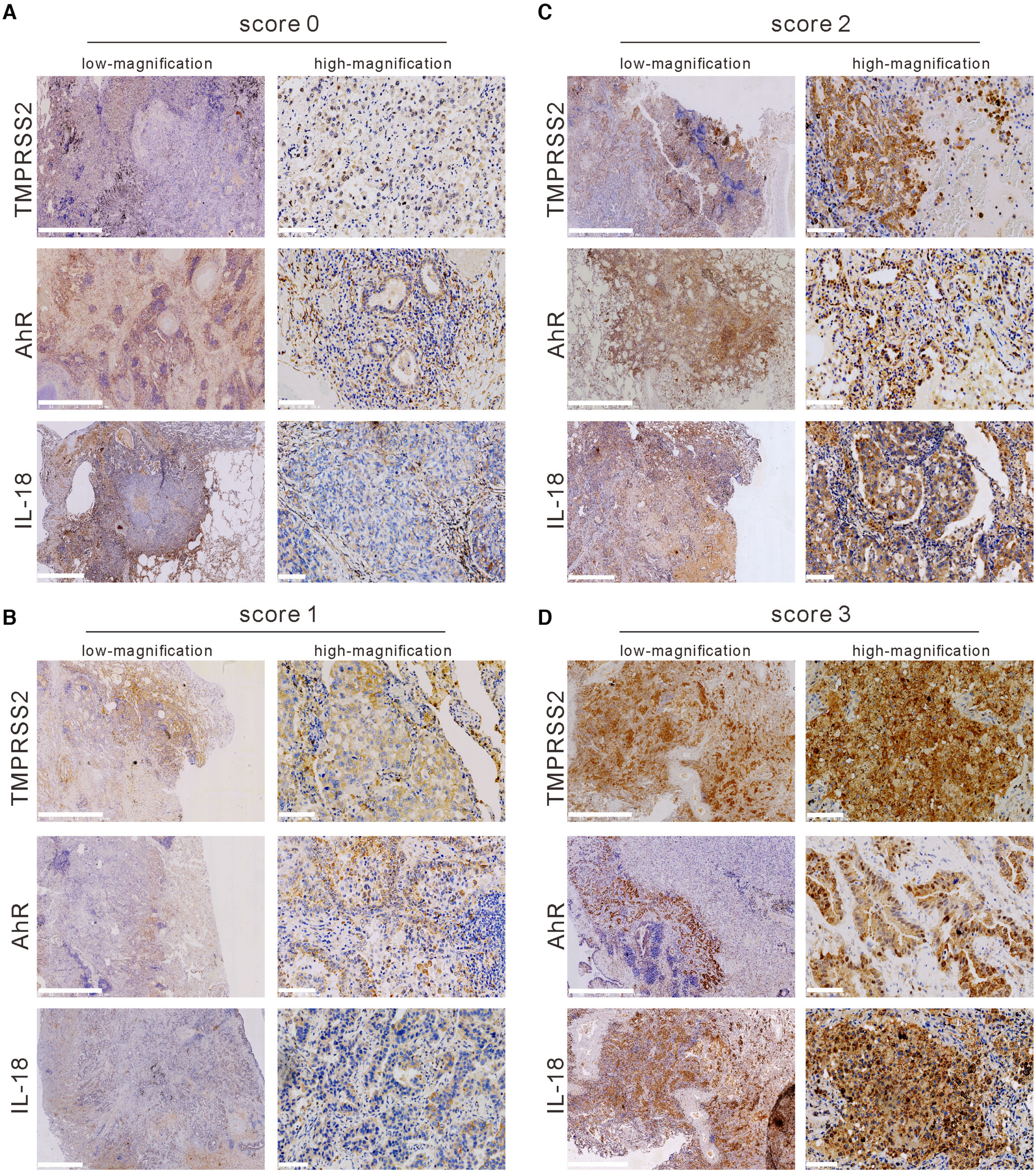

Figure EV5. Representative IHC staining images showing immunoreactivity.

A–D (A) Score 0, (B) score 1, (C) score 2, and (D) score 3 of TMPRSS2, IL18, and AhR in lung cancer tissue. Scale bars, 2.5 mm (low magnification) and 100 μm (high magnification).

Source data are available online for this figure.
