# Supplementary figures and images for: PM2.5 promotes lung cancer progression through activation of the AhR‐TMPRSS2‐IL18 pathway
Source: EMBO Mol Med. 2023 Mar 28;15(6):e17014. doi: 10.15252/emmm.202217014 (PMC10245036; doi:10.15252/emmm.202217014)

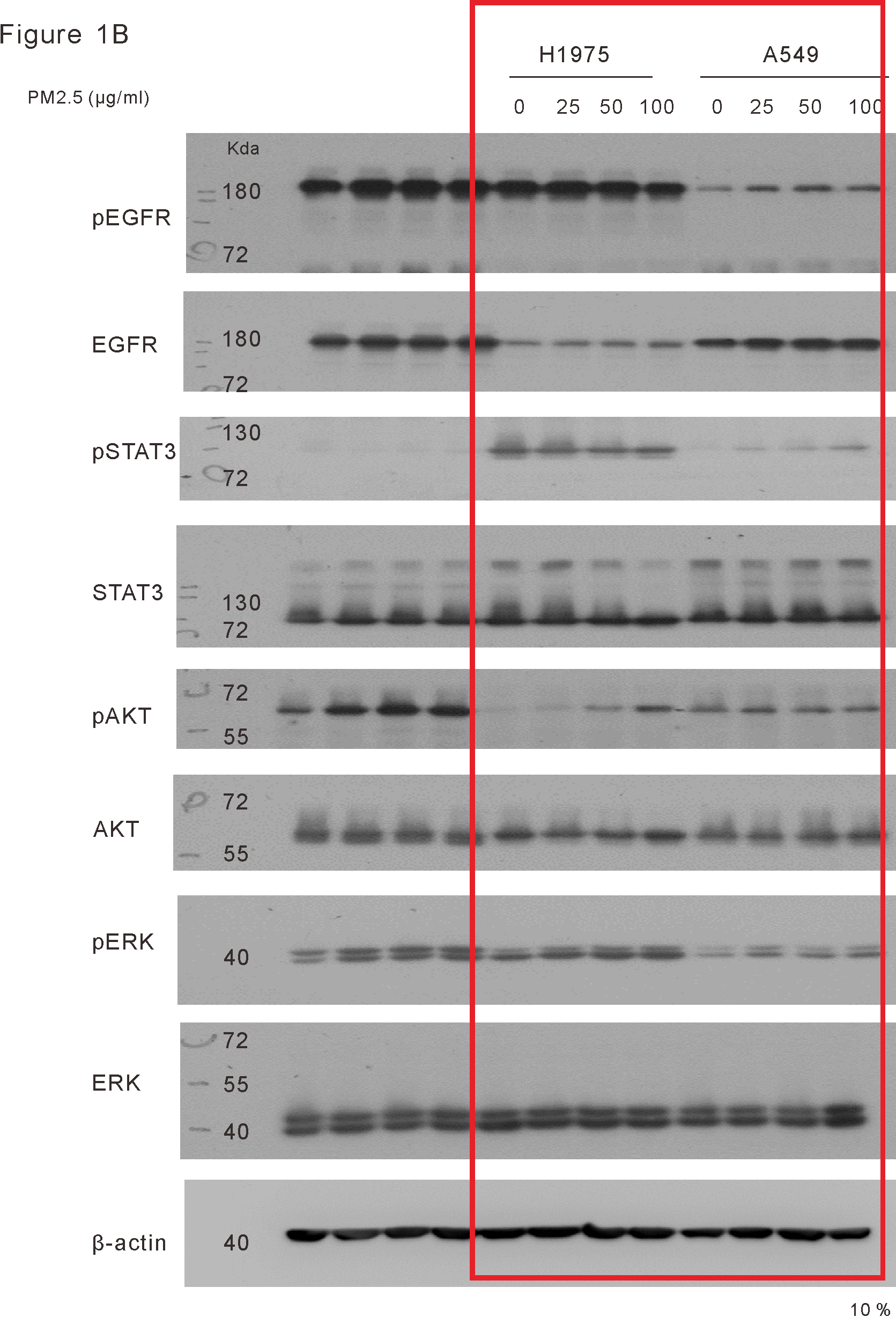

Supplement: Supplementary file 4 — Source Data for Figure 1 [file EMMM-15-e17014-s002.zip › Figure 1/Fig. 1B-Image data.tif]

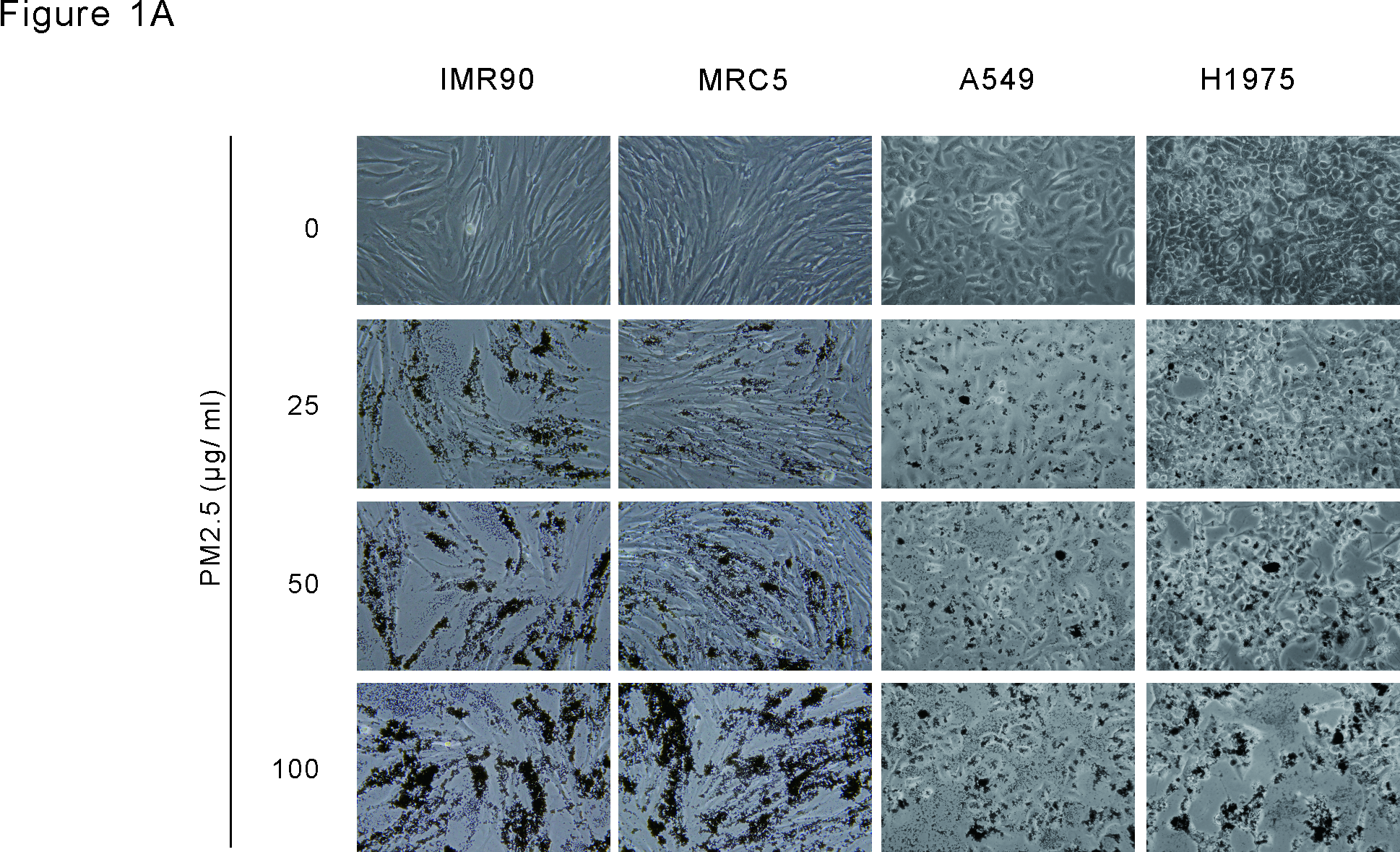

Supplement: Supplementary file 4 — Source Data for Figure 1 [file EMMM-15-e17014-s002.zip › Figure 1/Fig. 1A-Image data.tif]

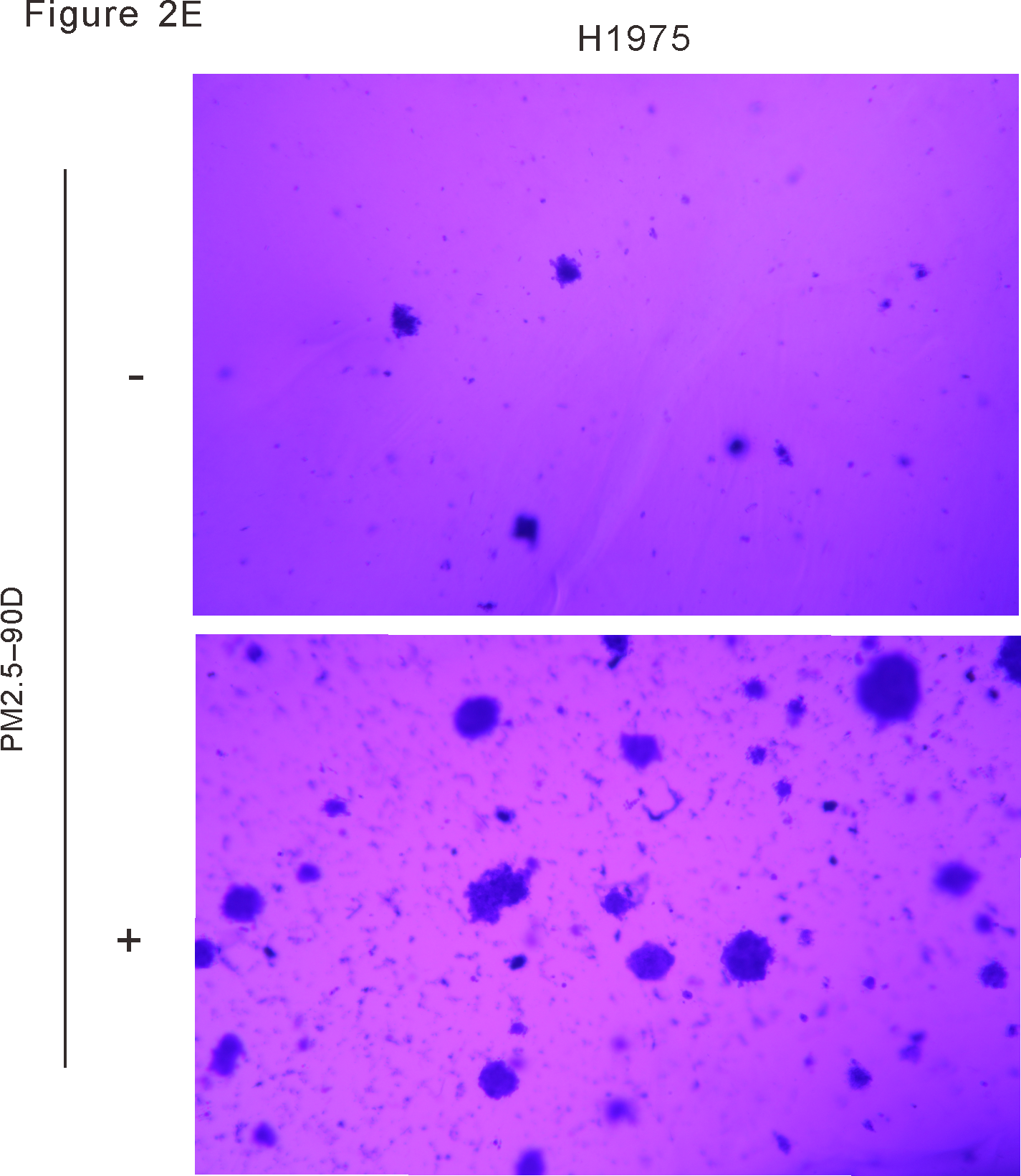

Supplement: Supplementary file 5 — Source Data for Figure 2 [file EMMM-15-e17014-s006.zip › Figure 2/Fig. 2E-Image data.tif]

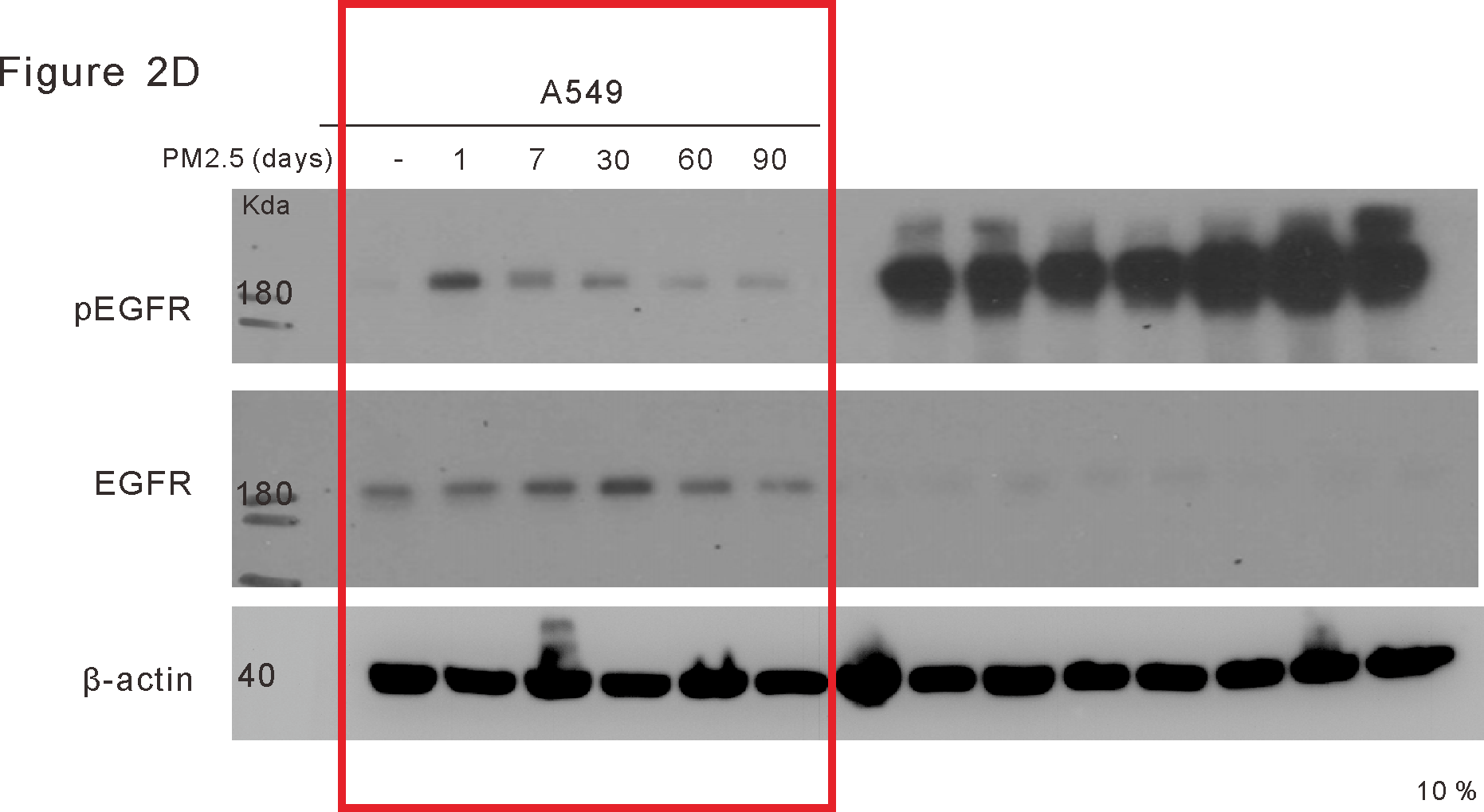

Supplement: Supplementary file 5 — Source Data for Figure 2 [file EMMM-15-e17014-s006.zip › Figure 2/Fig. 2D-Image data.tif]

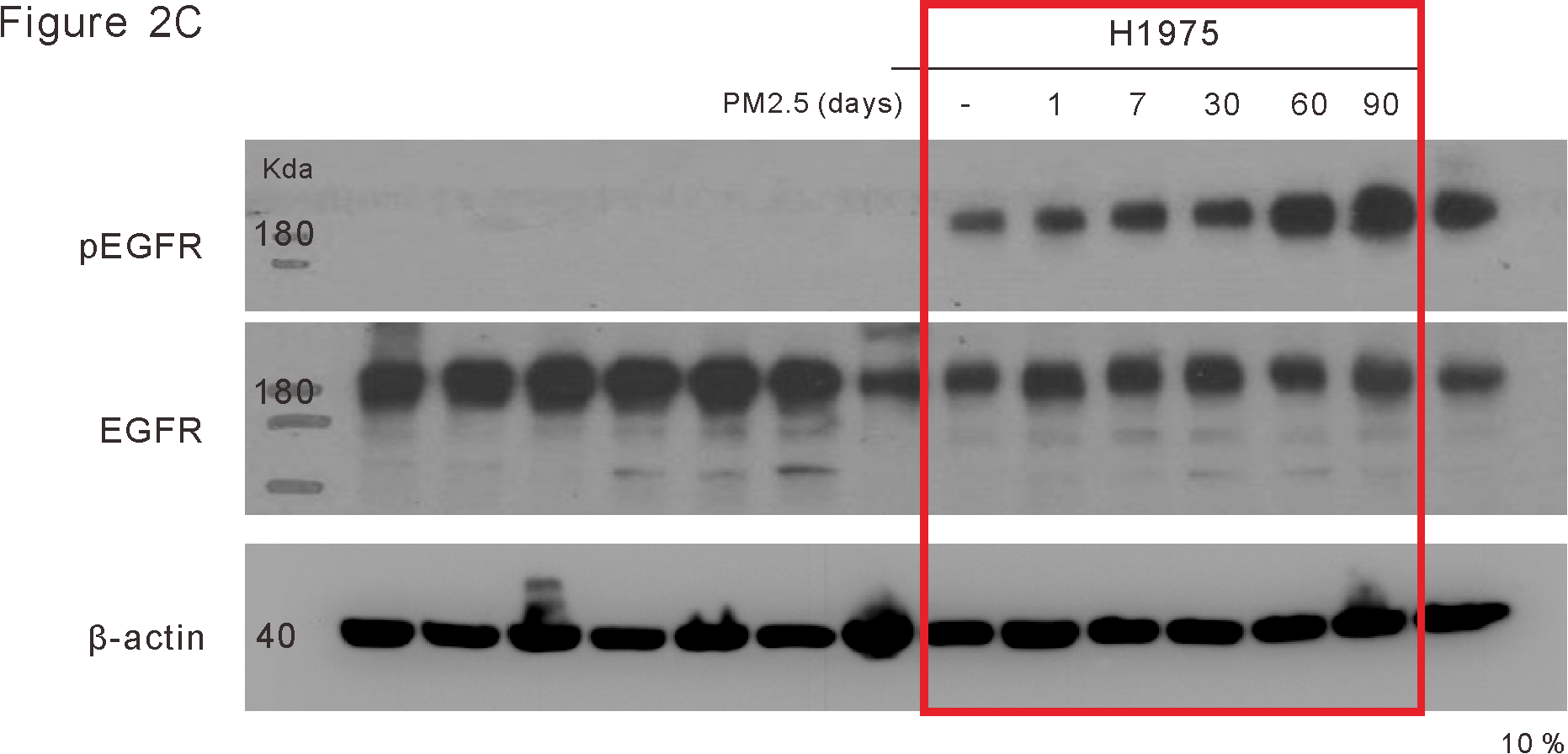

Supplement: Supplementary file 5 — Source Data for Figure 2 [file EMMM-15-e17014-s006.zip › Figure 2/Fig. 2C-Image data.tif]

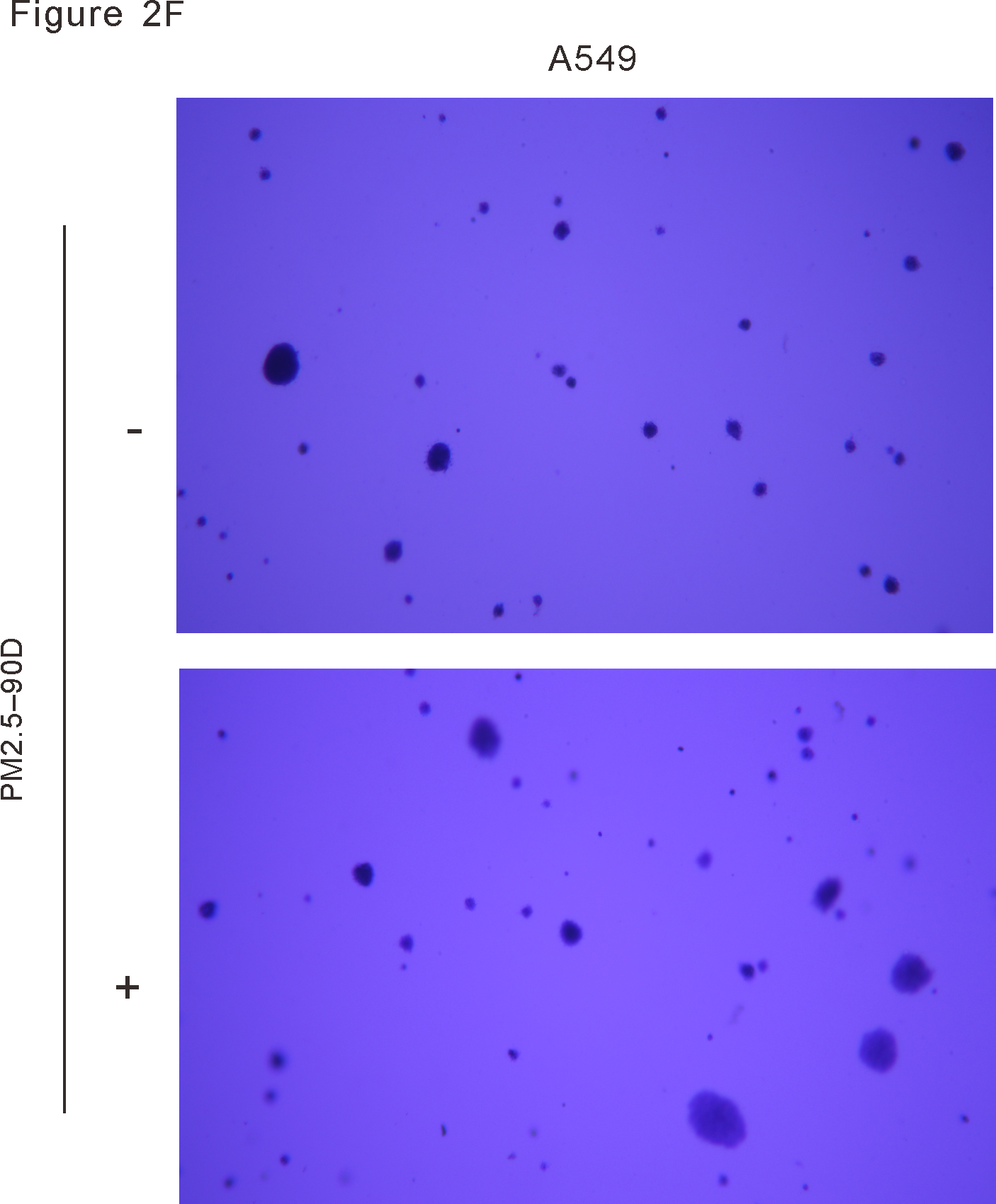

Supplement: Supplementary file 5 — Source Data for Figure 2 [file EMMM-15-e17014-s006.zip › Figure 2/Fig. 2F-Image data.tif]

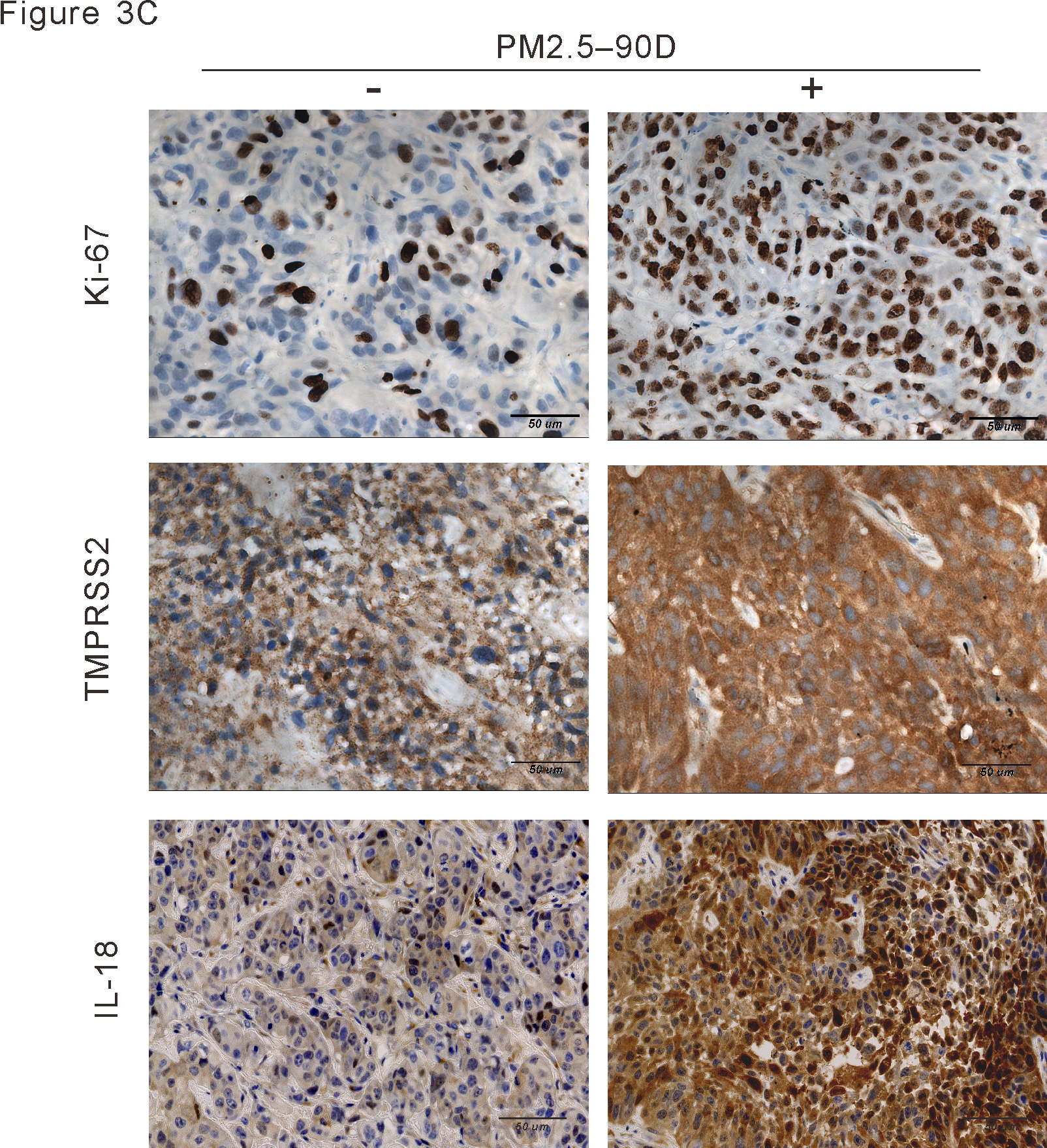

Supplement: Supplementary file 6 — Source Data for Figure 3 [file EMMM-15-e17014-s005.zip › Figure 3/Fig. 3C-Image data.tif]

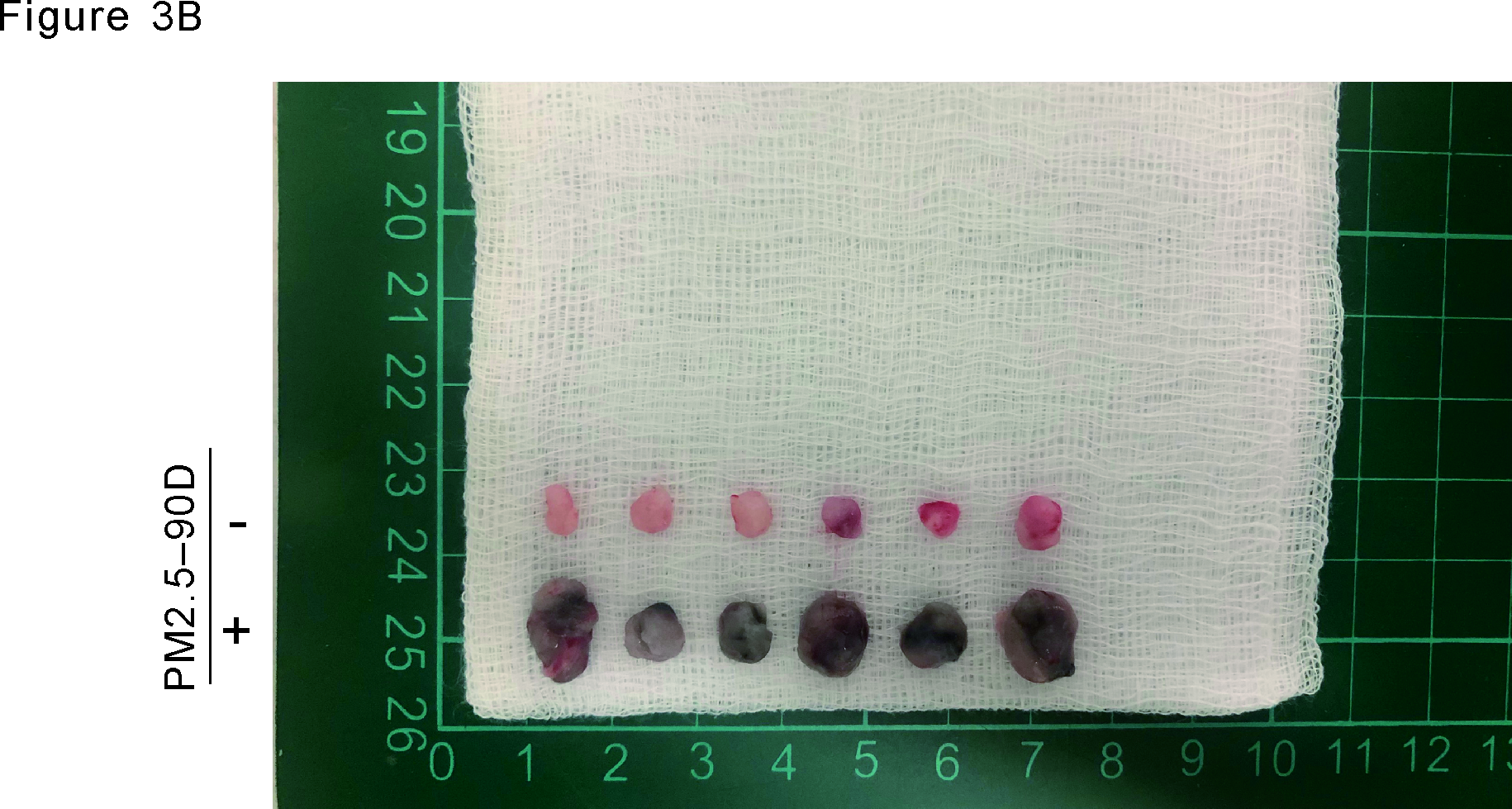

Supplement: Supplementary file 6 — Source Data for Figure 3 [file EMMM-15-e17014-s005.zip › Figure 3/Fig. 3B-Image data.tif]

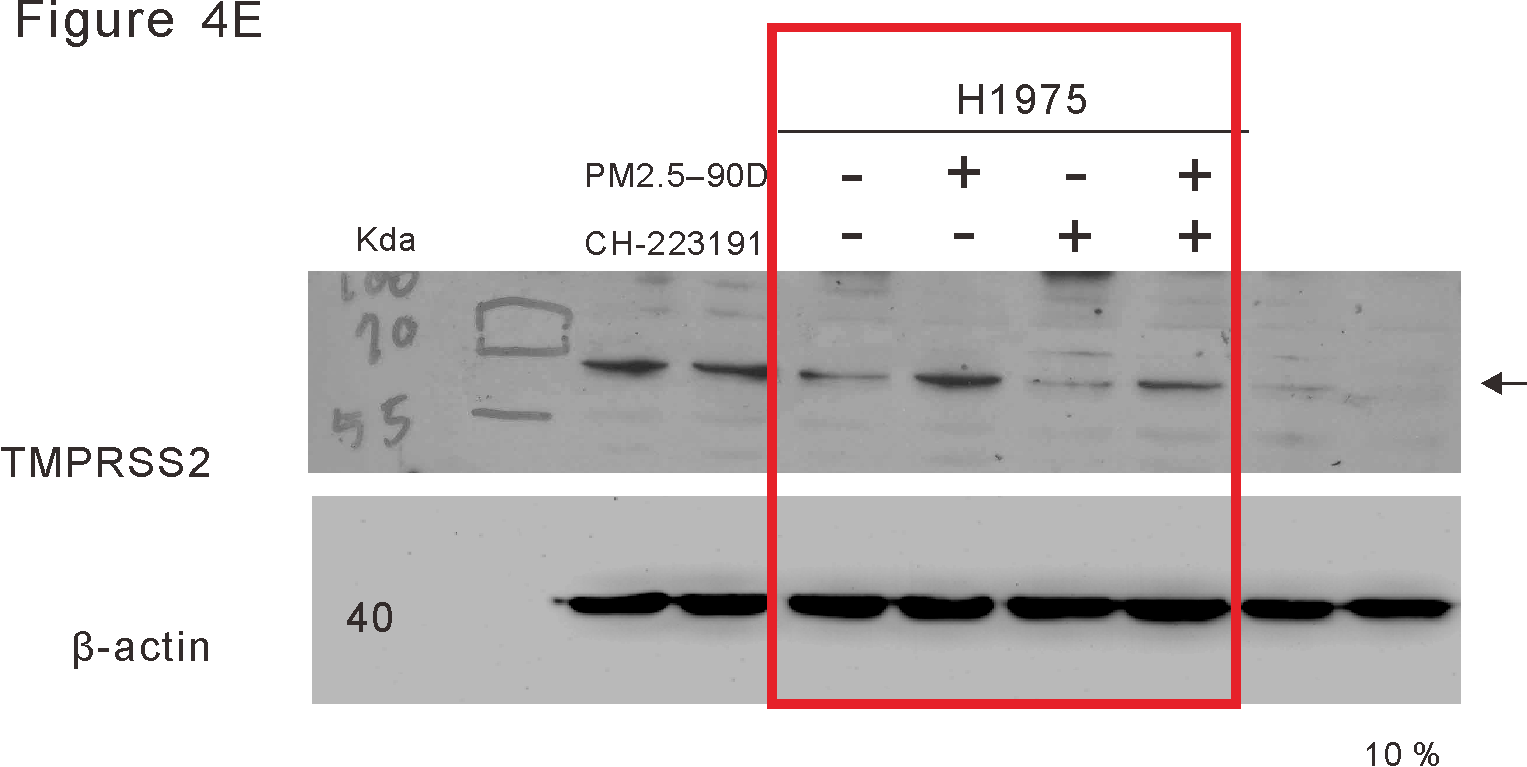

Supplement: Supplementary file 7 — Source Data for Figure 4 [file EMMM-15-e17014-s004.zip › Figure 4/Fig. 4E-Image data.tif]

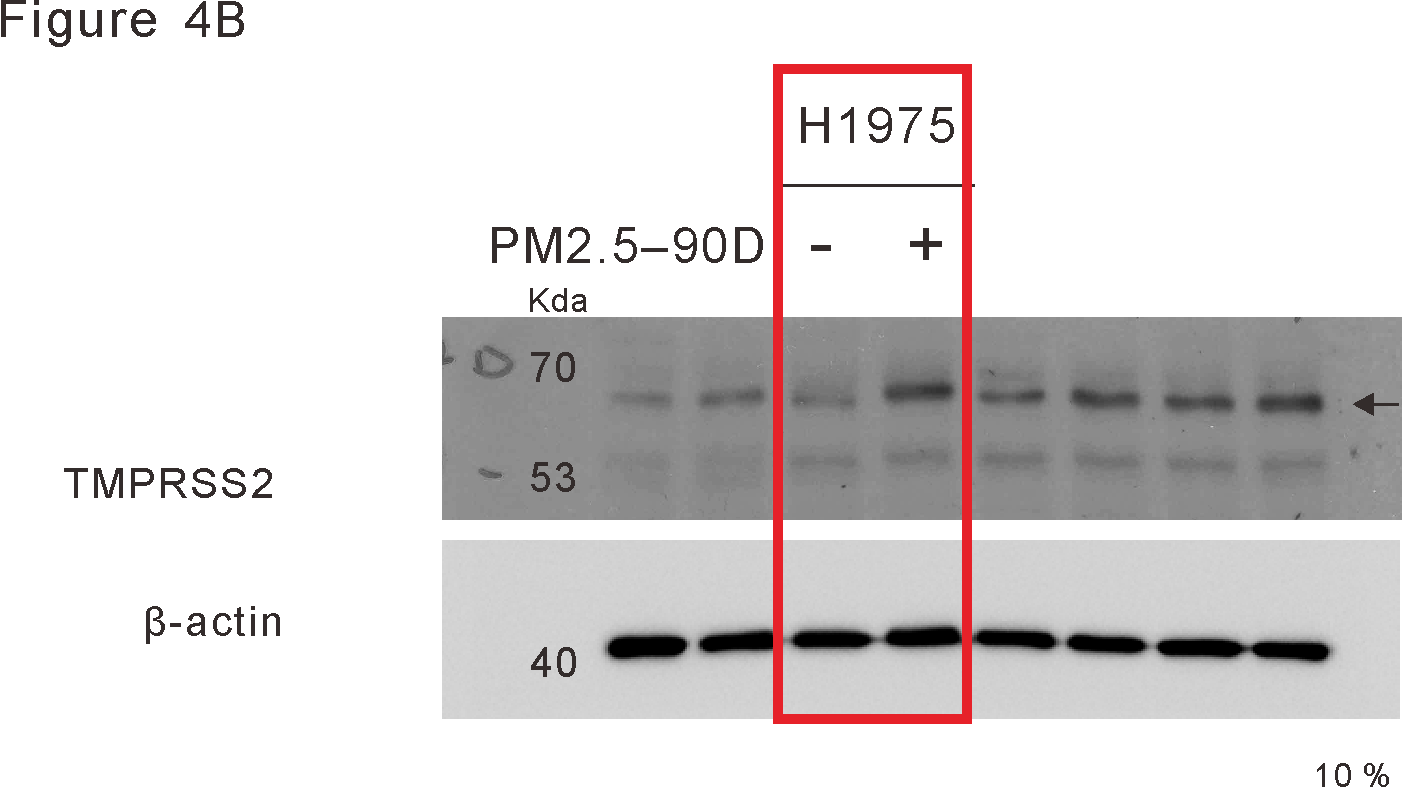

Supplement: Supplementary file 7 — Source Data for Figure 4 [file EMMM-15-e17014-s004.zip › Figure 4/Fig. 4B-Image data.tif]

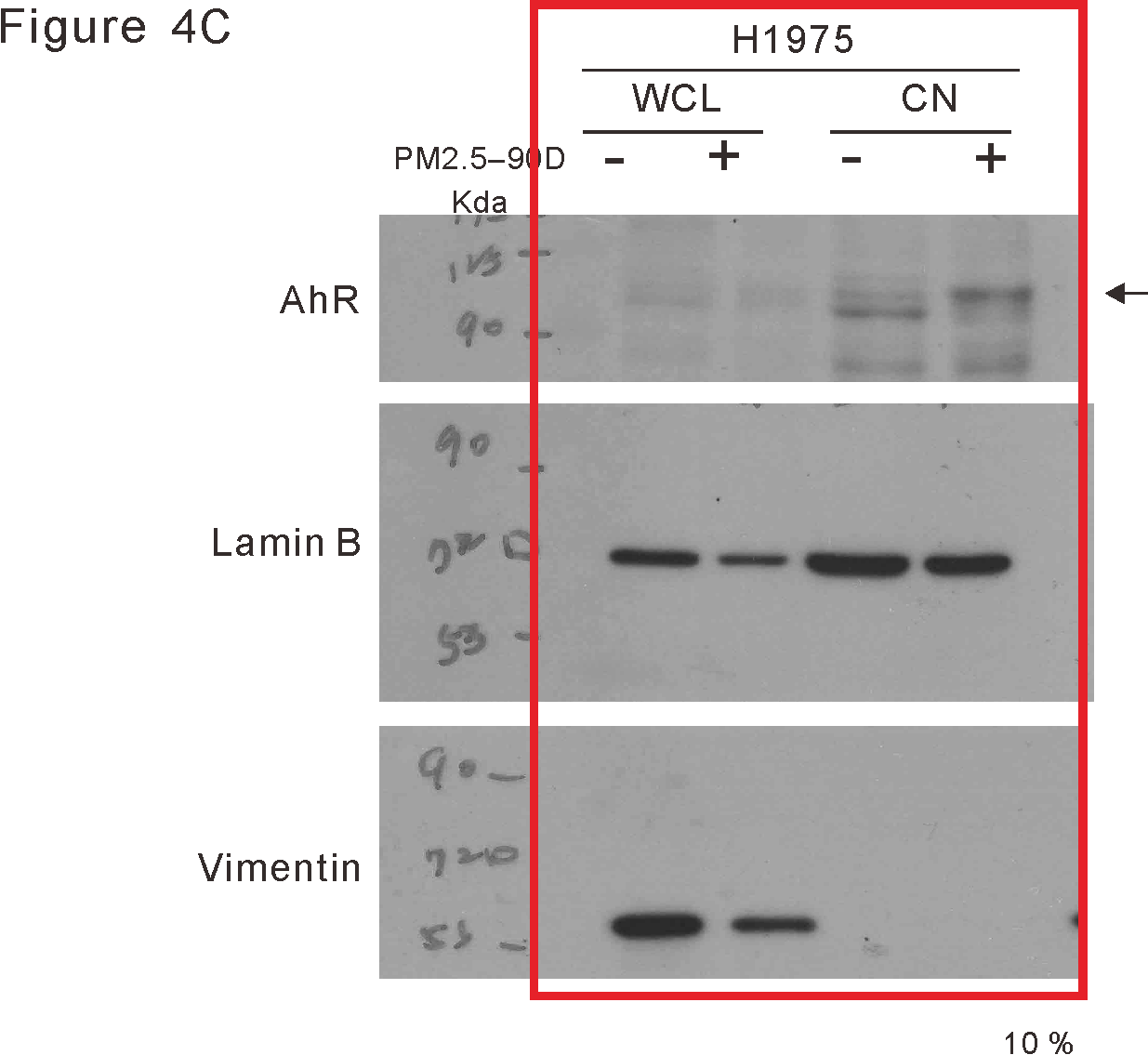

Supplement: Supplementary file 7 — Source Data for Figure 4 [file EMMM-15-e17014-s004.zip › Figure 4/Fig. 4C-Image data.tif]

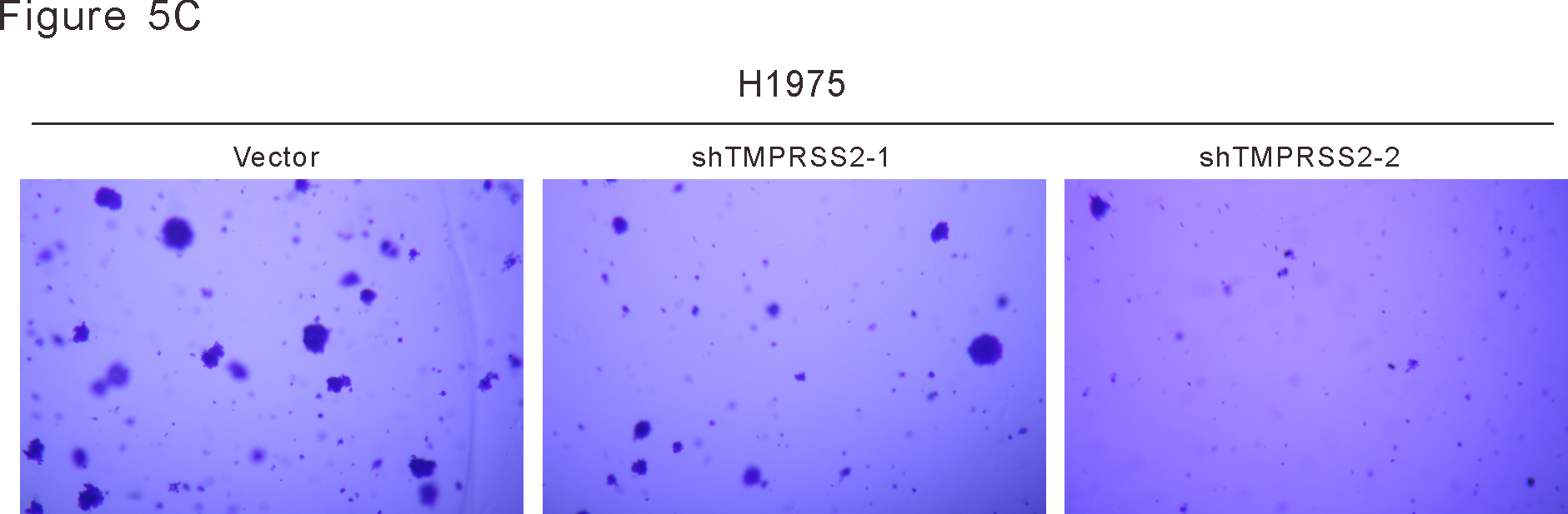

Supplement: Supplementary file 8 — Source Data for Figure 5 [file EMMM-15-e17014-s010.zip › Figure 5/Fig. 5C-Image data.tif]

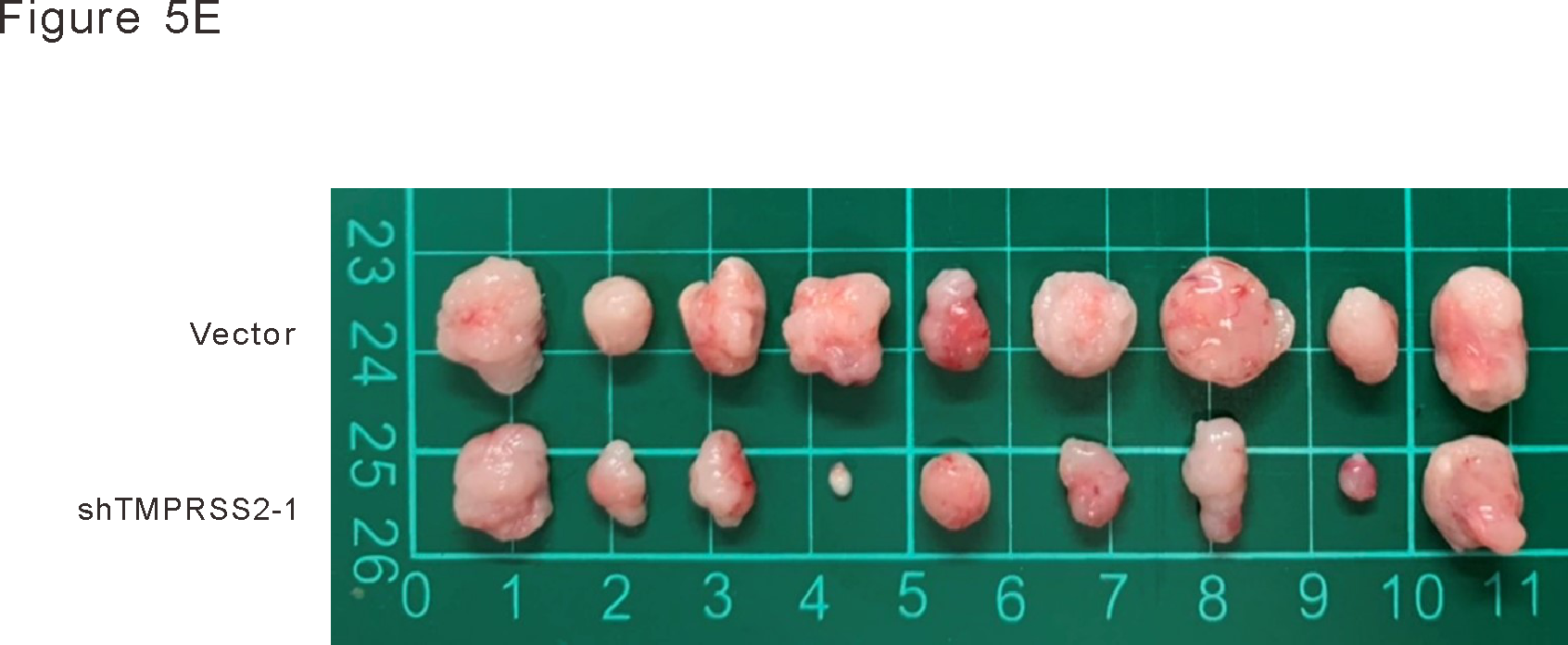

Supplement: Supplementary file 8 — Source Data for Figure 5 [file EMMM-15-e17014-s010.zip › Figure 5/Fig. 5E-Image data.tif]

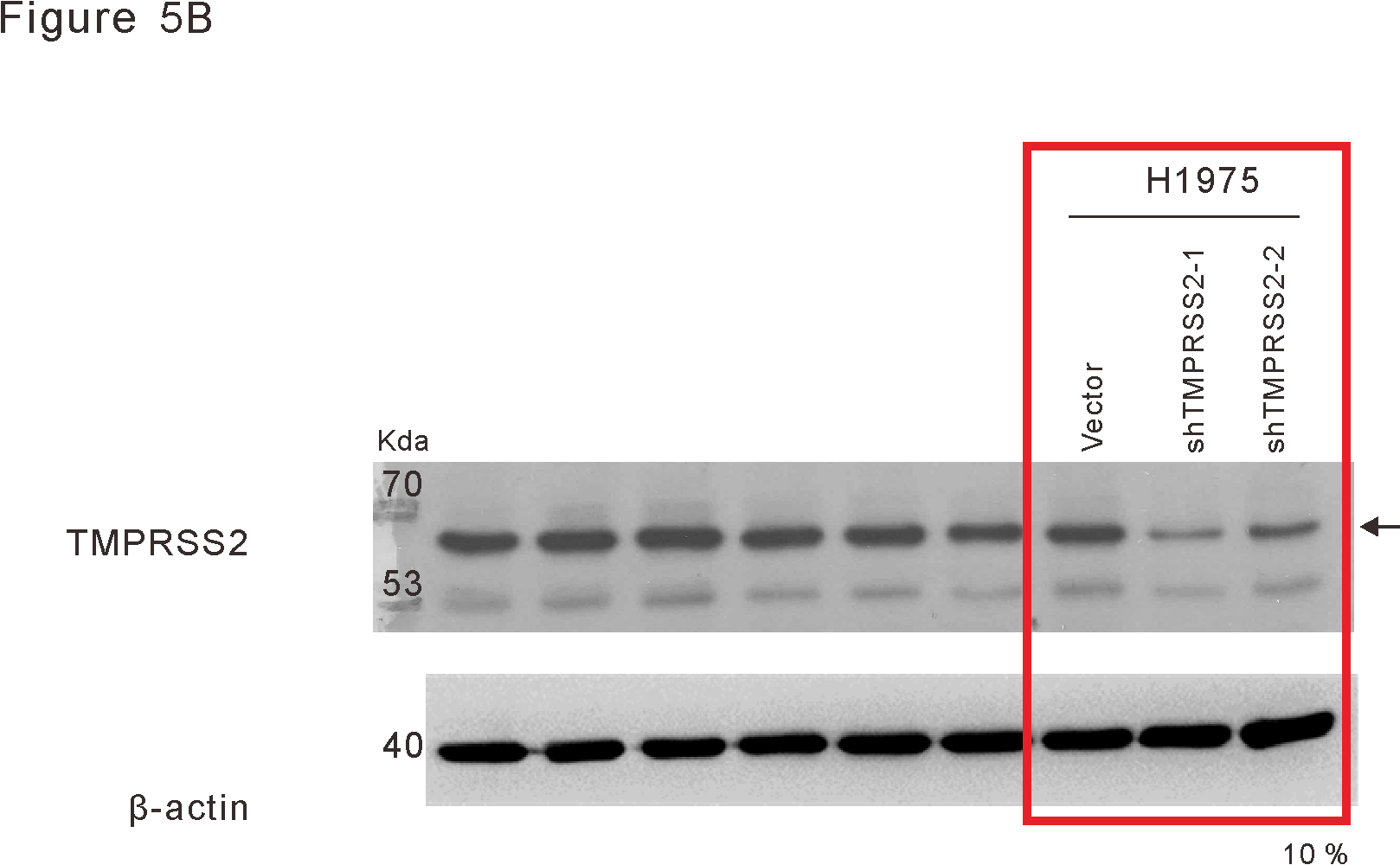

Supplement: Supplementary file 8 — Source Data for Figure 5 [file EMMM-15-e17014-s010.zip › Figure 5/Fig. 5B-Image data.tif]

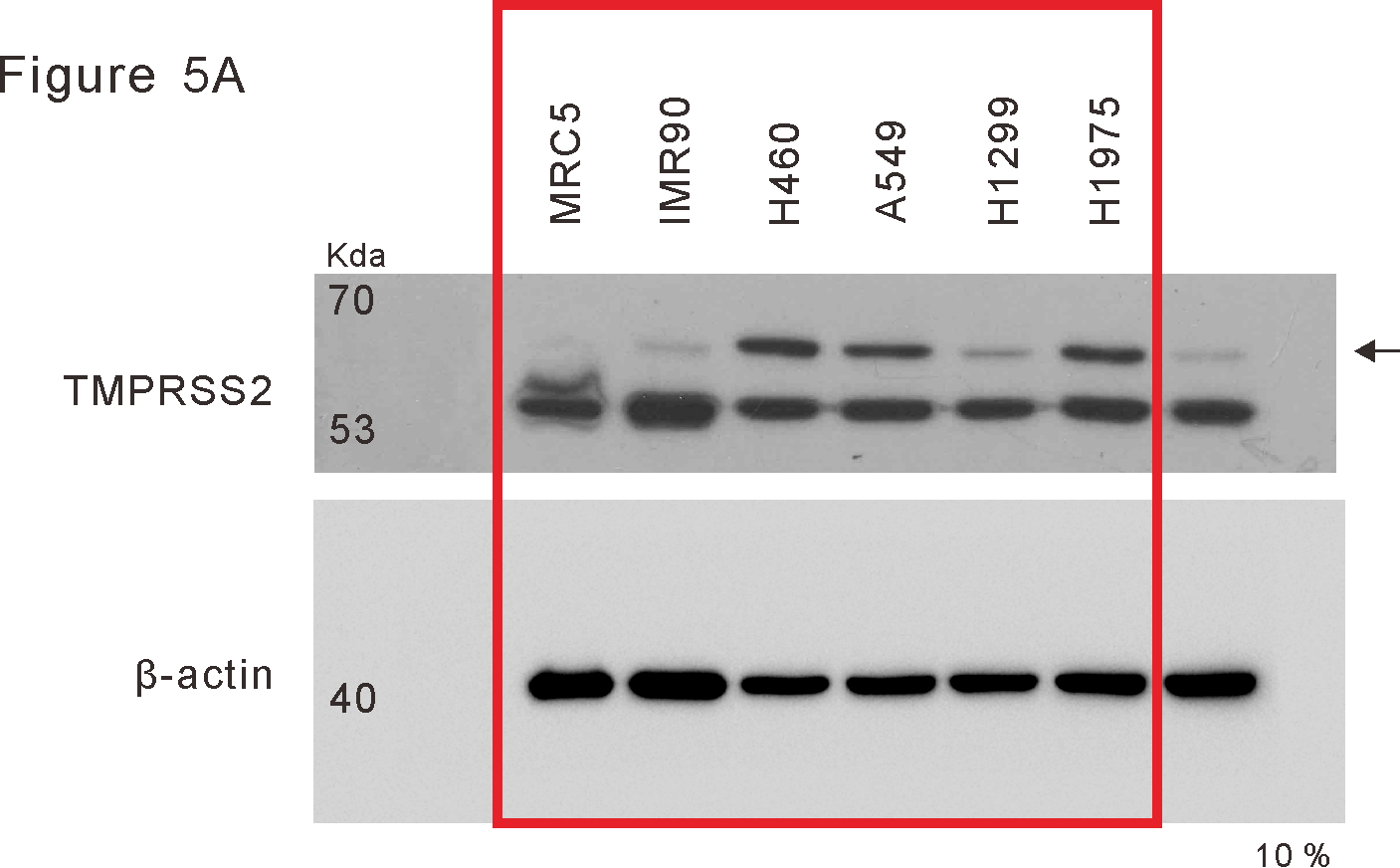

Supplement: Supplementary file 8 — Source Data for Figure 5 [file EMMM-15-e17014-s010.zip › Figure 5/Fig. 5A-Image data.tif]

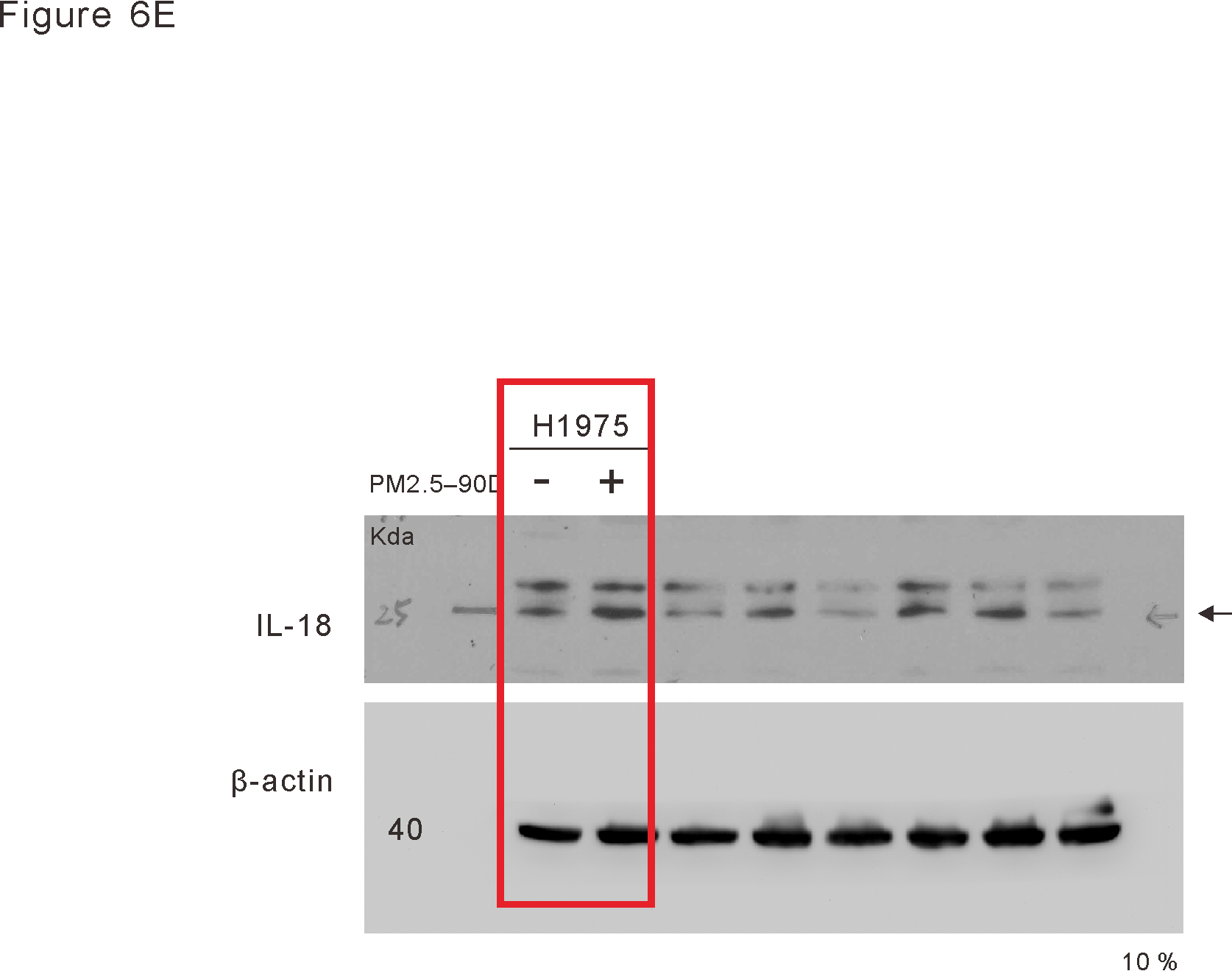

Supplement: Supplementary file 9 — Source Data for Figure 6 [file EMMM-15-e17014-s011.zip › Figure 6/Fig. 6E-Image data.tif]

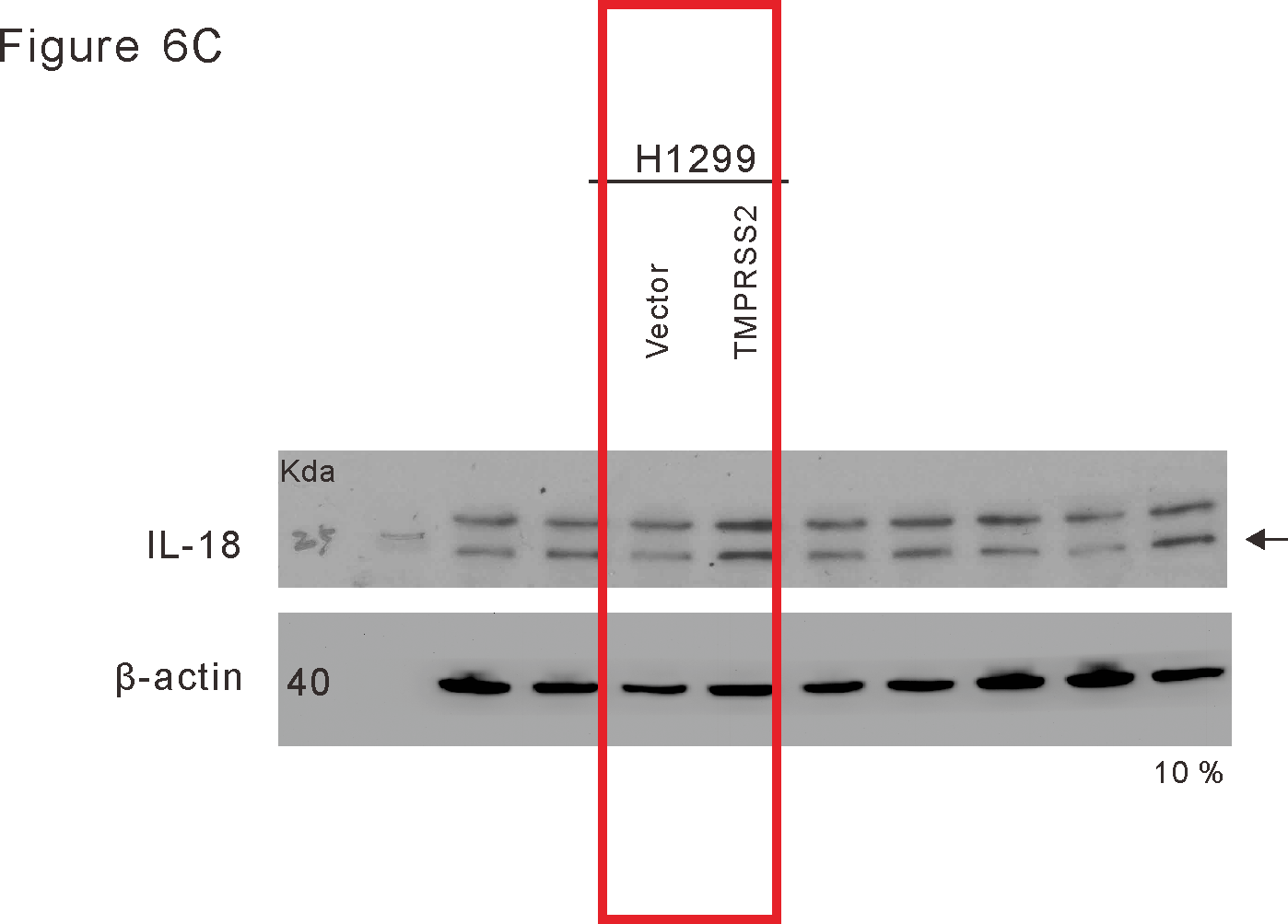

Supplement: Supplementary file 9 — Source Data for Figure 6 [file EMMM-15-e17014-s011.zip › Figure 6/Fig. 6C-Image data.tif]

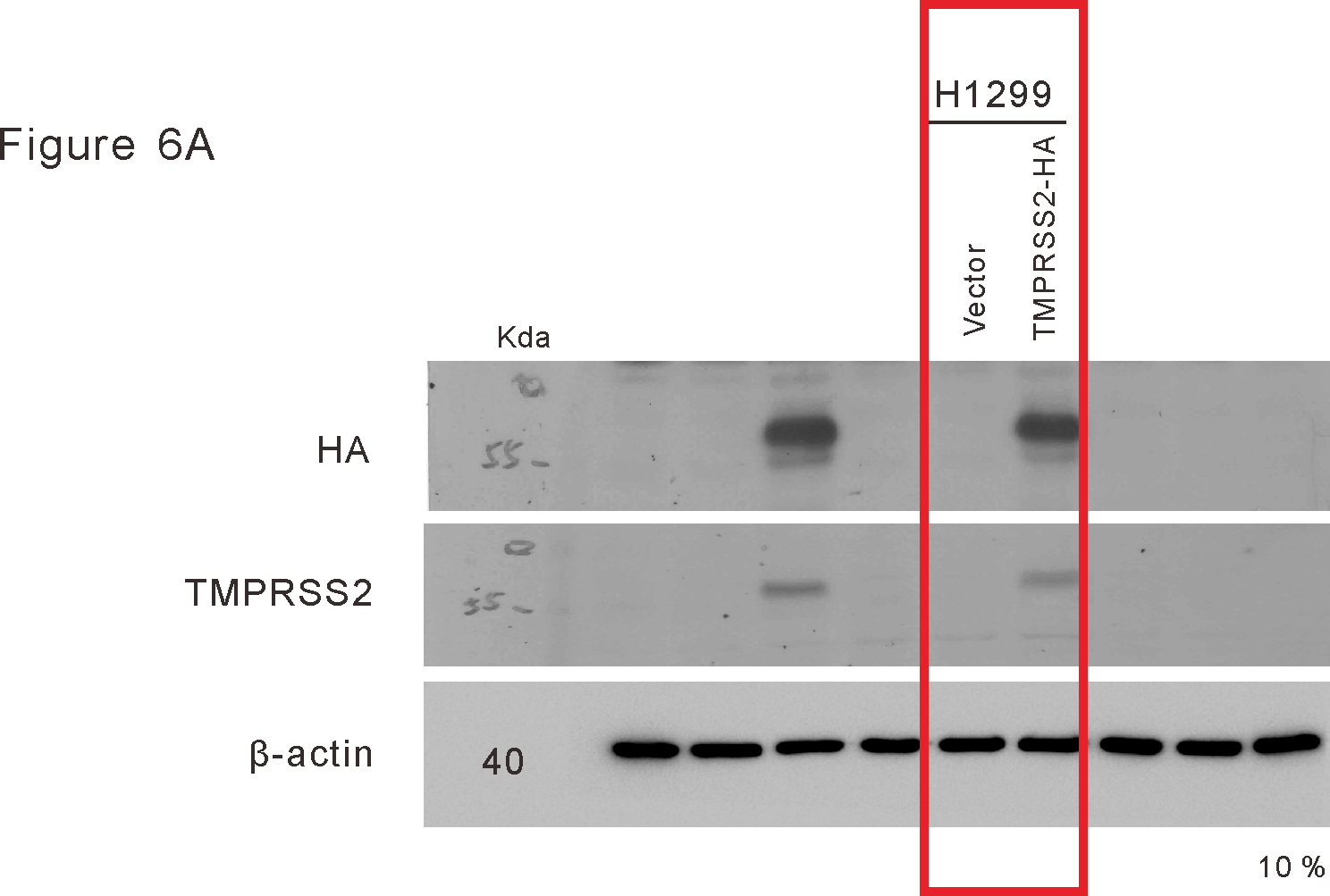

Supplement: Supplementary file 9 — Source Data for Figure 6 [file EMMM-15-e17014-s011.zip › Figure 6/Fig. 6A-Image data.tif]

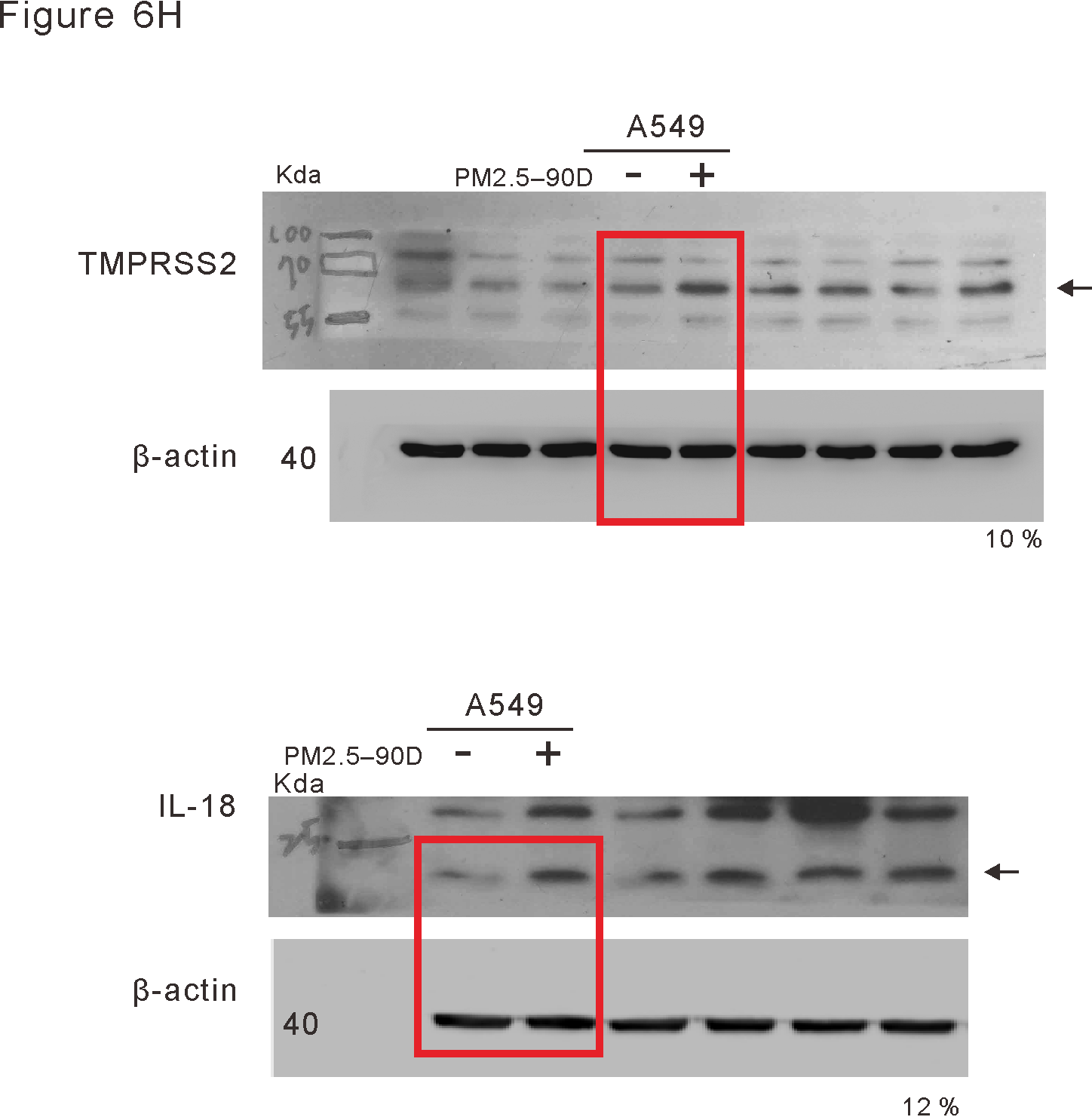

Supplement: Supplementary file 9 — Source Data for Figure 6 [file EMMM-15-e17014-s011.zip › Figure 6/Fig. 6H-Image data.tif]

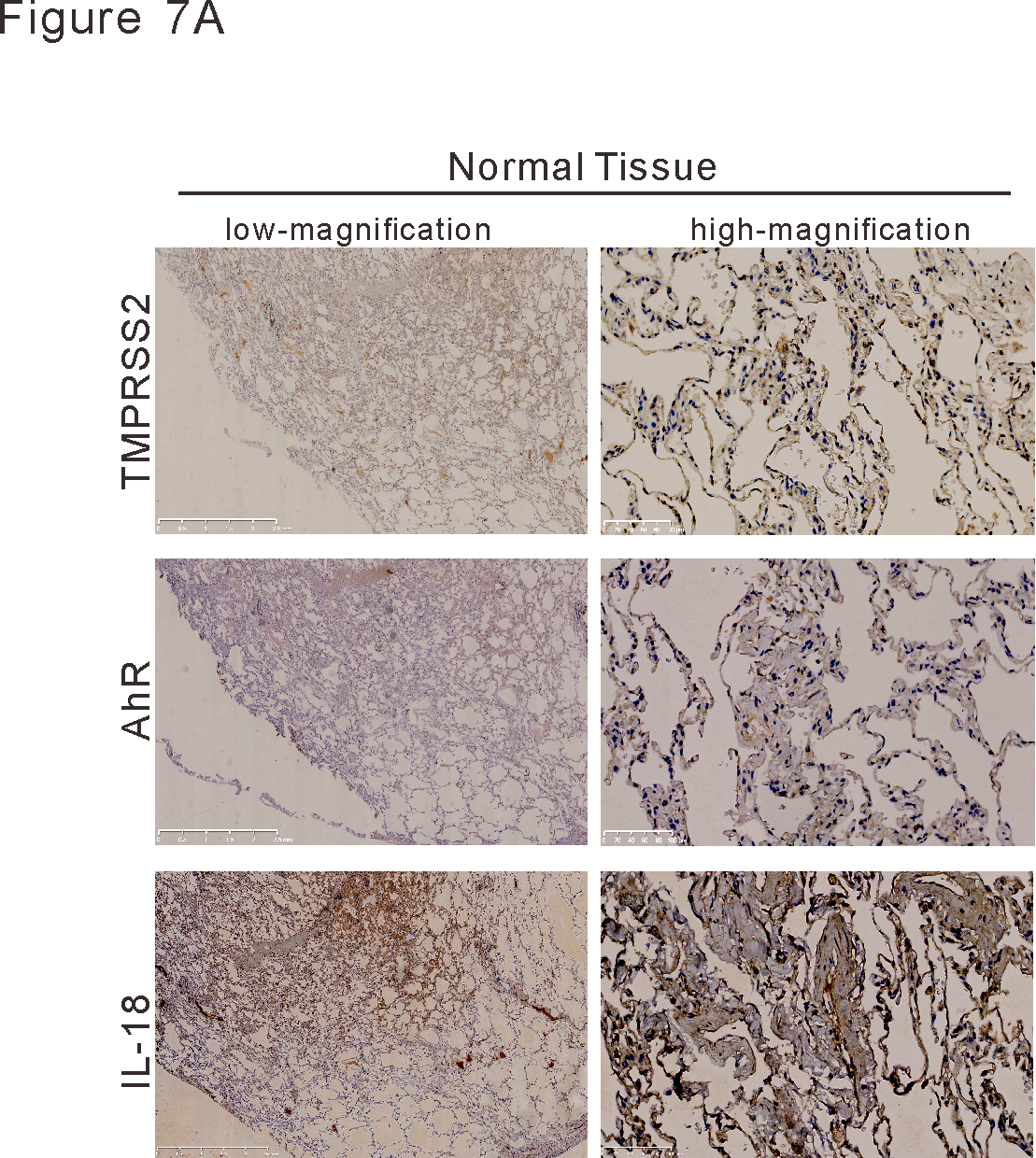

Supplement: Supplementary file 10 — Source Data for Figure 7 [file EMMM-15-e17014-s007.zip › Figure 7/Fig. 7A-Image data.tif]

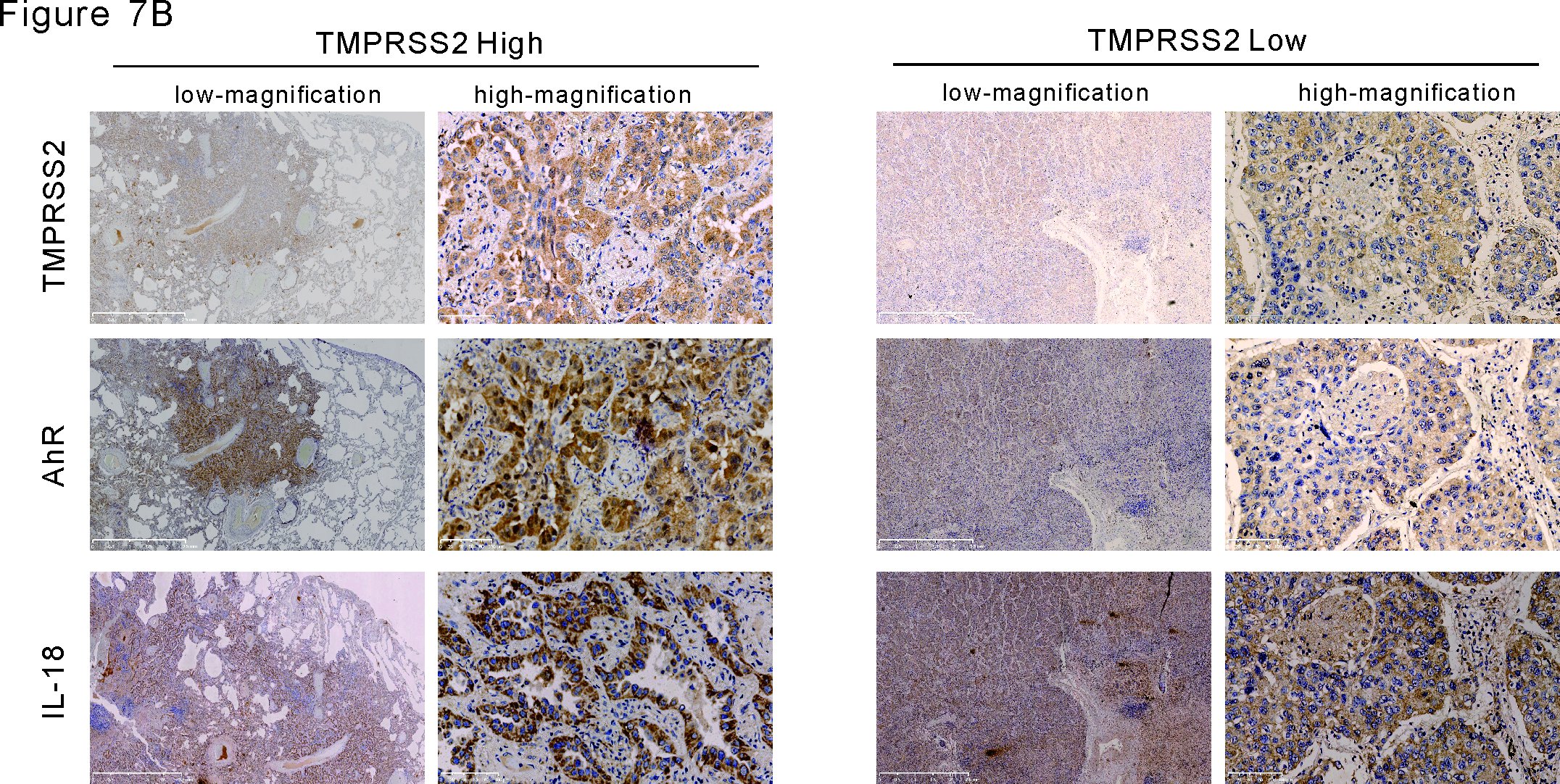

Supplement: Supplementary file 10 — Source Data for Figure 7 [file EMMM-15-e17014-s007.zip › Figure 7/Fig. 7B-Image data.tif]
