# Supplementary material for: PM2.5 promotes lung cancer progression through activation of the AhR‐TMPRSS2‐IL18 pathway
Source: EMBO Mol Med. 2023 Mar 28;15(6):e17014. doi: 10.15252/emmm.202217014 (PMC10245036; doi:10.15252/emmm.202217014)
Supplement: Supplementary file 11 [file EMMM-15-e17014-s009.pdf]

EMBO
Molecular Medicine

PM2.5 promotes lung cancer progression through activation of the AhR-TMPRSS2-IL18 pathway

Tong-Hong Wang, Kuo-Yen Huang, Chin-Chuan Chen, Ya-Hsuan Chang, Hsuan-Yu Chen, Chuen Hsueh, Yi-Tsen Liu, Shuenn-Chen Yang, Pan-Chyr Yang, and Chi-Yuan Chen
DOI: 10.15252/emmm.202217014

Corresponding authors: Pan-Chyr Yang (pcyang@ntu.edu.tw) , Chi-Yuan Chen (d49417002@gmail.com)

Review Timeline:

Submission Date::	20th Oct 22
Editorial Decision:	22nd Nov 22
Revision Received:	17th Feb 23
Editorial Decision:	7th Mar 23
Revision Received:	10th Mar 23
Accepted:	14th Mar 23

Editor: Lise Roth

Transaction Report:

(Note: With the exception of the correction of typographical or spelling errors that could be a source of ambiguity, letters and reports are not edited. Depending on transfer agreements, referee reports obtained elsewhere may or may not be included in this compilation. Referee reports are anonymous unless the Referee chooses to sign their reports.)

22nd Nov 2022

Dear Prof. Yang,

Thank you for the submission of your manuscript to EMBO Molecular Medicine, and please accept my apologies for the delay in getting back to you as one referee needed more time to complete his/her report. We have now received feedback from the three reviewers who agreed to evaluate your manuscript. As you will see below, the reviewers raise substantial concerns on your work, which unfortunately preclude its publication in EMBO Molecular Medicine in its current form.

The reviewers find that the question addressed by the study is of potential interest, however they remain unconvinced that some of the major conclusions are sufficiently supported by the data. They thus raise the following major issues:
- cellular model of long-term exposure reminiscent of a selection process
- mice have not been exposed to PM2.5
- lack of evidence that the patients were exposed to PM2.5

We further cross-commented with the referees and concluded that clear statements and discussion regarding the animal models limitations would be required in a revised version of the current manuscript. Furthermore, it would be appreciated if you could provide environmental information on the patients. If not feasible, this point should be adequately discussed in the manuscript.

If you feel you can satisfactorily address these points and those listed by the referees, you may wish to submit a revised version of your manuscript. Please attach a covering letter giving details of the way in which you have handled each of the points raised by the referees. A revised manuscript will once again be subject to review and we cannot guarantee at this stage that the eventual outcome will be favorable.

If you would like to discuss further the points raised by the referee, I am available to do so via email or video. Let me know if you are interested in this option.

Addressing the reviewers' concerns in full will be necessary for further considering the manuscript in our journal, and acceptance of the manuscript will entail a second round of review. EMBO Molecular Medicine encourages a single round of revision only and therefore, acceptance or rejection of the manuscript will depend on the completeness of your responses included in the next, final version of the manuscript. For this reason, and to save you from any frustrations in the end, I would strongly advise against returning an incomplete revision.

Revised manuscripts should be submitted within three months of a request for revision; they will otherwise be treated as new submissions, except under exceptional circumstances in which a short extension is obtained from the editor.

When submitting your revised manuscript, please carefully review the instructions that follow below. We perform an initial quality control of all revised manuscripts before re-review; failure to include requested items will delay the evaluation of your revision.

We require:

1) A .docx formatted version of the manuscript text (including legends for main figures, EV figures and tables). Please make sure that the changes are highlighted to be clearly visible.

2) Individual production quality figure files as .eps, .tif, .jpg (one file per figure). For guidance, download the 'Figure Guide PDF' (https://www.embopress.org/page/journal/17574684/authorguide#figureformat).

3) At EMBO Press we ask authors to provide source data for the main and EV figures. Our source data coordinator will contact you to discuss which figure panels we would need source data for and will also provide you with helpful tips on how to upload and organize the files.
Numerical data should be provided as individual .xls or .csv files (including a tab describing the data). For blots or microscopy, uncropped images should be submitted (using a zip archive if multiple images need to be supplied for one panel). Additional information on source data and instruction on how to label the files are available at
.

4) A .docx formatted letter INCLUDING the reviewers' reports and your detailed point-by-point responses to their comments. As part of the EMBO Press transparent editorial process, the point-by-point response is part of the Review Process File (RPF), which will be published alongside your paper.

5) A complete author checklist, which you can download from our author guidelines (https://www.embopress.org/page/journal/17574684/authorguide#submissionofrevisions). Please insert information in the

checklist that is also reflected in the manuscript. The completed author checklist will also be part of the RPF.

6) Please note that all corresponding authors are required to supply an ORCID ID for their name upon submission of a revised manuscript.

7) It is mandatory to include a 'Data Availability' section after the Materials and Methods. Before submitting your revision, primary datasets produced in this study need to be deposited in an appropriate public database, and the accession numbers and database listed under 'Data Availability'. Please remember to provide a reviewer password if the datasets are not yet public (see https://www.embopress.org/page/journal/17574684/authorguide#dataavailability).

In case you have no data that requires deposition in a public database, please state so in this section. Note that the Data Availability Section is restricted to new primary data that are part of this study.

8) For data quantification: please specify the name of the statistical test used to generate error bars and P values, the number (n) of independent experiments (specify technical or biological replicates) underlying each data point and the test used to calculate p-values in each figure legend. The figure legends should contain a basic description of n, P and the test applied. Graphs must include a description of the bars and the error bars (s.d., s.e.m.). Please provide exact p values.

9) Our journal encourages inclusion of *data citations in the reference list* to directly cite datasets that were re-used and obtained from public databases. Data citations in the article text are distinct from normal bibliographical citations and should directly link to the database records from which the data can be accessed. In the main text, data citations are formatted as follows: "Data ref: Smith et al, 2001" or "Data ref: NCBI Sequence Read Archive PRJNA342805, 2017". In the Reference list, data citations must be labeled with "[DATASET]". A data reference must provide the database name, accession number/identifiers and a resolvable link to the landing page from which the data can be accessed at the end of the reference. Further instructions are available at .

10) We replaced Supplementary Information with Expanded View (EV) Figures and Tables that are collapsible/expandable online. A maximum of 5 EV Figures can be typeset. EV Figures should be cited as 'Figure EV1, Figure EV2" etc... in the text and their respective legends should be included in the main text after the legends of regular figures.
- For the figures that you do NOT wish to display as Expanded View figures, they should be bundled together with their legends in a single PDF file called *Appendix*, which should start with a short Table of Content. Appendix figures should be referred to in the main text as: "Appendix Figure S1, Appendix Figure S2" etc.
- Additional Tables/Datasets should be labeled and referred to as Table EV1, Dataset EV1, etc. Legends have to be provided in a separate tab in case of .xls files. Alternatively, the legend can be supplied as a separate text file (README) and zipped together with the Table/Dataset file.
See detailed instructions here:

11) The paper explained: EMBO Molecular Medicine articles are accompanied by a summary of the articles to emphasize the major findings in the paper and their medical implications for the non-specialist reader. Please provide a draft summary of your article highlighting
- the medical issue you are addressing,
- the results obtained and
- their clinical impact.
This may be edited to ensure that readers understand the significance and context of the research. Please refer to any of our published articles for an example.

12) For more information: There is space at the end of each article to list relevant web links for further consultation by our readers. Could you identify some relevant ones and provide such information as well? Some examples are patient associations, relevant databases, OMIM/proteins/genes links, author's websites, etc...

13) Author contributions: CRediT has replaced the traditional author contributions section because it offers a systematic machine readable author contributions format that allows for more effective research assessment. Please remove the Authors Contributions from the manuscript and use the free text boxes beneath each contributing author's name in our system to add specific details on the author's contribution. More information is available in our guide to authors.

14) Conflict of interest: We updated our journal's competing interests policy in January 2022 and request authors to consider both actual and perceived competing interests. Please review the policy https://www.embopress.org/competing-interests and update your competing interests if necessary.

15) Every published paper now includes a 'Synopsis' to further enhance discoverability. Synopses are displayed on the journal webpage and are freely accessible to all readers. They include a short stand first (maximum of 300 characters, including space) as well as 2-5 one-sentences bullet points that summarizes the paper. Please write the bullet points to summarize the key NEW findings. They should be designed to be complementary to the abstract - i.e. not repeat the same text. We encourage inclusion

of key acronyms and quantitative information (maximum of 30 words / bullet point). Please use the passive voice. Please attach these in a separate file or send them by email, we will incorporate them accordingly.

Please also suggest a striking image or visual abstract to illustrate your article as a PNG file 550 px wide x 300-600 px high.

16) As part of the EMBO Publications transparent editorial process initiative (see our Editorial at http://embomolmed.embopress.org/content/2/9/329), EMBO Molecular Medicine will publish online a Review Process File (RPF) to accompany accepted manuscripts.
In the event of acceptance, this file will be published in conjunction with your paper and will include the anonymous referee reports, your point-by-point response and all pertinent correspondence relating to the manuscript. Let us know whether you agree with the publication of the RPF and as here, if you want to remove or not any figures from it prior to publication.
Please note that the Authors checklist will be published at the end of the RPF.

EMBO Molecular Medicine has a "scooping protection" policy, whereby similar findings that are published by others during review or revision are not a criterion for rejection. Should you decide to submit a revised version, I do ask that you get in touch after three months if you have not completed it, to update us on the status.

I look forward to receiving your revised manuscript.

Yours sincerely,

Lise Roth

Lise Roth, PhD
Senior Editor
EMBO Molecular Medicine

Please note: When submitting your revision you will be prompted to enter your funding and payment information. This will allow Wiley to send you a quote for the article processing charge (APC) in case of acceptance. This quote takes into account any reduction or fee waivers that you may be eligible for. Authors do not need to pay any fees before their manuscript is accepted and transferred to the publisher.

EMBO Press participates in many Publish and Read agreements that allow authors to publish Open Access with reduced/no publication charges. Check your eligibility: https://authorservices.wiley.com/author-resources/Journal-Authors/open-access/affiliation-policies-payments/index.html

***** Reviewer's comments *****

Referee #1 (Remarks for Author):

The manuscript reports how PM2.5 promotes lung cancer progression through activation of the AhR-TMPRSS2-IL18 by investigating the molecular mechanisms of PM2.5 exposure using a validated models.

Key points from this article include that (i) short-term exposure to PM2.5 for 24 h activated the EGFR pathway in lung cancer cells (EGFR wild-type and mutant), (ii) long-term exposure of lung cancer cells to PM2.5 for 90 days persistently promoted EGFR activation, (iii) while also promoting cell proliferation, anchorage-independent growth and tumor growth in a mouse model.

The results of this study are being presented and written in a cohesive flow. Materials and methods and figure legends have necessary overall details.

However, the following improvements to the introduction and statistical analysis portion can further improve the manuscript.

• Update WHO statistics of premature deaths to more recent numbers.

• Ensure that proper references are cited for the following statements:
o "Short term or long-term exposure to PM are known to cause adverse health effects, including cardiovascular and respiratory disease, and cancers."
o "PM10 is mostly deposited in the nasal cavity and upper respiratory tract, while PM2.5 can enter the lower respiratory tract into the alveoli and enter the bloodstream"
o "In addition, PAHs are known to photochemically react with sulfur oxides, nitrogen oxides, and ozone in the air to form secondary pollutants that are carcinogenic and genotoxic to the human body."

• Spell out TMPRSS2 the first time is mentioned.

• Provide reference for the following statement, "Although many epidemiological studies have confirmed that PM2.5 is a risk factor for the occurrence of lung cancer..."

• Correct grammatical error so the sentence reads as: "Culture media and chemical compounds were purchased..." (Also, please spell-check the document in case there are others)

• Describe the statistical analysis in detail for each of the experiments.

• Specify if any covariates were considered or if any adjustments for multiple hypothesis testing were done.

• Clarify if data were normally distributed and if any non-parametric tests were considered.

Referee #2 (Comments on Novelty/Model System for Author):

I think the topic is interesting, but the results are not cohesive enough.

Referee #2 (Remarks for Author):

The manuscript by Wang et al. showed that long-term exposure to PM2.5 promoted the growth of lung cancer cells in vitro and in vivo. They also found that AhR translocation, TMPR22 and IL18 expression were increased in the lung cancer cells exposed to PM2.5. Accumulating epidemiological studies have shown that PM2.5 is associated with the progression of lung cancer; however, it is still unclear how PM2.5 affects tumor growth. This study has provided evidence that long-term exposure of PM2.5 accelerated tumor growth and its potential molecular mechanism. The topic is of interest and will attract a wide readership. However, there are several major concerns that need to be addressed.

Major comments:
1. According to Fig. 1A, when lung cancer cells were treated with PM2.5 for 24 hours, the IC50 for H1975 was 55.35 ug/ml, but when the authors established long-term exposed cell lines, 50 ug/ml of PM2.5 was used. How would H1975 cells survive at this concentration? The strategy of establishing long-term exposure used in this study is more like the selection of cancer cells with growth advantage. How is 50 ug/ml of PM2.5 correlated with the level of PM2.5 that people might inhale?

2. More lung cancer cell lines are required to verify the effects of PM2.5 on cancer cell growth and tumorigenesis.

3. Can AhR translocation also be observed in the A549 cells exposed to PM2.5? Immunofluorescence staining of AhR to further verify its nuclear localization in both cell lines (H1975 and A549) is recommended.

4. It was nice to see TMPRSS2 was increased and the binding of AhR on the promoter of TMPRSS2 in the PM2.5-90D H1975 cells. It would be great if the authors can show whether PM2.5-induced TMPRSS2 expression is mediated by AhR.

5. Although the authors showed that TMPRSS2 was important for the growth of lung cancer cells (Fig.5), whether PM2.5 exposure-induced tumorigenesis was dependent on TMPRSS2 was not demonstrated here.

6. In Fig 6, the IL18 mRNA change is marginal, IL18 protein level needs quantitation and statistics. How is IL18 related to PM2.5-promoted tumorigenesis?

Minor comments:
1. Right panel of Fig. 3C (TMPRSS2 IHC) was not mentioned in the text.
2. p.4 line 8: derived from the PM2.5-exposed cells compared to the tumors derived from the untreated cells (Figure 3D). There is no Fig. 3D.

Referee #3 (Remarks for Author):

The authors Wang et al., submitted an original research article entitled "PM2.5 promotes lung cancer progression through activation of the AhR-TMPRSS2-IL18 pathway".
The manuscript demonstrated that PM2.5 activated EGFR signaling in lung cancer cell lines under short- and long-term exposure to PM2.5. Moreover, cytoplasmic aryl hydrocarbon receptor (AhR) was up-regulated in H1975 cells with long-term exposure to PM2.5. AhR then bound to promoter region of TMPRSS2 leading to up-regulation of TMPRSS2 in transcriptomic and proteomic level. Activation of TMPRSS2 result in elevating expression of IL18. This novel finding of PM2.5 promoting lung cancer progression via AhR-TMPRSS2-IL18 axis was interesting and may provide potential therapeutic strategy. However, lack of environmental information of lung cancer patients evaluates these patients who had exposure to PM2.5 previously. Thus, this manuscript may need to be revised before publishing.

Major concerns:
1. The author used long-term PM2.5-treated H1975 lung cancer cells to conduct majority of in vitro and in vivo experiments to demonstrate that PM2.5 promoted lung cancer progression. However, in figure 1A, the cell viability results indicated that PM2.5 had cytotoxicity to H1975 cells. In this case, long-term exposure to PM2.5 could be a selection processing to generate "PM2.5 resistant clone of H1975 cells". Using this PM2.5 treated H1975 cells to perform in vivo mouse study would be inappropriate to claim PM2.5 promote lung cancer progression since these mice were not really exposure to PM2.5. The sentences may need to be revised in manuscript.

2. In figure 7, even though the results showed that the expression correlation between TMPRSS2 and AhR in lung cancer patients, however, no evidence indicated that these patients were exposed to high PM2.5 environment or high concentration of PM2.5 accumulated in lung of these patients. It is difficult to be convinced that the high expression of AhR and TMPRSS2 were due to PM2.5 exposure. The high expression of AhR and TMPRSS2 may be induced by other factors.

Minor concerns:
1. In result section, IC50s for cell types were mentioned but did not provide figures or cited references.
2. Even though the concentration of PM2.5 was based on IC50 results, what would be the rationale to use 50 μg/ml PM2.5 for long-term exposure? Is it representable as patient exposure to high M2.5 environment?
3. In figure 4D, please provide IgG control for ChIP-qPCR results.
4. Please provide access number of RNA sequencing data in the Gene Expression Omnibus (GEO) repository in method section.
5. The expression of IL18 was increased in high TMPRSS2 cell lines. It would be better to demonstrate that IL18 expression is also up-regulated in high TMPRSS2 patients and xenograft tumors.

1). To review GEO accession GSE220252:
https://www.ncbi.nlm.nih.gov/geo/query/acc.cgi?acc=GSE220252
Token: ynwnuagkrzupbkh
2). To review GEO accession GSE220306:
https://www.ncbi.nlm.nih.gov/geo/query/acc.cgi?acc=GSE220306
Token: sryrqcmwbvedbqv

Dear Sir,

The authors would like to thank you for kindly providing us the opportunity to revise our manuscript entitled "PM2.5 promotes lung cancer progression through activation of the AhR-TMPRSS2-IL18 pathway" by Wang *et al.*. We appreciate the expert comments and suggestions from the Editor and the Reviewers, and have carefully revised our manuscript as suggested. Our point-by-point responses to the Reviewer's comments are shown below.

Our point-by-point responses to the comments from **Reviewer #1** are as follows:
Comment 1. Update WHO statistics of premature deaths to more recent numbers
Response: We thank the reviewer for the comment. We have updated WHO statistics of premature deaths in the revised Introduction (see p. 2, lines 21-26).

Comment 2. Ensure that proper references are cited for the following statements:
o "Short term or long-term exposure to PM are known to cause adverse health effects, including cardiovascular and respiratory disease, and cancers."
o "PM10 is mostly deposited in the nasal cavity and upper respiratory tract, while PM2.5 can enter the lower respiratory tract into the alveoli and enter the bloodstream"
o "In addition, PAHs are known to photochemically react with sulfur oxides, nitrogen oxides, and ozone in the air to form secondary pollutants that are carcinogenic and genotoxic to the human body."
Response: We thank the reviewer for valuable suggestions. We have added proper references in the revised manuscript (see p. 2, lines 29, 33, and 47).

Comment 3. Spell out TMPRSS2 the first time is mentioned.
Response: We thank the reviewer for the suggestion. We have added the full name of TMPRSS2 in the revised manuscript (see p. 3, lines 6-7).

Comment 4. Provide reference for the following statement, "Although many epidemiological studies have confirmed that PM2.5 is a risk factor for the occurrence of lung cancer..."

Response: We thank the reviewer for the comment. We have added two references in the revised manuscript (see p. 3, line 11).

Comment 5. Correct grammatical error so the sentence reads as: "Culture media and chemical compounds were purchased..." (Also, please spell-check the document in case there are others)
Response: We have corrected the grammatical error as suggested (p.9, line 6).

Comment 6. Describe the statistical analysis in detail for each of the experiments.
Response: We thank the reviewer for this critical issue. The description for the statistical analysis in detail for each of the experiments has been added in the section of Materials and Methods (see p. 10, lines 26-48, and p.11 lines 1-4).

Comment 7. Specify if any covariates were considered or if any adjustments for multiple hypothesis testing were done.
Response: Thanks for reviewer comments. To considering covariates and issues of multiple hypotheses testing, the following statistic methods were used in this study and revised in the section of statistical analysis (see p. 10, lines 26-48, and p.11 lines 1-4):
(1) One-way ANOVA with Tukey's multiple comparisons test of post-hoc analysis.
(2) Welch's ANOVA and Games-Howell test of post-hoc analysis.
(3) Two-way ANOVA and Tukey's multiple comparisons test of post-hoc analysis.
(4) Two-way repeated measures ANOVA with pairwise comparison of post-hoc analysis with Benjamini-Hochberg (BH) correction.
(5) If multiple statistics tests conducted in one experiment, Benjamini-Hochberg (BH) method for adjusting multiple hypothesis testing were done.

Comment 8. Clarify if data were normally distributed and if any non-parametric tests were considered.
Response: Normal distribution was tested by Shapiro-Wilk normality test. Data in this study were all followed normal distribution because *p*-values of all tests were > 0.05. The description of data normality examination was included in the revised section of statistical analysis (see p. 10, lines 26-48, and p.11 lines 1-4).

Our point-by-point responses to the comments from **Reviewer #2** are as follows:
Major comment 1. According to Fig. 1A, when lung cancer cells were treated with PM2.5 for 24 hours, the IC50 for H1975 was 55.35 ug/ml, but when the authors established long-term exposed cell lines, 50 ug/ml of PM2.5 was used. How would H1975 cells survive at this concentration? The strategy of establishing long-term exposure used in this study is more like the selection of cancer cells with growth

advantage. How is 50 ug/ml of PM2.5 correlated with the level of PM2.5 that people might inhale?

Response: Thanks for raising this critical question. The concentration 35 μg/m^3 of PM2.5 in the air is considered to be unhealthy when exposed during a 24-h period. This concentration is about 1.4 million-fold lower than the 50 μg/ml of PM2.5 used in this study. However, continuous exposure to the low level of PM2.5 in the air could result to the accumulation of high concentrations of PM2.5 in the targeted cells. To experimentally determine the potentially harmful effects of PM2.5, high concentrations of PM2.5 were employed by most investigators. The PM2.5 dosage used for *in vitro* experiments in this work was based on several previous studies on PM2.5-induced cytotoxicity (*Autophagy.* 2016, 12: 1832-1848, *Arch Toxicol.* 2018, 92: 2077-2091, and *Front Physiol. 2019*, 10: 1404). For H1975 cells treated with 50 μg/mL of PM2.5 for 24 h, the viability is about 60% (Figure 1A). Therefore, it is possible the survivors after long-term exposure to PM2.5 may have been selected for growth advantage as suggested by the Reviewer. Nevertheless, these long-term exposed cells were useful to unravel the signal pathways (eg, EGFR and AhR-TMPRSS2-IL18 pathway) that may promote the disease progression caused by PM2.5.

Major comment 2. More lung cancer cell lines are required to verify the effects of PM2.5 on cancer cell growth and tumorigenesis.

Response: We thank the reviewer for the suggestion. We have treated another lung cell line (PC9) with 50 μg/ml of PM2.5 for 60 days and shown that these long-term exposed cells have increased ability to proliferate and to undergo anchorage-independent growth. This result has been added in the revised manuscript as new Figure EV1, and is described on p. 3, lines 45-47.

Major comment 3. Can AhR translocation also be observed in the A549 cells exposed to PM2.5? Immunofluorescence staining of AhR to further verify its nuclear localization in both cell lines (H1975 and A549) is recommended.

Response: We thank the reviewer for the suggestion. As suggested by the reviewer, we have performed the immunofluorescence staining of AhR in A549 and H1975 cells. The results showed that elevated nuclear localization of AhR was evident in the PM2.5-treated cells. These results have been added in the revised manuscript as new Figure EV3 and described in p. 4, lines 47-48, and p. 5, lines 1-2.

Major comment 4. It was nice to see TMPRSS2 was increased and the binding of AhR on the promoter of TMPRSS2 in the PM2.5-90D H1975 cells. It would be great if the authors can show whether PM2.5-induced TMPRSS2 expression is mediated by AhR.

Response: We thank the reviewer for the suggestion. We have employed AhR antagonist CH223191 to examine the modulation the PM2.5-induced TMPRSS2 expression by AhR. The results indicate that AhR antagonist (CH223191) inhibited the expression level of TMPRSS2 in PM2.5-treated cells. These results have been added in the revised manuscript as the new Figure 4E and described in p. 5 lines 8-10.

Major comment 5. Although the authors showed that TMPRSS2 was important for the growth of lung cancer cells (Fig.5), whether PM2.5 exposure-induced tumorigenesis was dependent on TMPRSS2 was not demonstrated here.
Response: We thank the reviewer for the comment. PM2.5 exposure-induced tumorigenesis may include initiation and promotion. This work focused on the promotion effects of PM2.5 exposure on lung cancer cells. Our results have shown that long-term exposure to PM2.5 promoted tumor growth and TMPRSS2 was one of the important key players for the promotion of tumor growth in lung cancer cells (Figure 5). The other possibilities of PM2.5 exposure-induced tumorigenesis was not tested in the present study.

Major comment 6. In Fig 6, the IL18 mRNA change is marginal, IL18 protein level needs quantitation and statistics. How is IL18 related to PM2.5-promoted tumorigenesis?
Response: We thank the reviewer for the comments. The quantitation and statistics analysis of IL18 protein has been added to the revised Figure 6E and 6H. The possible involvement of IL-18 in PM2.5-promoted tumorigenesis has been discussed in the Discussion (p.8, 6-16).

Minor comment 1. Right panel of Fig. 3C (TMPRSS2 IHC) was not mentioned in the text.
Response: We thank you for pointing out this mistake. We have corrected the mistake in the revised manuscript (see p. 4, lines 14-16).

Minor comment 2. p.4 line 8: derived from the PM2.5-exposed cells compared to the tumors derived from the untreated cells (Figure 3D). There is no Fig. 3D.
Response: We thank you for pointing out this mistake. We have corrected the mistake in the revised manuscript (see p. 4, line 16).

Our point-by-point responses to the comments from **Reviewer #3** are as follows:
Major concern 1. The author used long-term PM2.5-treated H1975 lung cancer cells to conduct majority of in vitro and in vivo experiments to demonstrate that PM2.5 promoted lung cancer progression. However, in figure 1A, the cell viability results indicated that PM2.5 had cytotoxicity to H1975 cells. In this case, long-term exposure to PM2.5 could be a selection processing to generate "PM2.5 resistant clone of H1975 cells". Using this PM2.5 treated H1975 cells to perform in vivo mouse study would be inappropriate to claim PM2.5 promote lung cancer progression since these mice were not really exposure to PM2.5. The sentences may need to be revised in manuscript.
Response: We thank the reviewer for the comment. We rewrote the heading sentence (p. 4, lines 2-3) to make it clear that we are using the xenograft model to examine the effects of long-term PM2.5 exposure to H1975 cells on their ability to proliferate *in vivo*. The results indicate that long-term exposure of lung cancer cells to PM2.5 enhanced the tumorigenic abilities *in vivo*.

Major concern 2. In figure 7, even though the results showed that the expression correlation between TMPRSS2 and AhR in lung cancer patients, however, no evidence

indicated that these patients were exposed to high PM2.5 environment or high concentration of PM2.5 accumulated in lung of these patients. It is difficult to be convinced that the high expression of AhR and TMPRSS2 were due to PM2.5 exposure. The high expression of AhR and TMPRSS2 may be induced by other factors.

Response: We thank the reviewer for this critical issue. In the "Clinical correlation of TMPRSS2 and IL18 expression with nuclear AhR and overall cancer staging in human lung cancer specimens", we evaluated the role of TMPRSS2 expression in lung cancer progression using tumor specimens from lung cancer patients. Since there are no records of PM2.5 exposure in these patients, this study did not address the effects of PM2.5 exposure, it simply addressed if TMPRSS2 expression plays a role in lung cancer progression in humans. Our results revealed that there is a significant correlation between the expression level of TMPRSS2 and nuclear AhR ($p < 0.01$; Table 3). Elevated expression of TMPRSS2 was also positively correlated with the advanced overall stages of lung cancer patients ($p < 0.05$; Table 3). These results suggest that TMPRSS2 and AhR may play a role in the progression of lung cancer in vivo. The factors involved in the high expression of AhR and TMPRSS2 may be due to PM2.5 or other factors as suggested by the reviewer.

Minor concern 1. In result section, IC50s for cell types were mentioned but did not provide figures or cited references.

Response: This information is now provided in the revised manuscript as the new Figure 1A.

Minor concern 2. Even though the concentration of PM2.5 was based on IC50 results, what would be the rationale to use 50 μg/ml PM2.5 for long-term exposure? Is it representable as patient exposure to high PM2.5 environment?

Response: Thanks for raising this critical question. To determine the PM2.5 dose for long-term exposure, we first examined the effects of short-term exposure of PM2.5 over a dose range (0-100 μg/ml) used by other investigators for in vitro experiments (*Ecotoxicol Environ Saf.* 2019, 178:159-167 and *Environ Toxicol.* 2017, 32(11):2341-2351). Based on the results shown in Figure 1, we selected the concentration of 50 μg/ml to perform long-term exposure study.

Minor concern 3. In figure 4D, please provide IgG control for ChIP-qPCR results.

Response: The IgG data is now provided in the revised manuscript as Figure 4D and described in the section of figure legends (see p. 18, lines 18-21).

Minor concern 4. Please provide access number of RNA sequencing data in the Gene Expression Omnibus (GEO) repository in method section.

Response: The RNA sequencing data have been deposited in the Gene Expression Omnibus under the accession GSE220252 and GSE220306. This information is now provided in the section of Materials and Methods (see p. 9, lines 31-33).

1). To review GEO accession GSE220252:

https://www.ncbi.nlm.nih.gov/geo/query/acc.cgi?acc=GSE220252

Enter token ynwnuagkrzupbkh into the box

2). To review GEO accession GSE220306:

https://www.ncbi.nlm.nih.gov/geo/query/acc.cgi?acc=GSE220306
Enter token sryrqcmwbvedbqv into the box

Minor concern 5. The expression of IL18 was increased in high TMPRSS2 cell lines. It would be better to demonstrate that IL18 expression is also up-regulated in high TMPRSS2 patients and xenograft tumors.
Response: We have performed the IHC staining of IL18 in tumor specimens from patients and in xenografted tumors from nude mice. The results showed that elevated expression of IL18 was evident in tumor specimens with high level of TMPRSS2 and in xenograft tumors from PM2.5-treated cells. These results have been added in the revised manuscript as the new Figure 3C, 7A, 7B, EV5, Table 3 and described in p. 4, lines 14-16, p. 6, lines16-29, and p. 8, lines 24-25.

The manuscript has greatly benefited from these insightful suggestions, and we hope that you will find the revised manuscript is now acceptable for publication in EMBO Molecular Medicine.

Thank you for your consideration.

Sincerely yours,

Pan-Chyr Yang, MD, PhD
Professor
Department of Internal Medicine
National Taiwan University College of Medicine
No. 1, Sec. 1, Jen-Ai Road, Taipei 100225, Taiwan
Tel: +886-2-23562905
E-mail: pcyang@ntu.edu.tw, panchyryang@gmail.com

Chi-Yuan Chen, Ph.D.
Professor
Graduate Institute of Health Industry Technology
Chang Gung University of Science and Technology
Tao-Yuan 333, Taiwan
E-mail: d49417002@gmail.com

7th Mar 2023

Dear Prof. Yang,

Thank you for the submission of your revised manuscript to EMBO Molecular Medicine. We have now received the enclosed reports from the two referees who re-reviewed your study. As you will see, they are now supportive of publication, and we will therefore be able to accept your manuscript once the following editorial points will be addressed:

1/ Main manuscript text:
- Please accept the changes and only keep in track changes mode any new modification.
- Materials and methods:
o When referring to methods already published, please make sure the articles are open-access and to nevertheless provide minimal information.
o Cells: please indicate whether cells were tested for mycoplasma contamination.
o Antibodies: please provide the dilutions used.
o Animals: please indicate the housing and husbandry conditions.
o Tumor specimens: please include the sentence that the experiments conformed to the principles set out in the WMA Declaration of Helsinki and the Department of Health and Human Services Belmont Report.
o Statistical analysis: please include a statement about blinding, randomization and inclusion/exclusion criteria.
- Thank you for depositing your datasets in public repository. Please make sure that they are public before online publication.

2/ Figures and Source Data:
- Please provide exact p values, not a range, in the figures or in their legends, including for non-significant p values.
- Thank you for providing Source Data. Please update the SD checklist by checking the boxes when you have provided the requested files.

3/ Checklist:
Please upload your checklist as an excel file. Please select an answer in the middle column, and also fill in the section on the WMA Declaration of Helsinki. Please also make sure that all sections related to Data Availability are adequately filled in.

4/ Thank you for providing The Paper Explained. I included minor modifications, please let me know if you agree with the following, or amend as you see fit:
Problem
Particulate matter 2.5 (PM2.5) is a known risk factor for lung cancer; however, the molecular mechanisms triggered by exposure to PM2.5 and affecting cancer progression are unclear.
Results
In this study, lung cancer cells exposed to PM2.5 for 90 days were used as a cellular model of the effects of long-term exposure to PM2.5 on lung cancer. In mice, tumor progression was enhanced by long-term exposure to PM2.5. The molecular mechanism identified may include (i) activation of EGFR signaling and (ii) activation of AhR to upregulate TMPRSS2, which in turn upregulates its downstream targets, such as IL18.
Impact
Long-term exposure to PM2.5 could promote tumor progression in lung cancer through activation of EGFR and AhR to enhance the TMPRSS2-IL18 pathway.

5/ Synopsis:
Please modify the text of your synopsis: This text should be different from The Paper Explained. You may include up to 5 bullet points summarizing your findings.

Thank you for providing a nice synopsis image. Please upload it as an individual file (without synopsis text) as a PNG/TIF/JPEG file 550 px wide x 300-600 px high, and make sure that the text included in the image remains legible.

6/ As part of the EMBO Publications transparent editorial process initiative (see our Editorial at http://embomolmed.embopress.org/content/2/9/329), EMBO Molecular Medicine will publish online a Review Process File (RPF) to accompany accepted manuscripts.
This file will be published in conjunction with your paper and will include the anonymous referee reports, your point-by-point response and all pertinent correspondence relating to the manuscript. Let us know whether you agree with the publication of the RPF and as here, if you want to remove or not any figures from it prior to publication.
Please note that the Authors checklist will be published at the end of the RPF.

I look forward to receiving your revised manuscript.

With kind regards,

Lise

Lise Roth, PhD
Senior Editor
EMBO Molecular Medicine

.

***** Reviewer's comments *****

Referee #1 (Remarks for Author):

The manuscript reports how PM2.5 promotes lung cancer progression through activation of the AhR-TMPRSS2-IL18 by investigating the molecular mechanisms of PM2.5 exposure using a validated models.

The article outlines several important findings: firstly, that exposure to PM2.5 for 24 hours activates the EGFR pathway in both wild-type and mutant lung cancer cells; secondly, long-term exposure over a period of 90 days persistently promotes EGFR activation; and thirdly, this extended exposure promotes cell proliferation, anchorage-independent growth, and tumor growth in a mouse model.

The study is presented in a clear and organized manner, with comprehensive materials and methods and detailed figure legends provided.

Thank you for addressing most of the comments and suggestions raised during the review process

Referee #3 (Remarks for Author):

The authors addressed all my questions and I recommend the manuscript for publication.

The authors addressed the remaining editorial issues.

14th Mar 2023

Dear Prof. Yang,

Thank you for sending your revised files. I am pleased to inform you that your manuscript is accepted for publication and is now being sent to our publisher to be included in the next available issue of EMBO Molecular Medicine!

Please read below for additional IMPORTANT information regarding your article, its publication and the production process.

Congratulations on your interesting work,

With kind regards,

Lise

Lise Roth, Ph.D
Senior Editor
EMBO Molecular Medicine

Follow us on Twitter @EmboMolMed
Sign up for eTOCs at embopress.org/alertsfeeds

*** *** *** IMPORTANT INFORMATION *** *** ***

SPEED OF PUBLICATION
The journal aims for rapid publication of papers, using using the advance online publication "Early View" to expedite the process: A properly copy-edited and formatted version will be published as "Early View" after the proofs have been corrected. Please help the Editors and publisher avoid delays by providing e-mail address(es), telephone and fax numbers at which author(s) can be contacted.

Should you be planning a Press Release on your article, please get in contact with embomolmed@wiley.com as early as possible, in order to coordinate publication and release dates.

LICENSE AND PAYMENT:

All articles published in EMBO Molecular Medicine are fully open access: immediately and freely available to read, download and share.

EMBO Molecular Medicine charges an article processing charge (APC) to cover the publication costs. You, as the corresponding author for this manuscript, should have already received a quote with the article processing fee separately. Please let us know in case this quote has not been received.

Once your article is at Wiley for editorial production you will receive an email from Wiley's Author Services system, which will ask you to log in and will present you with the publication license form for completion. Within the same system the publication fee can be paid by credit card, an invoice, pro forma invoice or purchase order can be requested.

Payment of the publication charge and the signed Open Access Agreement form must be received before the article can be published online.

PROOFS

You will receive the proofs by e-mail approximately 2 weeks after all relevant files have been sent o our Production Office. Please return them within 48 hours and if there should be any problems, please contact the production office at embopressproduction@wiley.com.

Please inform us if there is likely to be any difficulty in reaching you at the above address at that time. Failure to meet our deadlines may result in a delay of publication.

All further communications concerning your paper proofs should quote reference number EMM-2022-17014-V3 and be directed to the production office at embopressproduction@wiley.com.

EMBO Press Author Checklist

Corresponding Author Name: Pan-Chyr Yang and Chi-Yuan Chen	
Journal Submitted to: EMBO Molecular Medicine	
Manuscript Number: EMM-2022-17014-V2	

USEFUL LINKS FOR COMPLETING THIS FORM
The EMBO Journal - Author Guidelines
EMBO Reports - Author Guidelines
Molecular Systems Biology - Author Guidelines
EMBO Molecular Medicine - Author Guidelines

Reporting Checklist for Life Science Articles (updated January 2022)
This checklist is adapted from Materials Design Analysis Reporting (MDAR) Checklist for Authors. MDAR establishes a minimum set of requirements in transparent reporting in the life sciences (see Statement of Task: 10.31222/osf.io/9sm4x). Please follow the journal's guidelines in preparing your manuscript.
Please note that a copy of this checklist will be published alongside your article.

Abridged guidelines for figures
1. Data
The data shown in figures should satisfy the following conditions:
- the data were obtained and processed according to the field's best practice and are presented to reflect the results of the experiments in an accurate and unbiased manner.
- ideally, figure panels should include only measurements that are directly comparable to each other and obtained with the same assay.
- plots include clearly labeled error bars for independent experiments and sample sizes. Unless justified, error bars should not be shown for technical replicates.
- if n<5, the individual data points from each experiment should be plotted. Any statistical test employed should be justified.
- Source Data should be included to report the data underlying figures according to the guidelines set out in the authorship guidelines on Data Presentation.

2. Captions
Each figure caption should contain the following information, for each panel where they are relevant:
- a specification of the experimental system investigated (eg cell line, species name).
- the assay(s) and method(s) used to carry out the reported observations and measurements.
- an explicit mention of the biological and chemical entity(ies) that are being measured.
- an explicit mention of the biological and chemical entity(ies) that are altered/varied/perturbed in a controlled manner.
- the exact sample size (n) for each experimental group/condition, given as a number, not a range;
- a description of the sample collection allowing the reader to understand whether the samples represent technical or biological replicates (including how many animals, litters, cultures, etc.).
- a statement of how many times the experiment shown was independently replicated in the laboratory.
- definitions of statistical methods and measures:
  - common tests, such as t-test (please specify whether paired vs. unpaired), simple χ2 tests, Wilcoxon and Mann-Whitney tests, can be unambiguously identified by name only, but more complex techniques should be described in the methods section;
  - are tests one-sided or two-sided?
  - are there adjustments for multiple comparisons?
  - exact statistical test results, e.g., P values = x but not P values < x;
  - definition of 'center values' as median or average;
  - definition of error bars as s.d. or s.e.m.

Please complete ALL of the questions below.
Select "Not Applicable" only when the requested information is not relevant for your study.

Materials

Newly Created Materials	Information included in the manuscript?	In which section is the information available? (Reagents and Tools Table, Materials and Methods, Figures, Data Availability Section)
New materials and reagents need to be available; do any restrictions apply?	Yes	Reagents Table, Materials and Methods.

Antibodies	Information included in the manuscript?	In which section is the information available? (Reagents and Tools Table, Materials and Methods, Figures, Data Availability Section)
For **antibodies** provide the following information:<br>- Commercial antibodies: RRID (if possible) or supplier name, catalogue number and or/clone number<br>- Non-commercial: RRID or citation	Yes	Reagents Table, Materials and Methods.

DNA and RNA sequences	Information included in the manuscript?	In which section is the information available? (Reagents and Tools Table, Materials and Methods, Figures, Data Availability Section)
**Short novel DNA or RNA including primers, probes**: provide the sequences.	Yes	Reagents Table, Materials and Methods.

Cell materials	Information included in the manuscript?	In which section is the information available? (Reagents and Tools Table, Materials and Methods, Figures, Data Availability Section)
**Cell lines:** Provide species information, strain. Provide accession number in repository **OR** supplier name, catalog number, clone number, and/**OR** RRID.	Yes	Materials and Methods.
**Primary cultures:** Provide species, strain, sex of origin, genetic modification status.	Not Applicable	Not Applicable.
Report if the cell lines were recently **authenticated** (e.g., by STR profiling) and tested for mycoplasma contamination.	Yes	Materials and Methods.

Experimental animals	Information included in the manuscript?	In which section is the information available? (Reagents and Tools Table, Materials and Methods, Figures, Data Availability Section)
**Laboratory animals or Model organisms:** Provide species, strain, sex, age, genetic modification status. Provide accession number in repository **OR** supplier name, catalog number, clone number, **OR** RRID.	Yes	Materials and Methods.
**Animal observed in or captured from the field:** Provide species, sex, and age where possible.	Not Applicable	Not Applicable.
Please detail **housing and husbandry conditions**.	Yes	Materials and Methods.

Plants and microbes	Information included in the manuscript?	In which section is the information available? (Reagents and Tools Table, Materials and Methods, Figures, Data Availability Section)
**Plants:** provide species and strain, ecotype and cultivar where relevant, unique accession number if available, and source (including location for collected wild specimens).	Not Applicable	Not Applicable.
**Microbes:** provide species and strain, unique accession number if available, and source.	Not Applicable	Not Applicable.

Human research participants	Information included in the manuscript?	In which section is the information available? (Reagents and Tools Table, Materials and Methods, Figures, Data Availability Section)
If collected and within the bounds of privacy constraints report on age, sex and gender or ethnicity for all study participants.	Yes	Table 3, Materials and Methods.

Core facilities	Information included in the manuscript?	In which section is the information available? (Reagents and Tools Table, Materials and Methods, Figures, Data Availability Section)
If your work benefited from core facilities, was their service mentioned in the acknowledgments section?	Yes	Acknowledgments

Design

Study protocol	Information included in the manuscript?	In which section is the information available? (Reagents and Tools Table, Materials and Methods, Figures, Data Availability Section)
If study protocol has been **pre-registered, provide DOI in the manuscript**. For clinical trials, provide the trial registration number **OR** cite DOI.	Not Applicable	Not Applicable.
Report the **clinical trial registration number** (at ClinicalTrials.gov or equivalent), where applicable.	Not Applicable	Not Applicable.

Laboratory protocol	Information included in the manuscript?	In which section is the information available? (Reagents and Tools Table, Materials and Methods, Figures, Data Availability Section)
Provide DOI OR other citation details if **external detailed step-by-step protocols** are available.	Not Applicable	Not Applicable.

Experimental study design and statistics	Information included in the manuscript?	In which section is the information available? (Reagents and Tools Table, Materials and Methods, Figures, Data Availability Section)
Include a statement about **sample size** estimate even if no statistical methods were used.	Yes	Figure, Materials and Methods.
Were any steps taken to minimize the effects of subjective bias when allocating animals/samples to treatment (e.g. **randomization procedure**)? If yes, have they been described?	Not Applicable	Not Applicable.
Include a statement about **blinding** even if no blinding was done.	Yes	Materials and Methods.
Describe **inclusion/exclusion criteria** if samples or animals were excluded from the analysis. Were the criteria pre-established?<br><br>If sample or data points were omitted from analysis, report if this was due to attrition or intentional exclusion and provide justification.	Not Applicable	Not Applicable.
For every figure, are **statistical tests** justified as appropriate? Do the data meet the assumptions of the tests (e.g., normal distribution)? Describe any methods used to assess it. Is there an estimate of variation within each group of data? Is the variance similar between the groups that are being statistically compared?	Yes	Materials and Methods, Figure.

Sample definition and in-laboratory replication	Information included in the manuscript?	In which section is the information available? (Reagents and Tools Table, Materials and Methods, Figures, Data Availability Section)
In the figure legends: state number of times the experiment was **replicated** in laboratory.	Yes	Figure legends.
In the figure legends: define whether data describe **technical or biological replicates**.	Yes	Figure legends.

Ethics

Ethics	Information included in the manuscript?	In which section is the information available? (Reagents and Tools Table, Materials and Methods, Figures, Data Availability Section)
Studies involving **human participants**: State details of **authority granting ethics approval** (IRB or equivalent committee(s), provide reference number for approval.	Yes	Materials and Methods.
Studies involving **human participants**: Include a statement confirming that **informed consent** was obtained from all subjects and that the experiments conformed to the principles set out in the WMA Declaration of Helsinki and the Department of Health and Human Services Belmont Report.	Yes	Materials and Methods.
Studies involving **human participants:** For publication of **patient photos**, include a statement confirming that consent to publish was obtained.	Not Applicable	Not Applicable.
Studies involving experimental **animals**: State details of **authority granting ethics approval** (IRB or equivalent committee(s), provide reference number for approval. Include a statement of compliance with ethical regulations.	Yes	Materials and Methods.
Studies involving **specimen and field samples:** State if relevant **permits** obtained, provide details of authority approving study; if none were required, explain why.	Not Applicable	Not Applicable.

Dual Use Research of Concern (DURC)	Information included in the manuscript?	In which section is the information available? (Reagents and Tools Table, Materials and Methods, Figures, Data Availability Section)
Could your study fall under dual use research restrictions? Please check biosecurity documents and list of **select agents and toxins** (CDC): https://www.selectagents.gov/sat/list.htm	Not Applicable	Not Applicable.
If you used a select agent, is the security level of the lab appropriate and reported in the manuscript?	Not Applicable	Not Applicable.
If a study is subject to dual use research of concern regulations, is the name of the **authority granting approval and reference number** for the regulatory approval provided in the manuscript?	Not Applicable	Not Applicable.

Reporting

The MDAR framework recommends adoption of discipline-specific guidelines, established and endorsed through community initiatives. Journals have their own policy about requiring specific guidelines and recommendations to complement MDAR.

Adherence to community standards	Information included in the manuscript?	In which section is the information available? (Reagents and Tools Table, Materials and Methods, Figures, Data Availability Section)
State if relevant guidelines or checklists (e.g., **ICMJE, MIBBI, ARRIVE, PRISMA**) have been followed or provided.	Not Applicable	Not Applicable.
For **tumor marker prognostic studies,** we recommend that you follow the **REMARK** reporting guidelines (see link list at top right). See author guidelines, under 'Reporting Guidelines'. Please confirm you have followed these guidelines.	Not Applicable	Not Applicable.
For **phase II and III randomized controlled trials**, please refer to the **CONSORT** flow diagram (see link list at top right) and submit the CONSORT checklist (see link list at top right) with your submission. See author guidelines, under 'Reporting Guidelines'. Please confirm you have submitted this list.	Not Applicable	Not Applicable.

Data Availability

Data availability	Information included in the manuscript?	In which section is the information available? (Reagents and Tools Table, Materials and Methods, Figures, Data Availability Section)
Have **primary datasets** been deposited according to the journal's guidelines (see 'Data Deposition' section) and the respective accession numbers provided in the Data Availability Section?	Yes	Materials and Methods.
Were **human clinical and genomic datasets** deposited in a public access-controlled repository in accordance to ethical obligations to the patients and to the applicable consent agreement?	Not Applicable	Not Applicable.
Are **computational models** that are central and integral to a study available without restrictions in a machine-readable form? Were the relevant accession numbers or links provided?	Not Applicable	Not Applicable.
If publicly available data were reused, provide the respective **data citations in the reference list**.	Not Applicable	Not Applicable.
